# Membrane-targeting antibacterial isoniazid schiff base against *S. aureus* and biofilms 

**DOI:** 10.3389/fchem.2025.1654358

**Published:** 2025-09-09

**Authors:** Yaguang Liu, Lianzhi Hu, Binbin Liu, Zheng Qu

**Affiliations:** Pharmacy Department, The Second Hospital of Qinhuangdao, Qinhuangdao, China

**Keywords:** isoniazid, schiff base, antibacterial activity, anti biofilm, anti inflammatory

## Abstract

**Introduction:**

Building upon previous research, this study focuses on the replication and evaluation of a series of hydrazone derivatives derived from isoniazid.

**Methods:**

The lead compound, identified as C5, was assessed for its antibacterial activity against Gram-positive bacteria, notably Staphylococcus aureus ATCC 29213. Its hemolytic potential, cytotoxicity (against VERO cells), and ability to induce resistance were evaluated. Mechanistic studies included assays for membrane depolarization (using DiSC35 fluorescence), membrane integrity (via SYTOX Green uptake), measurement of intracellular ATP levels, and detection of reactive oxygen species (ROS). Additional investigations examined its effect on LPS-induced NO/TNF-α release in macrophages and its activity against *S. aureus* biofilms.

**Results:**

Compound C5 exhibited potent antibacterial activity (MIC = 16 μg/mL against *S. aureus* ATCC 29213). It demonstrated no hemolysis and low cytotoxicity (IC50 > 128 μg/mL). A time-kill assay achieved complete eradication of *S. aureus* within 16 hours at 8× MIC, and the compound showed a low tendency to induce resistance. The mechanistic studies revealed that C5 disrupts the bacterial membrane, causing depolarization, loss of integrity, and leakage of proteins/DNA. It also induced ROS accumulation and significantly reduced ATP levels. Furthermore, C5 suppressed LPS-induced NO/TNF-α release in macrophages (p < 0.01) and inhibited/disrupted *S. aureus* biofilms.

**Discussion:**

These results demonstrate that C5 possesses a multifunctional mechanism of action, combining direct bactericidal activity through membrane targeting with anti-biofilm efficacy and immunomodulatory properties. This multifaceted profile highlights its strong potential as a promising candidate for combating resistant bacterial infections.

## 1 Introduction

Antibiotic resistance in Gram-positive bacteria has become a critical global public health threat (Asenjo et al., 2021). The latest World Health Organization (WHO) report indicates that methicillin-resistant *Staphylococcus aureus* (MRSA) now surpasses HIV infection in mortality rates (Lakhundi and Zhang, 2018). Moreover, the pipeline for novel traditional antibiotics is nearing depletion: among antibacterial new molecular entities currently entering Phase I clinical trials, only approximately one-third target Gram-positive bacteria. Furthermore, the vast majority are derivatives of existing antibiotics, lacking truly groundbreaking mechanisms of action (Mohr, 2016). While traditional antibiotics like vancomycin remain the ‘last line of defense’ against MRSA infections, their nephrotoxicity and the rising prevalence of resistance (exemplified by the emergence of vancomycin-resistant *S. aureus* VRSA strains) underscore the urgent need for novel antibacterial agents with distinct mechanisms of action (Cheung et al., 2021; García-Castro et al., 2023).

Against this backdrop, isoniazid (INH), a first-line tuberculosis drug, has garnered significant interest due to its unique hydrazine (-NHNH_2_) pharmacophore (Ridahunlang et al., 2023). However, the antibacterial spectrum of isoniazid is relatively narrow, exhibiting high efficacy primarily against Mycobacteria, while its activity against many common Gram-positive bacteria (such as *Staphylococcus aureus*, *Streptococcus* pneumoniae, etc.) and Gram-negative bacteria is limited (Bhowmik et al., 2023; Poulton and Rock, 2022). This is largely attributed to its strong hydrophilicity, which hinders effective penetration through the dense peptidoglycan layer of Gram-positive bacteria as well as the outer membrane barrier of Gram-negative bacteria. However, the highly reactive hydrazine moiety within the INH molecule provides an ideal platform for structural modification (Sodré-Alves et al., 2024). Studies demonstrate that constructing Schiff bases via aldehyde-amine condensation can confer amphiphilic character to the resulting molecules. This modification enhances penetration through the cell membranes of Gram-positive bacteria while simultaneously evading recognition by efflux pumps (Hong et al., 2025). Consequently, developing novel Schiff base derivatives based on the INH scaffold represents not only a rational strategy to overcome its inherent antibacterial spectrum limitations but also an innovative approach to combat infections caused by drug-resistant Gram-positive bacteria.

Schiff bases possess diverse biological activities, including antibacterial, anticancer, and antioxidant effects, making them highly valuable research targets (Udhayakumari and Inbaraj, 2020; Rana et al., 2024; Presenjit et al., 2024). The unique structure of the Schiff base linkage (-C=N-) offers a triple advantage in antibacterial drug design: Membrane Targeting: The electron delocalization characteristic of the imine bond facilitates molecular intercalation into the bacterial phospholipid bilayer, disrupting membrane potential through electrostatic interactions (Fontana et al., 2022). Metal Chelation Capacity: The lone pair of electrons on the nitrogen atom enables the chelation of metal ions such as Mg^2+^ and Zn^2+^, interfering with the function of metalloenzymes (e.g., DNA polymerase, peptide deformylase) (Kaur et al., 2023). ROS-Inducing Effect: Schiff bases substituted with nitro/hydroxyl groups can act as electron shuttles, disrupting respiratory chain complex I and triggering a burst of reactive oxygen species (ROS) (Barua et al., 2024). It is worth noting that Schiff bases are important compounds in synthetic processes and drug discovery (Han et al., 2025; Klika et al., 2022; Kopka et al., 2019).

**FIGURE 1:**
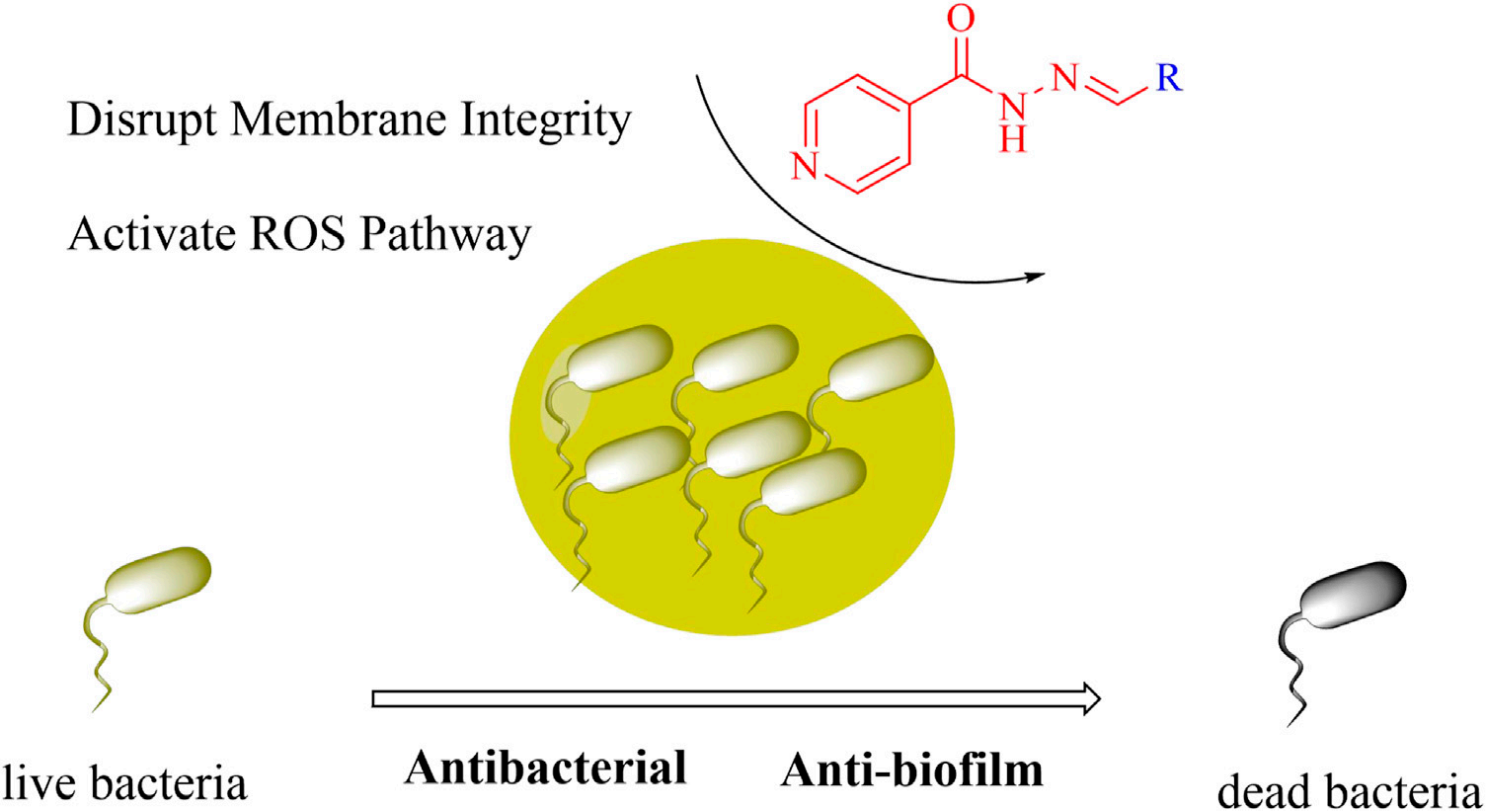
Antibacterial mechanism diagram of Isoniazid Schiff Base derivatives.

Therefore, this study adopted a molecular hybridization strategy. Condensing isoniazid with aromatic aldehydes to form Schiff bases. The introduced aromatic aldehydes serve as hydrophobic groups to enhance lipophilicity, thus improving the ability to penetrate the thick peptidoglycan layer of Gram-positive bacteria. This approach led to the discovery of compound **C5** (*N*'-(2-hydroxy-5-nitrobenzylidene)isonicotinohydrazide), which exhibits potent antibacterial activity. Compound **C5** rapidly eradicates *Staphylococcus aureus* by disrupting cell membrane integrity and activating the endogenous ROS pathway (Figure 1). This work provides a chemical entity (NCE) for developing drugs against drug-resistant Gram-positive bacteria. Furthermore, it establishes a theoretical foundation for the rational design of Schiff base-based antibacterial agents.

## 2 Results and discussion

### 2.1 Chemical synthesis

**SCHEME 1:**
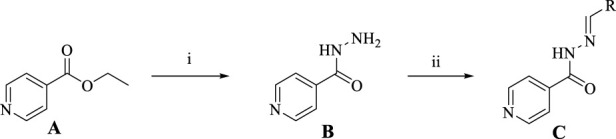
Synthesis of Isoniazid derivatives. Conditions and reagents: (i) ethanol, NH_2_NH_2_, reflux, yield 86%; (ii) ethanol, different aldehyde groups, reflux, yield 81%–92%.

Building on established synthetic approaches, this process employs ethyl isonicotinate as the starting material, as outlined in Scheme 1 (Backes et al., 2015; Ji et al., 2018; Rouzi et al., 2024). Hydrazinolysis with hydrazine hydrate in ethanol solvent affords the key intermediate, isoniazid. This step features simple operation under mild, well-controlled conditions, delivering isoniazid in 86% isolated yield with high efficiency. Subsequently, the reactive hydrazine group of isoniazid undergoes condensation with structurally diverse aldehyde derivatives in ethanol. This transformation also proceeds under mild conditions with excellent reaction compatibility, successfully yielding varying target products C. For compound B, the signal for the hydrazinyl proton was observed at δ 12.06 (s, 1H), which is consistent with literature values. For compound C, the set of signals in the aromatic region (δ 9.0-7.0) for the phenyl protons also agreed well with reported data (Backes et al., 2015; Ji et al., 2018; Rouzi et al., 2024). With the exception of compound **C7**, all other compounds have been previously synthesized and were not first developed in this study (Backes et al., 2015; Ji et al., 2018; Rouzi et al., 2024). Critically, all intermediates and final products C are purified to high purity via straightforward recrystallization, eliminating the need for tedious, time-consuming, and costly column chromatography. This purification strategy significantly streamlines the workflow, enhances process economy, and improves scalability, collectively highlighting the substantial potential of this route for industrial-scale applications.

### 2.2 Determination of minimum inhibitory concentration

Reportedly, some Schiff base derivatives exhibit promising *in vitro* antibacterial activity (Hong et al., 2025). Therefore, this study employed the broth microdilution method to determine the *in vitro* antibacterial activity (MIC) against the following strains: Gram-positive bacteria: *Staphylococcus aureus* ATCC 29213, *Staphylococcus aureus* ATCC 43300, *Staphylococcus aureus* ATCC 33731, *Staphylococcus aureus* MRSA2, *Bacillus Subtilis* ATCC6633. Gram-negative bacteria: *Escherichia coli* ATCC 25922, *Salmonella enterica* serovar Enteritidis SM012. The antibacterial results for all compounds are summarized in Table 1. Among the tested compounds: **C5** (16 μg/mL) exhibited inhibitory activity against all tested *S. aureus* strains. **C1** (64 μg/mL) also inhibited all tested *S. aureus* strains. The remaining compounds demonstrated poor antibacterial activity, likely attributable to their overall poor solubility.

**TABLE 1:** The antibacterial activity of Isoniazid derivatives.

MIC ^a^ (μg/mL)								
Compounds	R	*E. coli* ATCC 25922	*S. enteritidis* SM012	*S. aureus* ATCC 29213	*S. aureus* ATCC 43300	*S. aureus* ATCC 33731	*S. aureus* MRSA2	*B.* Subtilis ATCC6633
Vancomycin^b^	-	-	-	1	1	1	1	1
Enrofloxacin^c^	-	0.0625	0.0625	-	-	-	-	-
**C1**	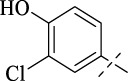	>256	>256	64	64	64	128	128
**C2**	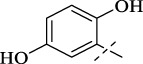	>256	>256	>256	>256	>256	>256	>256
**C3**	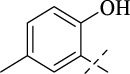	>256	>256	>256	>256	>256	>256	>256
**C4**	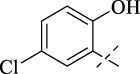	>256	>256	>256	>256	>256	>256	>256
**C5**	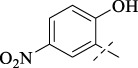	>256	>256	16	16	16	16	16
**C6**	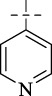	>256	>256	>256	>256	>256	>256	>256
**C7**	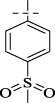	>256	>256	128	128	128	128	128
**C8**	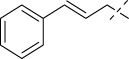	>256	>256	>256	>256	>256	>256	>256
**C9**	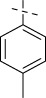	>256	>256	>256	>256	>256	>256	>256
**C5**0	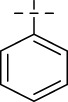	>256	>256	128	128	128	256	256
**C11**	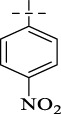	>256	>256	>256	>256	>256	>256	>256
**C12**	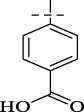	>256	>256	>256	>256	>256	>256	>256
**C13**	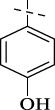	>256	>256	>256	>256	>256	>256	>256
**C14**	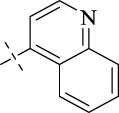	>256	>256	>256	>256	>256	>256	>256
**C15**	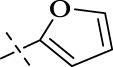	256	128	128	128	128	128	256
**C16**	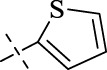	>256	>256	>256	>256	>256	>256	>256

^a^ The minimum inhibitory concentration (MIC) is the lowest concentration that completely inhibits microbial growth after 16–24 h. Each experiment was repeated three times.

^b^ vancomycin is a clinical drug against Gram-positive bacteria.

^c^ Enrofloxacin is a broad-spectrum quinolone-based antibiotic.

### 2.3 Time-killing curve determinations and drug resistance study

**FIGURE 2:**
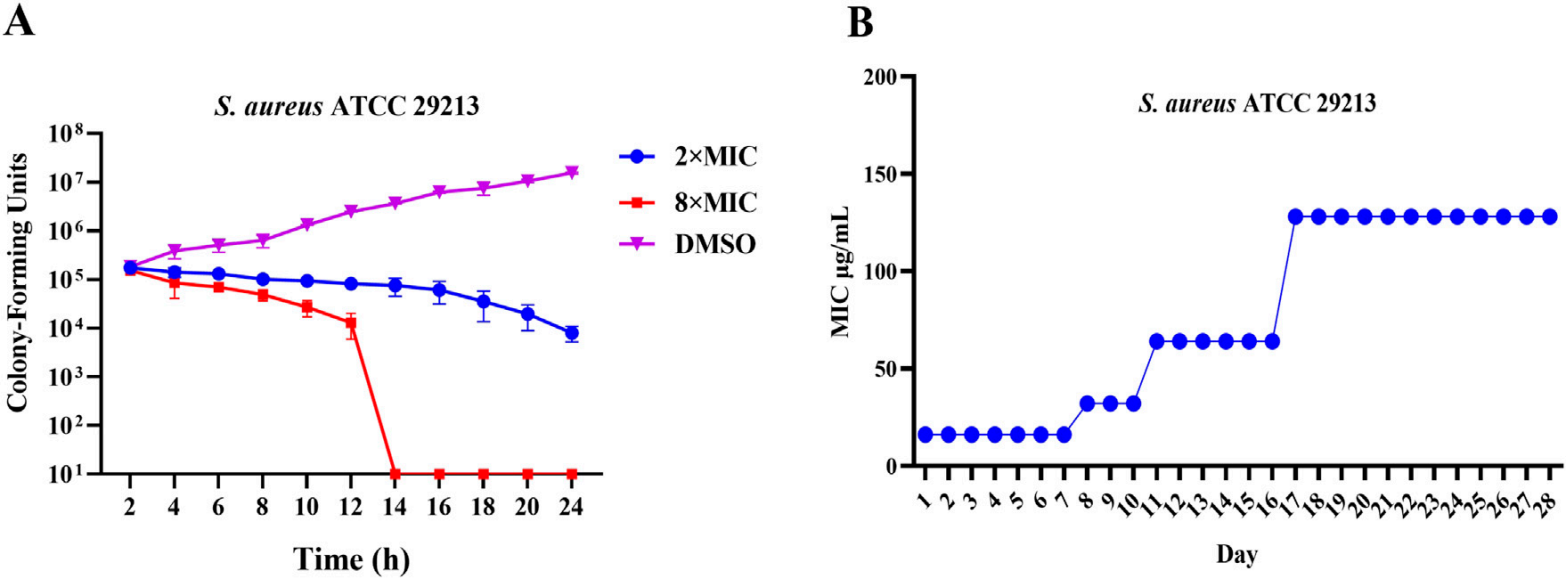
**(A)** Time-kill kinetics of **C5** against *S. aureus* ATCC 29213. **(B)** Resistance development of **C5**. Data are presented as means ± SEM (Standard Error of Mean) from three independent experiments.

To evaluate the bactericidal efficacy of **C5** against S. *aureus* ATCC 29213, we determined the time-kill kinetics of the compound by enumerating bacterial colonies at various time points, using dimethyl sulfoxide (DMSO) as the negative control (Xiao et al., 2024). As shown in Figure 2A, the growth of *S. aureus* ATCC 29213 was completely inhibited at 4 × MIC. Schiff bases exhibit a low propensity for resistance development due to their multi-target mechanism of action and membrane-disrupting effects. Consistent with this, resistance development studies demonstrated a low spontaneous resistance frequency for **C5** against *S*. *aureus* ATCC 29213. As depicted in Figure 2B, after 28 serial passages, the MIC value for *S. aureus* ATCC 29213 increased by no more than 8-fold. These results indicate that **C5** effectively kills bacteria while minimizing the development of resistance.

### 2.4 The toxicity of the compounds

**FIGURE 3:**
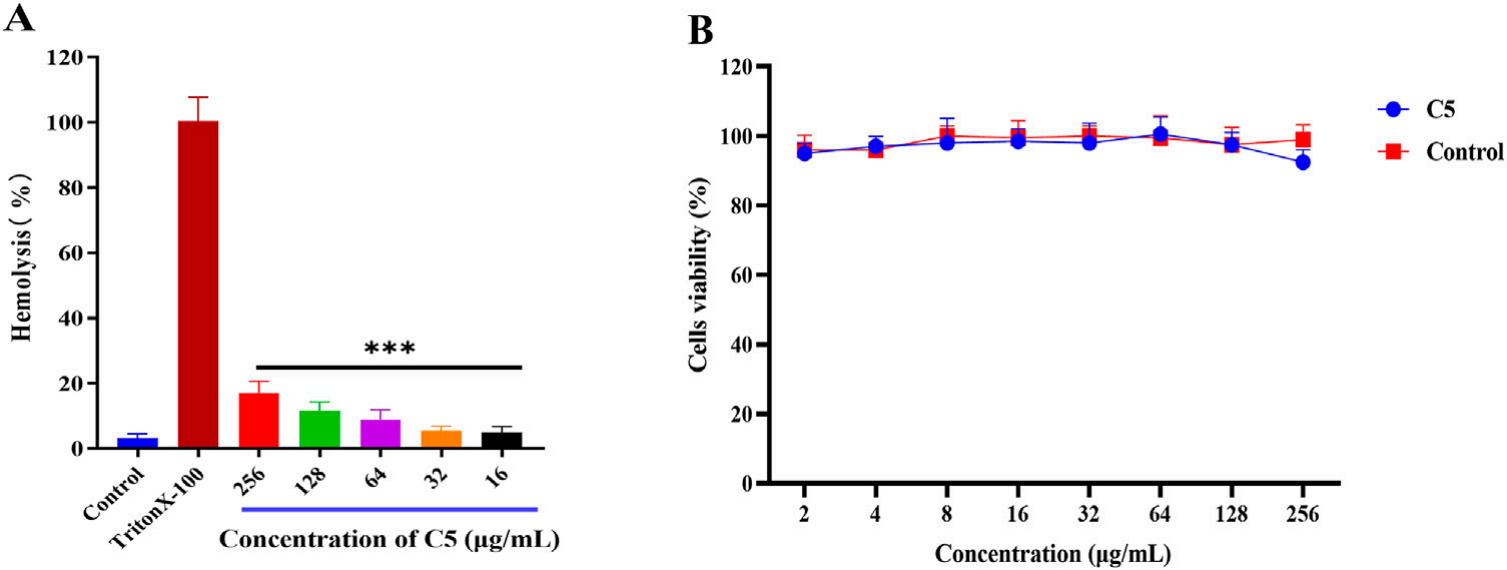
**(A)** Percentage of hemolysis of rabbit blood cells at various **C5** concentrations, The values represented by the bars from left to right are: 3, 100, 17, 11, 9, 5, and 5. **(B)** Cytotoxicity of compound **C5** against Vero cells after 24 h. Difference is considered significant at ^*^p < 0.05, ^**^p < 0.01, ^***^p < 0.001.

To evaluate compound safety, hemolysis assays were first performed for all test compounds. A 1% Triton X-100 solution served as the positive control, and sterile PBS was used as the negative control. As shown in Figure 3A, no hemolysis was observed for compound **C5** across the concentration range of 2–256 μg/mL. This indicates that **C5** exhibits no hemolytic activity against rabbit erythrocytes at concentrations effective for its antibacterial action. Subsequently, the cytotoxicity of the active compound **C5** against African green monkey kidney (VERO) cells was assessed using the CCK-8 assay.The results (Figure 3B) demonstrate that **C5** exhibited no cytotoxicity towards VERO cells at concentrations up to 256 μg/mL.

### 2.5 Antimicrobial mechanism investigation

#### 2.5.1 Membrane depolarization and permeabilization assay

Studies indicate that the antibacterial activity of Schiff base compounds is associated with their hydrophobic interactions (Caldés et al., 2013; Hong et al., 2025). Based on the Schiff base group and hydrophobic characteristics inherent in compound **C5**’s structure, we hypothesized that it likely exerts its antibacterial effect by targeting the bacterial cell membrane. The specific mechanism may involve inducing alterations in membrane depolarization and permeability. To investigate the direct impact of **C5** on the bacterial membrane, this study employed fluorescent probes: The cationic dye 3,3′-dipropylthiadicarbocyanine iodide (DiSC35) was used to monitor changes in bacterial membrane potential (depolarization). The nucleic acid stain SYTOX Green, which cannot penetrate intact cell membranes, was utilized to assess changes in membrane permeability (integrity), evaluating **C5**’s disruptive effect on membrane function.

**FIGURE 4:**
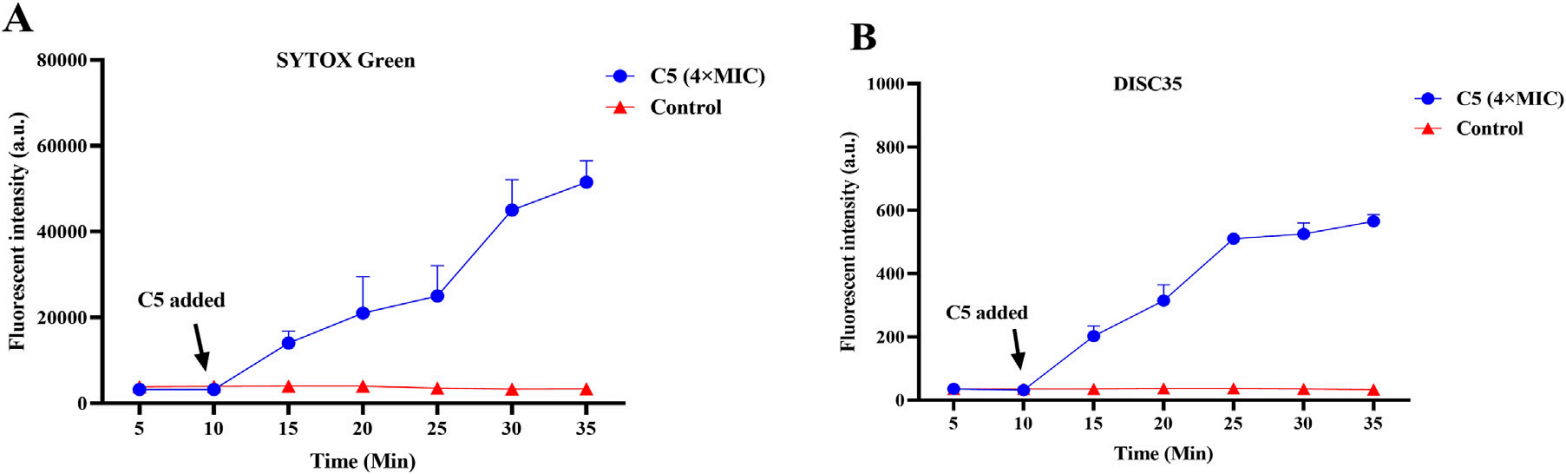
**(A)** Cytoplasmic membrane permeabilization by **C5** assessed using SYTOX Green uptake. **(B)** Cytoplasmic membrane depolarization by **C5** measured with the DiSC35 probe. The blank control was bacteria without compound treatment. Data are presented as means ± SEM from three independent experiments.

Within 10 min of adding compound **C5**, a sustained increase in fluorescence intensity was observed in suspensions of *S. aureus* ATCC 29213 pre-loaded with either the DiSC35 or SYTOX Green probes (Figures 4A,B). When **C5** reached concentrations of 4 × MIC or 32 × MIC, the fluorescence intensity of the bacterial mixtures at 35 min was significantly enhanced compared to the initial value. In contrast, the fluorescence intensity of the blank control (without **C5**) remained stable. These findings demonstrate that **C5** disrupts the polarized state of the bacterial cell membrane (i.e., the distribution of positive and negative charges across the membrane), leading to increased membrane permeability. In conclusion, **C5** exerts its bactericidal effect by mediating membrane damage through alterations in the polarization state and permeability of the bacterial cell membrane.

#### 2.5.2 Intracellular reactive oxygen species (ROS) and ATP

**FIGURE 5:**
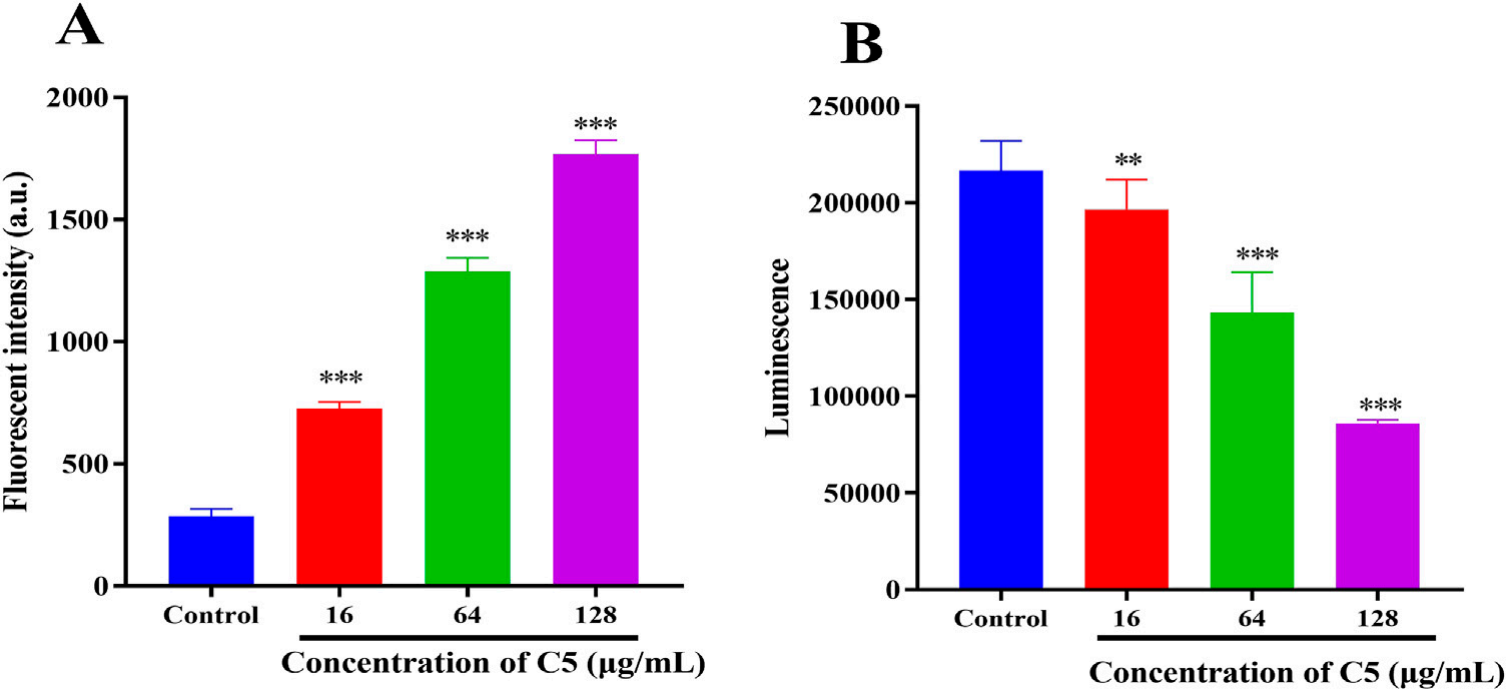
**(A)** Effect of **C5** treatment on reactive oxygen species (ROS) production in *S*. *aureus* ATCC 29213. **(B)** Effect of **C5** treatment on intracellular ATP levels in *S*. *aureus* ATCC 29213. ^*^p < 0.05, ^**^p < 0.01, ^***^p < 0.001. Data are presented as means ± SEM from three independent experiments.

During antibiotic treatment, disruption of membrane equilibrium often leads to the accumulation of ROS, a common mechanism of action for bactericidal antibiotics (Tu et al., 2022). Furthermore, membrane depolarization is also linked to ROS generation (Hong et al., 2025). Therefore, we examined changes in ROS accumulation in bacteria following treatment with compound **C5**. Within 30 min, a significant increase in ROS levels was observed in the **C5**-treated group (Figure 5A). The elevation in bacterial ROS levels corresponded with an increase in the proportion of dead bacteria. Studies indicate a strong correlation between the bactericidal effects of antibiotics and enhanced bacterial respiratory activity (Dörner et al., 2024; Islam and Reid, 2024). Consequently, we assessed the impact of **C5** treatment on intracellular ATP levels in bacteria. As shown in Figure 5B, intracellular ATP levels decreased significantly following treatment with **C5**.

#### 2.5.3 Leakage of proteins and DNA

**FIGURE 6:**
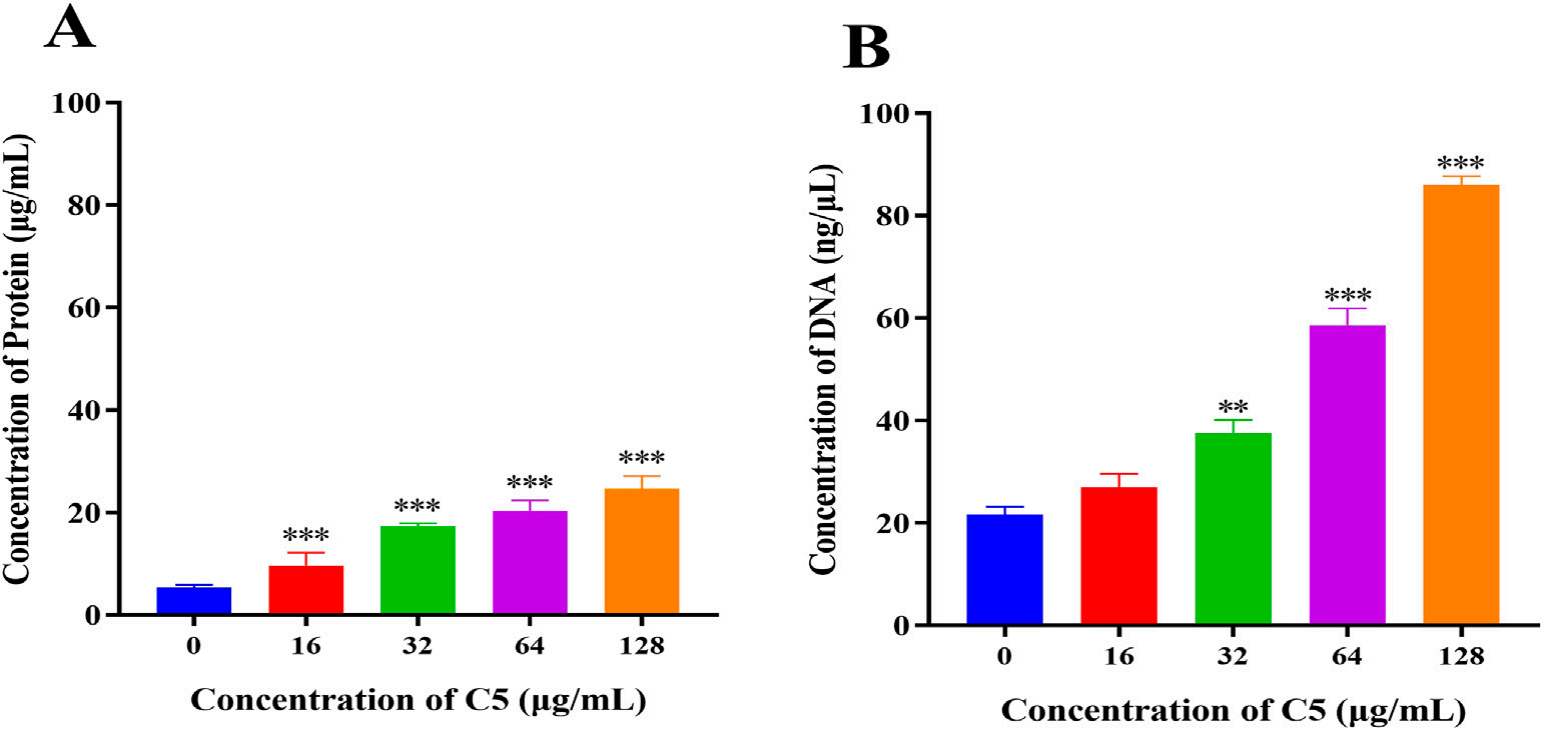
**(A)** Protein leakage caused by the treatment of C5 on *S. aureus* ATCC 29213. **(B)** DNA leakage resulting from the treatment of C5 on *S. aureus* ATCC 29213. ^*^p < 0.05, ^**^p < 0.01, ^***^p < 0.001. Data are presented as means ± SEM from three independent experiments.

To further evaluate the impact of compound **C5** on bacterial membrane integrity, we measured changes in the concentration of proteins and DNA in the extracellular culture medium of *S*. *aureus* ATCC 29213 following treatment with different concentrations of **C5**. The results demonstrated that compared to the blank control group, the concentrations of extracellular proteins and DNA were significantly elevated in the **C5**-treated bacterial suspensions. This effect occurred in a dose-dependent manner (Figures 6A,B). These findings directly demonstrate that **C5** disrupts the cell membrane integrity of *S. aureus* ATCC 29213, leading to the leakage of intracellular contents (proteins and DNA).

### 2.6 The anti-inflammatory activity of the compounds

**FIGURE 7:**
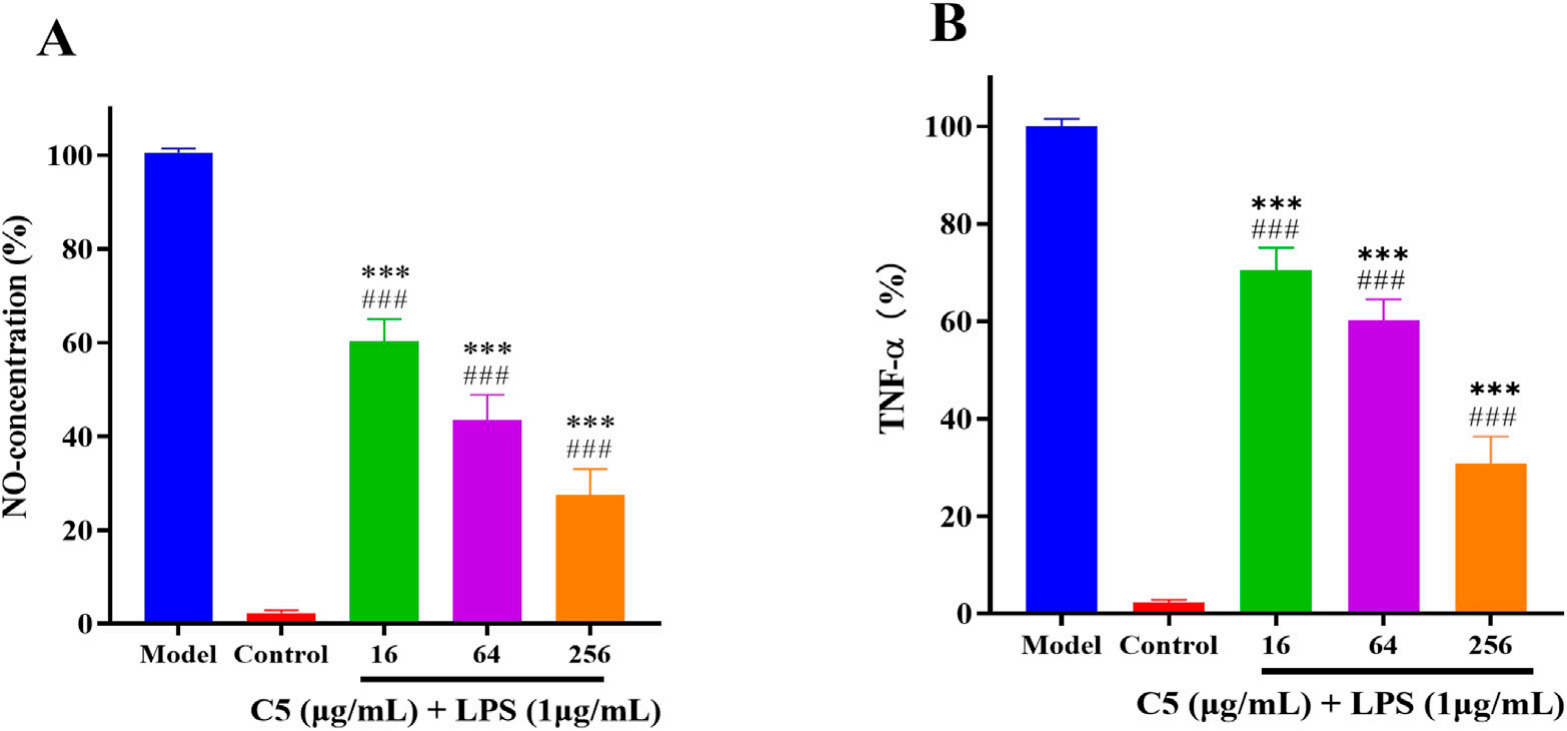
Anti-inflammatory activity of the C5 compounds in RAW 264.7 macrophage cells was evaluated in the LPS-enhanced leukocyte migration assay. **(A)** C5 affects the level of NO. **(B)** C5 affects the level of TNF-α. Compared with the LPS model group, ^*^p < 0.05, ^**^p < 0.01, ^***^p < 0.001; ^###^p < 0.001 vs. control group. Data are presented as means ± SEM from three independent experiments.

Inflammation commonly accompanies infections. Given that Schiff Base derivatives have been demonstrated to possess anti-inflammatory effects, we further evaluated the impact of compound **C5** on the levels of inflammatory factors NO and TNF-α (Hu et al., 2022). As shown in Figure 7, compared to the control group, LPS stimulation alone significantly increased the production of NO and TNF-α in RAW 264.7 cells. However, treatment with compound **C5** significantly suppressed this production. At a concentration as low as 64 μg/mL, compound **C5** effectively reduced the generation of both NO and TNF-α.

### 2.7 Inhibitory effects towards *S. Aureus* biofilm formation

**FIGURE 8:**
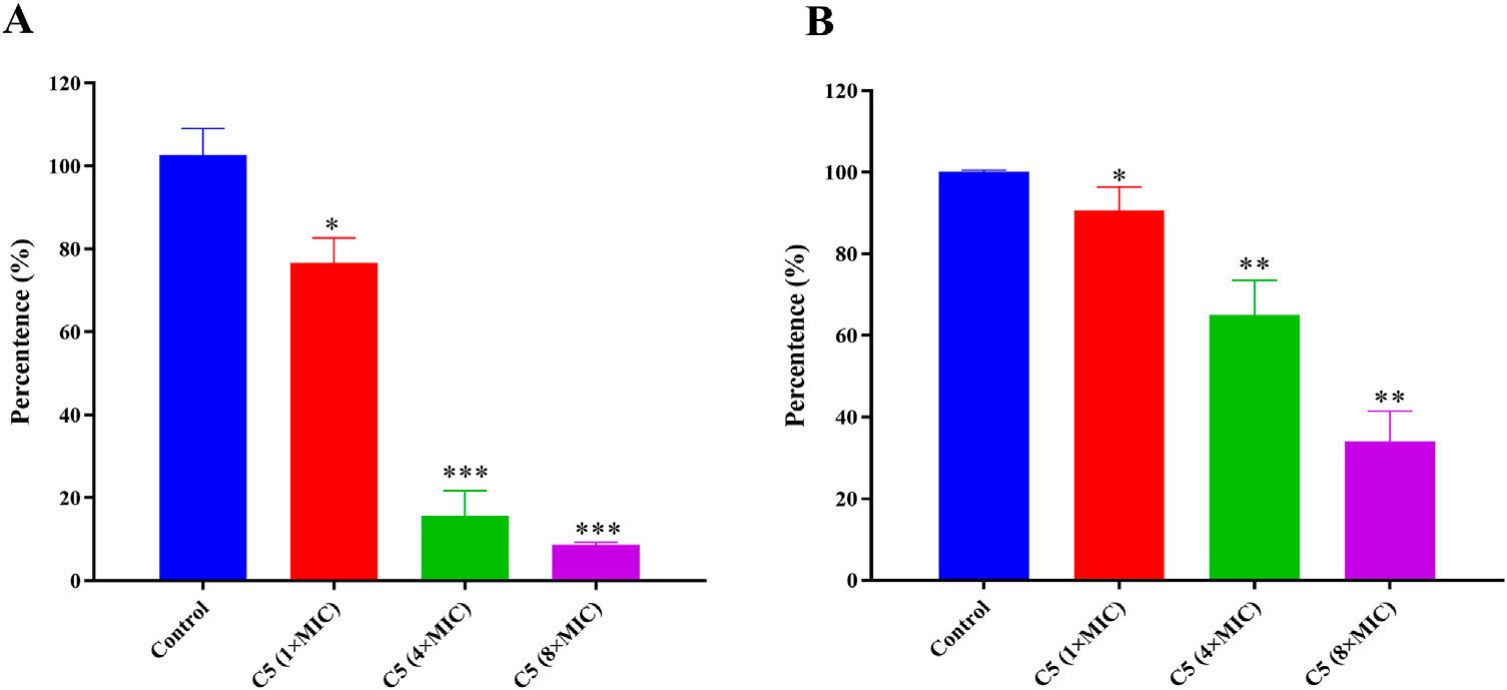
**(A)** Inhibition rate of C5 on *S. aureus* ATCC 29213 biofilm formation. **(B)** Biofilm dispersion of C5 on *S. aureus* ATCC 29213 biofilm. Difference is considered significant at ^*^p < 0.05, ^**^p < 0.01, vs. control group. Data are presented as means ± SD from three independent experiments.

Over 80% of chronic bacterial infections in humans are associated with biofilms. Biofilms are structured communities of bacteria encased within a protective extracellular polymeric matrix, exhibiting significantly enhanced tolerance to antimicrobial agents and host defense systems. In contexts such as medical devices (e.g., catheters, implants), chronic wounds, and cystic fibrosis lungs, biofilm-associated infections are characterized by their persistent, recurrent, and recalcitrant nature (Sirinirund et al., 2023; Vyas et al., 2020). Consequently, there is an urgent need to develop agents capable of effectively preventing biofilm formation and eradicating established biofilms. Building upon this, we investigated the ability of compound **C5** to inhibit biofilm formation by *S*. *aureus* ATCC 29213. Quantitative analysis of biofilms was performed using the crystal violet assay. Figure 8A illustrates the inhibitory effects of **C5** at various concentrations. **C5** exhibited dose-dependent inhibition of *S. aureus* ATCC 29213 biofilm formation: 23% (16 μg/mL, 1 × MIC), 84% (64 μg/mL, 4 × MIC), and 91% (256 μg/mL, 8 × MIC). Subsequently, we further evaluated the eradication efficacy of **C5** against pre-formed *S. aureus* ATCC 29213 biofilms (Figure 8B). **C5** effectively disrupted established biofilms with eradication rates of 10% (1 × MIC), 35% (4 × MIC), and 66% (8 × MIC), confirming its potency against biofilm-embedded *S. aureus*.

## 3 Conclusion

Based on compounds synthesized by other researchers, this study discovered the antibacterial activity of the isoniazid-based Schiff base agent, featuring a lead compound (MIC = 16 μg/mL) with outstanding properties: potent and rapid bactericidal activity achieving complete eradication within 16 h at 8 × MIC coupled with low resistance potential; a defined multi-mechanistic antibacterial action involving disruption of bacterial membrane integrity (as confirmed by DiSC35) depolarization and SYTOX Green uptake) leading to intracellular content leakage, interference with energy metabolism via ATP depletion, and induction of oxidative stress through ROS accumulation; significant anti-biofilm efficacy; a unique anti-inflammatory function suppressing the production of key macrophage mediators NO and TNF-α; and an excellent safety profile demonstrating no hemolysis and extremely low mammalian cytotoxicity (IC_50_ > 128 μg/mL). This combined “antibacterial–anti-inflammatory–anti-biofilm” triple synergistic effect positions the compound as a highly promising multifunctional candidate for combating drug-resistant staphylococcal infections.

## 4 Experimental section

### 4.1 Chemically synthetical experiments

All chemicals were of reagent grade or higher and used as received from Adamas without further purification. Solvents were employed as supplied or dried over molecular sieves when necessary. Column chromatography was performed on silica gel (100–200 mesh, Qingdao Ocean Chemical). Reaction progress was monitored by TLC on silica gel GF254 plates (Yantai Jiangyou). ^1^H NMR (400 MHz) and ^13^C NMR (100 MHz) spectra were recorded on a Bruker Avance 400 spectrometer, with chemical shifts reported relative to residual solvent signals (CDCl_3_: δH 7.26 ppm, δC 77.16 ppm). High-resolution mass spectra (HRMS) were acquired on an AB Sciex TripleTOF 5600+ instrument using electrospray ionization (ESI).

#### 4.1.1 Isonicotinohydrazide (B)

Ethyl isonicotinate (1 mmol, 151 mg) was dissolved in anhydrous ethanol. Hydrazine hydrate (3 mmol) was then added, and the solution was heated to reflux at 80 °C for 8 h. After completion of the reaction, isoniazid was obtained by recrystallization from ethanol.

119 mg, Yield, 86%. White solid powder. M.P. 171 °C–173 °C. ^1^H NMR (400 MHz, DMSO-*d*_6_) δ 10.07 (s, 1H), 8.71–8.64 (m, 3H), 7.74–7.68 (m, 3H). ^13^C NMR (101 MHz, DMSO-*d*_6_) δ 164.54, 150.63, 140.72, 121.49. TOF-MS, m/z: [M + H]^+^, calcd. for C_6_H_8_N_3_O^+^, 138.0667, found: 138.0669.

#### 4.1.2 *N*'-(3-chloro-4-hydroxybenzylidene)isonicotinohydrazide (C1)

Compound **C1** was synthesized analogously to compound **B**. Compound **B** (1 mmol) was dissolved in anhydrous ethanol, followed by the addition of 4-chloro-3-hydroxybenzaldehyde (1 mmol). The solution was heated to reflux at 80 °C for 8 h. Upon reaction completion, the product was obtained by recrystallization from ethanol.

248 mg, Yield, 90%. White solid powder. M.P. 232 °C–235 °C. ^1^H NMR (400 MHz, DMSO-*d*_6_) δ 12.06 (s, 1H), 8.80 (d, *J* = 5.8 Hz, 2H), 8.36 (s, 1H), 7.86 (d, *J* = 5.9 Hz, 2H), 7.73 (s, 1H), 7.55 (d, *J* = 8.4 Hz, 1H), 7.06 (d, *J* = 8.4 Hz, 1H). ^13^C NMR (101 MHz, DMSO-*d*_6_) δ 161.38, 155.19, 149.85, 148.04, 141.06, 128.64, 127.51, 126.34, 121.84, 120.42, 116.96. TOF-MS, m/z: [M + H]^+^, calcd. for C_13_H_11_ClN_3_O_2_^+^, 276.0540, found: 276.0544.

#### 4.1.3 *N*'-(2,5-dihydroxybenzylidene)isonicotinohydrazide (C2)

232 mg, Yield, 90%. White solid powder. M.P. 259 °C–261 °C. ^1^H NMR (400 MHz, DMSO-*d*_6_) δ 12.46 (s, 1H), 11.12 (s, 1H), 8.83 (d, *J* = 5.7 Hz, 2H), 8.69 (s, 1H), 7.92 (d, *J* = 5.8 Hz, 2H), 7.69 (d, *J* = 2.5 Hz, 1H), 7.32 (dd, *J* = 8.7, 2.5 Hz, 1H), 6.97 (d, *J* = 8.8 Hz, 1H). ^13^C NMR (101 MHz, DMSO-d6) δ 161.05, 150.28, 149.87, 149.69, 148.55, 140.57, 121.73, 119.34, 118.89, 117.11, 113.46. TOF-MS, m/z: [M + H]^+^, calcd. for C_13_H_12_N_3_O_3_^+^, 258.0878, found: 258.0853.

#### 4.1.4 *N*'-(2-hydroxy-5-methylbenzylidene)isonicotinohydrazide (C3)

217 mg, Yield, 85%. White solid powder. M.P. 189 °C–191 °C. ^1^H NMR (400 MHz, DMSO-*d*_6_) δ 12.26 (s, 1H), 10.83 (s, 1H), 8.80 (d, *J* = 5.9 Hz, 2H), 8.64 (s, 1H), 7.85 (d, *J* = 5.9 Hz, 2H), 7.40 (s, 1H), 7.12 (d, *J* = 8.3 Hz, 1H), 6.84 (d, *J* = 8.3 Hz, 1H), 2.25 (s, 3H). ^13^C NMR (101 MHz, DMSO-*d*_6_) δ 161.21, 155.30, 150.19, 149.04, 139.83, 132.28, 129.07, 127.85, 121.39, 118.13, 116.17, 19.76. TOF-MS, m/z: [M + H]^+^, calcd. for C_14_H_14_N_3_O_2_^+^, 256.1086, found: 256.1088.

#### 4.1.5 *N*'-(5-chloro-2-hydroxybenzylidene)isonicotinohydrazide (C4)

223 mg, Yield, 81%. White solid powder. M.P. 189 °C–191 °C. ^1^H NMR (400 MHz, DMSO-*d*_6_) δ 12.24 (s, 1H), 8.81 (d, *J* = 5.1 Hz, 2H), 8.64 (s, 1H), 7.89 (d, *J* = 5.1 Hz, 2H), 7.04 (s, 1H), 6.76 (s, 2H). ^13^C NMR (101 MHz, DMSO-*d*_6_) δ 161.27, 156.19, 149.56, 146.89, 140.76, 131.14, 127.37, 123.15, 122.05, 120.68, 118.31. TOF-MS, m/z: [M + H]^+^, calcd. for C_13_H_11_ClN_3_O_2_^+^, 276.0540, found: 276.0542.

#### 4.1.6 *N*'-(2-hydroxy-5-nitrobenzylidene)isonicotinohydrazide (**C5**)

252 mg, Yield, 88%. White solid powder. M.P. 242 °C–245 °C. ^1^H NMR (400 MHz, DMSO-*d*_6_) δ 12.44 (s, 1H), 12.19 (s, 1H), 8.90–8.67 (m, 3H), 8.61 (d, *J* = 2.7 Hz, 1H), 8.18 (dd, *J* = 9.1, 2.7 Hz, 1H), 7.87 (d, *J* = 5.5 Hz, 2H), 7.12 (d, *J* = 9.1 Hz, 1H). ^13^C NMR (101 MHz, DMSO-*d*_6_) δ 162.85, 161.76, 150.34, 145.36, 140.35, 140.18, 127.05, 123.69, 121.94, 120.21, 117.34. IR (KBr, cm-1): 3265 (NH), 1650 (CO), 1600 (NH). TOF-MS, m/z: [M + H]^+^, calcd. for C_13_H_11_N_4_O_4_^+^, 287.0780, found: 287.0782.

#### 4.1.7 *N*'-(pyridin-4-ylmethylene)isonicotinohydrazide (C6)

183 mg, Yield, 81%. White solid powder. M.P. 230 °C–232 °C. ^1^H NMR (400 MHz, DMSO-*d*_6_) δ 12.49 (s, 1H), 8.80 (d, *J* = 5.4 Hz, 2H), 8.69 (d, *J* = 5.4 Hz, 2H), 8.54 (s, 1H), 7.87 (d, *J* = 5.4 Hz, 2H), 7.73 (d, *J* = 5.4 Hz, 2H). ^13^C NMR (101 MHz, DMSO-*d*_6_) δ 162.48, 150.78, 150.04, 146.81, 142.49, 140.55, 122.12, 121.85. TOF-MS, m/z: [M + H]^+^, calcd. for C_12_H_11_N_4_O^+^, 227.0933, found: 227.0937.

#### 4.1.8 *N*'-[4-(methylsulfonyl)benzylidene]isonicotinohydrazide (C7)

270 mg, Yield, 89%. White solid powder. M.P. 209 °C–211 °C. ^1^H NMR (400 MHz, DMSO-*d*_6_) δ 12.41 (s, 1H), 8.84 (d, *J* = 5.6 Hz, 2H), 8.59 (s, 1H), 8.10–7.86 (m, 4H), 3.89 (s, 3H). ^13^C NMR (101 MHz, DMSO-*d*_6_) δ 162.07, 149.83, 147.75, 142.18, 141.69, 139.24, 128.39, 128.04, 122.62, 43.89. TOF-MS, m/z: [M + H]^+^, calcd. for C_14_H_14_N_3_O_3_S^+^, 304.0756, found: 304.0759.

#### 4.1.9 *N*'-[(1E,2E)-3-phenylallylidene]isonicotinohydrazide (C8)

213 mg, Yield, 85%. White solid powder. M.P. 164 °C–166 °C. ^1^H NMR (400 MHz, DMSO-*d*_6_) δ 12.07 (s, 1H), 8.82 (d, *J* = 5.6 Hz, 2H), 8.29 (d, *J* = 6.7 Hz, 1H), 7.90 (d, *J* = 4.8 Hz, 2H), 7.64 (d, *J* = 7.4 Hz, 2H), 7.53–7.29 (m, 3H), 7.10 (d, *J* = 6.6 Hz, 2H). ^13^C NMR (101 MHz, DMSO-*d*_6_) δ 161.20, 151.22, 149.45, 141.33, 140.03, 135.80, 129.05, 128.87, 127.24, 125.39, 122.04, 39.52. TOF-MS, m/z: [M + H]^+^, calcd. for C_15_H_14_N_3_O^+^, 252.1137, found: 252.1141.

#### 4.1.10 *N*'-(4-methylbenzylidene)isonicotinohydrazide (C9)

196 mg, Yield, 82%. White solid powder. M.P. 186 °C–188 °C. ^1^H NMR (400 MHz, DMSO-*d*_6_) δ 11.99 (s, 1H), 8.78 (d, *J* = 5.4 Hz, 2H), 8.43 (s, 1H), 7.82 (d, *J* = 5.4 Hz, 2H), 7.65 (d, *J* = 7.9 Hz, 2H), 7.29 (d, *J* = 7.9 Hz, 2H), 2.35 (s, 3H). ^13^C NMR (101 MHz, DMSO-*d*_6_) δ 161.58, 150.33, 149.13, 140.56, 140.29, 131.36, 129.50, 127.29, 121.56, 21.06. TOF-MS, m/z: [M + H]^+^, calcd. for C_14_H_14_N_3_O^+^, 240.1137, found: 240.1141.

#### 4.1.11 *N*′-benzylideneisonicotinohydrazide (C10)

203 mg, Yield, 90%. White solid powder. M.P. 191°C–193°C. ^1^H NMR (400 MHz, DMSO-*d*_6_) δ 12.29 (s, 1H), 8.85 (d, *J* = 5.4 Hz, 2H), 8.55 (s, 1H), 7.98 (d, *J* = 5.4 Hz, 2H), 7.75 (d, *J* = 7.0 Hz, 2H), 7.47 (s, 3H). ^13^C NMR (101 MHz, DMSO-*d*_6_) δ 160.98, 149.24, 148.61, 141.80, 133.83, 130.26, 128.72, 127.12, 122.23, 39.52. TOF-MS, m/z: [M + H]^+^, calcd. for C_13_H_12_N_3_O^+^, 226.0980, found: 226.0983.

#### 4.1.12 *N*'-(4-nitrobenzylidene)isonicotinohydrazide (C11)

243 mg, Yield, 90%. White solid powder. M.P. 237 °C–239 °C. ^1^H NMR (400 MHz, DMSO-*d*_6_) δ 12.61 (s, 1H), 8.87 (d, *J* = 6.1 Hz, 2H), 8.66 (s, 1H), 8.31 (d, *J* = 8.7 Hz, 2H), 8.01 (d, *J* = 9.0 Hz, 4H). ^13^C NMR (101 MHz, DMSO-*d*_6_) δ 161.70, 149.11, 148.30, 147.04, 141.86, 140.46, 128.47, 124.33, 122.64. TOF-MS, m/z: [M + H]^+^, calcd. for C_13_H_11_N_4_O_3_^+^, 271.0831, found: 271.0835.

#### 4.1.13 4-[(2-isonicotinoylhydrazineylidene)methyl]benzoic acid (C12)

237 mg, Yield, 88%. White solid powder. M.P. 313 °C–315 °C. ^1^H NMR (400 MHz, DMSO-d6) δ 12.45 (s, 1H), 8.86 (d, *J* = 6.0 Hz, 2H), 8.61 (s, 1H), 8.01 (dd, *J* = 12.6, 7.2 Hz, 4H), 7.87 (d, *J* = 8.3 Hz, 2H). ^13^C NMR (101 MHz, DMSO-*d*_6_) δ 167.34, 161.70, 148.99, 148.68, 146.87, 142.64, 138.45, 132.52, 130.31, 127.81, 123.05. TOF-MS, m/z: [M + H]^+^, calcd. for C_14_H_12_N_3_O_3_^+^, 270.0878, found: 270.0881.

#### 4.1.14 *N*'-(4-hydroxybenzylidene)isonicotinohydrazide (C13)

210 mg, Yield, 87%. White solid powder. M.P. 244 °C–246 °C. ^1^H NMR (400 MHz, DMSO-*d*_6_) δ 12.04 (s, 1H), 8.81 (d, *J* = 6.0 Hz, 2H), 8.42 (s, 1H), 7.93 (d, *J* = 6.1 Hz, 2H), 7.58 (d, *J* = 8.6 Hz, 2H), 6.86 (d, *J* = 8.6 Hz, 2H). ^13^C NMR (101 MHz, DMSO-*d*_6_) δ 160.80, 159.65, 149.50, 149.02, 141.65, 128.98, 124.79, 121.96, 115.66, 39.52. TOF-MS, m/z: [M + H]^+^, calcd. for C_13_H_12_N_3_O_2_^+^, 242.0929, found: 242.0933.

#### 4.1.15 *N*'-(quinolin-4-ylmethylene)isonicotinohydrazide (C14)

248 mg, Yield, 90%. White solid powder. M.P. 204 °C–206 °C. ^1^H NMR (400 MHz, DMSO-*d*_6_) δ 12.81 (s, 1H), 9.32 (s, 1H), 9.05 (d, *J* = 4.6 Hz, 1H), 8.84 (d, *J* = 5.7 Hz, 2H), 8.74 (d, *J* = 8.4 Hz, 1H), 8.15 (d, *J* = 8.3 Hz, 1H), 8.03–7.85 (m, 5H). ^13^C NMR (101 MHz, DMSO-*d*_6_) δ 162.40, 150.48, 149.63, 146.73, 146.28, 141.12, 139.91, 131.42, 128.94, 128.58, 125.06, 122.62, 120.50. TOF-MS, m/z: [M + H]^+^, calcd. for C_16_H_13_N_4_O^+^, 277.1089, found: 277.1094.

#### 4.1.16 *N*'-(furan-2-ylmethylene)isonicotinohydrazide (C15)

176 mg, Yield, 82%. White solid powder. M.P. 257 °C–259 °C. ^1^H NMR (400 MHz, DMSO-*d*_6_) δ 12.51 (s, 1H), 8.91 (d, *J* = 5.2 Hz, 3H), 8.52 (s, 1H), 8.13 (d, *J* = 6.3 Hz, 3H), 7.88 (s, 1H), 6.99 (d, *J* = 3.4 Hz, 2H), 6.65 (dd, *J* = 3.3, 1.7 Hz, 2H). ^13^C NMR (101 MHz, DMSO-*d*_6_) δ 159.96, 148.67, 146.50, 145.29, 138.86, 122.94, 114.25, 111.95. TOF-MS, m/z: [M + H]^+^, calcd. for C_11_H_10_N_3_O_2_^+^, 216.0773, found: 216.0776.

#### 4.1.17 *N*'-(thiophen-2-ylmethylene)isonicotinohydrazide (C16)

194 mg, Yield, 84%. White solid powder. M.P. 217 °C–219 °C. ^1^H NMR (400 MHz, DMSO-*d*_6_) δ 12.04 (s, 1H), 8.78 (d, *J* = 5.9 Hz, 2H), 8.69 (s, 1H), 7.81 (d, *J* = 5.9 Hz, 2H), 7.69 (d, *J* = 5.0 Hz, 1H), 7.51 (d, *J* = 3.4 Hz, 1H), 7.18–7.11 (m, 1H). ^13^C NMR (101 MHz, DMSO-*d*_6_) δ 161.93, 150.76, 144.56, 140.88, 139.20, 132.00, 129.88, 128.39, 121.94. TOF-MS, m/z: [M + H]^+^, calcd. for C_11_H_9_N_3_OS^+^, 232.0544, found: 232.0547.

### 4.2 Determination of minimum inhibitory concentration

For detailed procedures, refer to the Supplementary Material (Stratev and Fasulkova, 2024).

### 4.3 Time-killing kinetics

Time-Kill Kinetics The time-kill kinetics of compound **C5** against *S. aureus* ATCC 29213 were assessed by the viable plate count method. Detailed procedures followed those described in previous reports.

### 4.4 Drug resistance study

The drug resistance study of compound **C5** was performed by following the protocol of previous study. The initial MIC values of **C5** against *S*. *aureus* ATCC 29213 was determined according to method described above. The process was repeated continuously for 28 days.

### 4.5 Hemolysis assay

For detailed procedures, refer to the Supplementary Material (Hong et al., 2025; Sæbø et al., 2023).

### 4.6 Cytotoxicity assay

Cytotoxicity was assessed using the Cell Counting Kit-8 (CCK-8; Beyotime, Shanghai, China), following established methods with minor modifications. Cell viability was calculated as follows: Cell viability (%) = [(OD_450,sample_ - OD_450,blank_)/(OD_450,control_ - OD_450,blank_)] × 100% (Mine et al., 2020).

### 4.7 Biofilm formation assay

For detailed procedures, refer to the Supplementary Material (Li et al., 2024).

### 4.8 The anti-inflammatory activity of the compounds

For detailed procedures, refer to the Supplementary Material (Moudgil and Venkatesha, 2022).

### 4.9 Membrane depolarization study

For detailed procedures, refer to the Supplementary Material (Hong et al., 2025).

### 4.10 DNA and protein leakage

For detailed procedures, refer to the Supplementary Material (Hong et al., 2025).

### 4.11 ROS detection assay

For detailed procedures, refer to the Supplementary Material (Zhang et al., 2022).

### 4.12 Intracellular ATP measurement

For detailed procedures, refer to the Supplementary Material (Morciano et al., 2020).

### 4.13 Statistical analysis

The above experimental data is the average ±SEM (Standard Error of Mean) independent experiment of at least three data points. SPSS 21.0 software was used to analyze the data, and one-way analysis of variance (ANOVA) was used to process the statistical differences between the two groups.

## Data Availability

The original contributions presented in the study are included in the article/Supplementary Material, further inquiries can be directed to the corresponding author/s.

## References

[B1] Asenjo A. Oteo-Iglesias J. Alós J. I. (2021). What's new in mechanisms of antibiotic resistance in bacteria of clinical origin? Enferm. Infecc. Microbiol. Clin. 39, 291–299. 10.1016/j.eimce.2020.02.017 34088451

[B2] Backes G. L. Jursic B. S. Neumann D. M. (2015). Potent antimicrobial agents against azole-resistant fungi based on pyridinohydrazide and hydrazomethylpyridine structural motifs. Bioorg Med. Chem. 23, 3397–3407. 10.1016/j.bmc.2015.04.040 25943854

[B3] Barua M. Bandyopadhyay S. Wasai A. Ghosh M. Roy I. Ghosh P. (2024). A trinuclear Zn (II) schiff base dicyanamide complex attenuates bacterial biofilm formation by ROS generation and membrane damage and exhibits anticancer activity. Microb. Pathog. 188, 106548. 10.1016/j.micpath.2024.106548 38262493

[B4] Bhowmik P. Modi B. Roy P. Chowdhury A. (2023). Strategies to combat Gram-negative bacterial resistance to conventional antibacterial drugs: a review. Osong Public Health Res. Perspect. 14, 333–346. 10.24171/j.phrp.2022.0323 37920891 PMC10626324

[B5] Caldés C. Vilanova B. Adrover M. Donoso J. Muñoz F. (2013). The hydrophobic substituent in aminophospholipids affects the formation kinetics of their schiff bases. Bioorg Med. Chem. Lett. 23, 2202–2206. 10.1016/j.bmcl.2013.01.100 23462644

[B6] Cheung G. Y. C. Bae J. S. Otto M. (2021). Pathogenicity and virulence of *Staphylococcus aureus* . Virulence 12, 547–569. 10.1080/21505594.2021.1878688 33522395 PMC7872022

[B7] Dörner P. J. Anandakumar H. Röwekamp I. Fiocca-Vernengo F. Millet B. Krzanowski M. (2024). Clinically used broad-spectrum antibiotics compromise inflammatory monocyte-dependent antibacterial defense in the lung. Nat. Commun. 15, 2788. 10.1038/s41467-024-47149-z 38555356 PMC10981692

[B8] Fontana R. Marconi P. C. R. Caputo A. Gavalyan V. B. (2022). Novel chitosan-based schiff base compounds: Chemical characterization and antimicrobial activity. Molecules 27, 2740. 10.3390/molecules27092740 35566088 PMC9102824

[B9] García-Castro M. Sarabia F. Díaz-Morilla A. López-Romero J. M. (2023). Approved antibacterial drugs in the last 10 years: from the bench to the clinic. Explor. Drug Sci. 3, 180–209. 10.37349/eds.2023.00013

[B10] Han J. Klika K. D. Makarem A. (2025). Byproducts in the synthesis of [68Ga]Ga-PSMA-11. Nat. Protoc. 18. 10.1038/s41596-025-01164-6 40251421

[B11] Hong S. Lu H. Tian D. Chang Y. Lu Q. Gao F. (2025). Discovery of triazole derivatives for biofilm disruption, anti-inflammation and metal ion chelation. Front. Chem. 26, 1545259. 10.3389/fchem.2025.1545259 40078565 PMC11897050

[B12] Hu C. W. Chang Y. C. Liu C. H. Yu Y. A. Mou K. Y. (2022). Development of a TNF-α-mediated trojan horse for bacteria-based cancer therapy. Mol. Ther. 30, 2522–2536. 10.1016/j.ymthe.2022.04.008 35440418 PMC9263318

[B13] Islam N. Reid D. (2024). Inhaled antibiotics: a promising drug delivery strategies for efficient treatment of lower respiratory tract infections (LRTIs) associated with antibiotic resistant biofilm-dwelling and intracellular bacterial pathogens. Respir. Med. 227, 107661. 10.1016/j.rmed.2024.107661 38729529

[B14] Ji Y. Dai F. Zhou B. (2018). Designing salicylaldehyde isonicotinoyl hydrazones as Cu(II) ionophores with tunable chelation and release of copper for hitting redox achilles heel of cancer cells. Free Radic. Biol. Med. 129, 215–226. 10.1016/j.freeradbiomed.2018.09.017 30240704

[B15] Kaur M. Kumar S. Yusuf M. Lee J. Malik A. K. Ahmadi Y. (2023). Schiff base-functionalized metal-organic frameworks as an efficient adsorbent for the decontamination of heavy metal ions in water. Environ. Res. 236, 116811. 10.1016/j.envres.2023.116811 37541413

[B16] Klika K. D. Alsalim R. Eftekhari M. Makarem A. (2022). Synthesis of a polyaminocarboxylate-based aluminum complex and its structural studies using 1H{^13^C}-HMBC NMR and a Karplus-type function. Dalton Trans. 51, 12436–12441. 10.1039/d2dt01702d 35943556

[B17] Kopka K. Makarem A. Sarvestani M. K. Klika K. D. (2019). A multifunctional HBED-type chelator with dual conjugation capabilities for radiopharmaceutical development. Synlett 30, 1795–1798. 10.1055/s-0039-1690194

[B18] Lakhundi S. Zhang K. (2018). Methicillin-Resistant staphylococcus aureus: Molecular Characterization, Evolution, and epidemiology. Clin. Microbiol. Rev. 31, e00020-18–18. 10.1128/CMR.00020-18 30209034 PMC6148192

[B19] Li M. Cruz C. D. Ilina P. Tammela P. (2024). High-throughput combination assay for studying biofilm formation of uropathogenic Escherichia coli. Arch. Microbiol. 206, 344. 10.1007/s00203-024-04029-w 38967798 PMC11226472

[B20] Mine Y. Suga M. Mimura S. Minoda M. Murayama T. Nikawa H. (2020). Cytotoxicity assay using a human pluripotent stem cell-derived cranial neural crest cell model. Vitro Cell Dev. Biol. Anim. 56, 505–510. 10.1007/s11626-020-00491-0 32812205

[B21] Mohr K. I. (2016). History of antibiotics research. Curr. Top. Microbiol. Immunol. 398, 237–272. 10.1007/82_2016_499 27738915

[B22] Morciano G. Imamura H. Patergnani S. Pedriali G. Giorgi C. Pinton P. (2020). Measurement of ATP concentrations in mitochondria of living cells using luminescence and fluorescence approaches. Methods Cell Biol. 155, 199–219. 10.1016/bs.mcb.2019.10.007 32183959

[B23] Moudgil K. D. Venkatesha S. H. (2022). The anti-inflammatory and immunomodulatory activities of natural products to control autoimmune inflammation. Int. J. Mol. Sci. 24, 95. 10.3390/ijms24010095 36613560 PMC9820125

[B24] Poulton N. C. Rock J. M. (2022). Unraveling the mechanisms of intrinsic drug resistance in *Mycobacterium tuberculosis* . Front. Cell Infect. Microbiol. 17, 997283. 10.3389/fcimb.2022.997283 36325467 PMC9618640

[B25] Presenjit P. Chaturvedi S. Singh A. Gautam D. Singh K. Mishra A. K. (2024). An insight into the effect of schiff base and their d and f block metal complexes on various cancer cell lines as anticancer agents: a review. Anticancer Agents Med. Chem. 24, 488–503. 10.2174/0118715206280314231201111358 38279753

[B26] Rana M. S. Rayhan N. M. A. Emon M. S. H. Islam M. T. Rathry K. Hasan M. M. (2024). Antioxidant activity of schiff base ligands using the DPPH scavenging assay: an updated review. RSC Adv. 14, 33094–33123. 10.1039/d4ra04375h 39434996 PMC11492428

[B27] Ridahunlang N. Bisht R. Rishanlang N. (2023). Isoniazid derivatives as anti-tubercular agents: from structural design to clinical investigations. Infect. Disord. Drug Targets 223, e041022209552. 10.2174/1871526522666221004152324 36200205

[B28] Rouzi K. Altay A. Bouatia M. Yeniçeri E. Islam M. S. Oulmidi A. (2024). Novel isoniazid-hydrazone derivatives induce cell growth inhibition, cell cycle arrest and apoptosis *via* mitochondria-dependent caspase activation and PI3K/AKT inhibition. Bioorg Chem. 150, 107563. 10.1016/j.bioorg.2024.107563 38885547

[B29] Sæbø I. P. Bjørås M. Franzyk H. Helgesen E. Booth J. A. (2023). Optimization of the hemolysis assay for the assessment of cytotoxicity. Int. J. Mol. Sci. 24, 2914. 10.3390/ijms24032914 36769243 PMC9917735

[B30] Sirinirund B. Siqueira R. Li J. Mendonça G. Zalucha J. Wang H. L. (2023). Effects of crown contour on artificial biofilm removal efficacy with interdental cleaning aids: an *in vitro* study. Clin. Oral Implants Res. 34, 783–792. 10.1111/clr.14105 37269176

[B31] Sodré-Alves B. M. C. Toledo M. M. Zimmermann I. R. Araújo W. N. Tavares N. U. L. (2024). Isoniazid use, effectiveness, and safety for treatment of latent tuberculosis infection: a systematic review. Rev. Soc. Bras. Med. Trop. 25, e004022024. 10.1590/0037-8682-0504-2023 38536998 PMC10962359

[B32] Stratev D. Fasulkova R. (2024). Minimum inhibitory concentration of doxycycline for Vibrio parahaemolyticus. Indian J. Med. Microbiol. 48, 100532. 10.1016/j.ijmmb.2024.100532 38237736

[B33] Tu C. Lu H. Zhou T. Zhang W. Deng L. Cao W. (2022). Promoting the healing of infected diabetic wound by an anti-bacterial andnano-enzyme-containing hydrogel with inflammation-suppressing, ROS-scavenging, oxygen and nitric oxide-generating properties. Biomaterials 286, 121597. 10.1016/j.biomaterials.2022.121597 35688112

[B34] Udhayakumari D. Inbaraj V. (2020). A review on schiff base fluorescent chemosensors for cell imaging applications. J. Fluoresc. 30, 1203–1223. 10.1007/s10895-020-02570-7 32737660

[B35] Vyas N. Grewal M. Kuehne S. A. Sammons R. L. Walmsley A. D. (2020). High speed imaging of biofilm removal from a dental implant model using ultrasonic cavitation. Dent. Mater 36, 733–743. 10.1016/j.dental.2020.03.003 32299665

[B36] Xiao X. Huan Q. Huang Y. Liu Z. Liu Y. Li R. (2024). Gramine sensitizes *Klebsiella pneumoniae* to tigecycline killing. Phytomedicine 126, 155421. 10.1016/j.phymed.2024.155421 38430819

[B37] Zhang X. Zheng X. Ying X. Xie W. Yin Y. Wang X. (2022). CEBPG suppresses ferroptosis through transcriptional control of SLC7A11 in ovarian cancer. J. Transl. Med. 21, 334. 10.1186/s12967-023-04136-0 37210575 PMC10199564

