# Correction: Membrane-targeting antibacterial isoniazid schiff base against *S. aureus* and biofilms

**DOI:** 10.3389/fchem.2025.1706525

**Published:** 2025-10-09

**Authors:** Yaguang Liu, Lianzhi Hu, Binbin Liu, Zheng Qu

**Affiliations:** Pharmacy Department, The Second Hospital of Qinhuangdao, Qinhuangdao, China

**Keywords:** isoniazid, schiff base, antibacterial activity, anti biofilm, anti inflammatory

There was a mistake in Figure 7 as published. Panels A and B inadvertently contained duplicate images. The corrected Figure 7 appears below.

**FIGURE 7:**
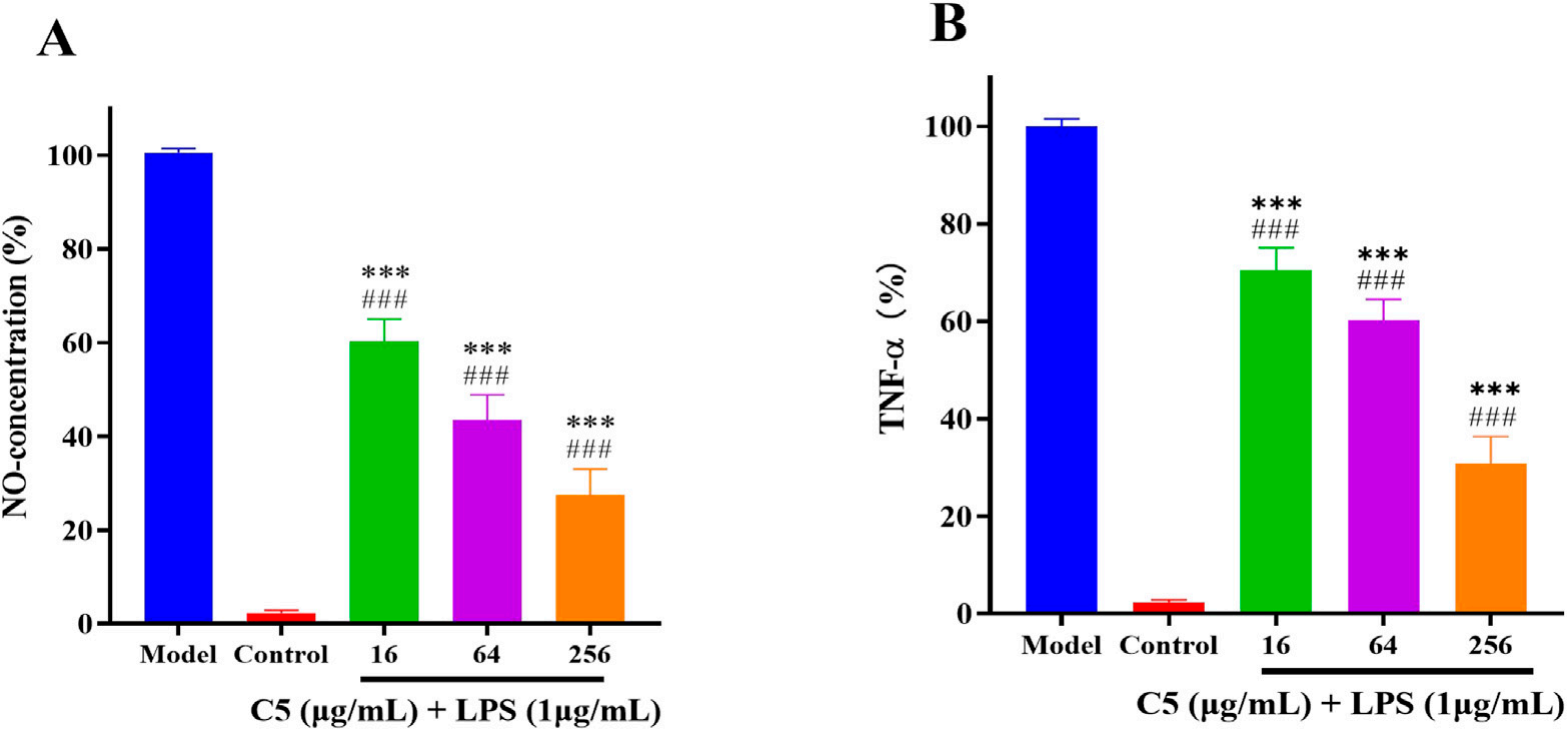
Anti-inflammatory activity of the C5 compounds in RAW 264.7 macrophage cells was evaluated in the LPS-enhanced leukocyte migration assay. **(A)** C5 affects the level of NO. **(B)** C5 affects the level of TNF-α. Compared with the LPS model group, ^*^p < 0.05, ^**^p < 0.01, ^***^p < 0.001; ^###^p < 0.001 vs. control group. Data are presented as means ± SEM from three independent experiments.

The original article has been updated.

