# Supplementary material for: Membrane-targeting antibacterial isoniazid schiff base against S. aureus and biofilms
Source: Front Chem. 2025 Sep 9;13:1654358. doi: 10.3389/fchem.2025.1654358 (PMC12454367; doi:10.3389/fchem.2025.1654358)
Supplement: Supplementary file 1 [file DataSheet1.doc]

**Membrane-Targeting Antibacterial Isoniazid Schiff Base against**

***S. aureus* and Biofilms**

Yaguang Liu^1*^, Lianzhi Hu[[1]](#footnote-2), Binbin Liu^1^, Zheng Qu^1^

*^1^The Second Hospital of Qinhuangdao, Pharmacy Department, Qinhuangdao, China, 066000*

**Table of Contents**

**Determination of Minimum Inhibitory Concentration**···································1

**Hemolysis assay**·····························································································1

**Biofilm Formation Assay**···············································································1

**The anti-inflammatory activity of the compounds**··········································2

**Membrane depolarization study**····································································2

**DNA and protein leakage**··············································································2

**ROS Detection Assay**·················································································3

**Intracellular ATP Measurement**·······························································3

**Spectral data**······························································································4-21

**Determination of Minimum Inhibitory Concentration**

The minimum inhibitory concentrations (MICs) of all test compounds were determined using the microdilution method according to Clinical and Laboratory Standards Institute (CLSI) guidelines. Bacterial strains were grown in Mueller-Hinton broth (MHB) on a shaker at 37°C for ~5 h, adjusted to ~10⁵ CFU/mL, and dispensed into 96-well microtiter plates. Compounds were added at serial concentrations (0.05–256 μg/mL). After incubation at 37°C for 18 h, MICs were defined as the lowest concentration showing no visible growth. All tests were performed in triplicate.

**Hemolysis assay**

The hemolysis assay was performed as described previously. Rabbit erythrocytes (4% v/v suspension in PBS) were mixed with equal volumes (100 µL each) of C5 in PBS, resulting in final C5 concentrations of 2-256 µg/mL. Controls included 1% Triton X-100 (positive control) and PBS (negative control). After incubation at 37°C for 1 h, samples were centrifuged (1000 × g, 5 min). The absorbance of the supernatant was then measured at 490 nm. Hemolysis (%) was calculated using the formula: Hemolysis (%) = [(Sample − PBS) / (Triton − PBS)] × 100. All experiments were performed in triplicate.

**Biofilm Formation Assay**

Biofilm Inhibition Assay: *S. aureus* ATCC 29213 was diluted 100-fold in fresh TSB containing 1% (w/v) glucose. Diluted bacteria and compounds were added to wells containing 200 µL TSB + 1% glucose. **C5** was added directly (final concentrations: 2–256 µg/mL). Controls received an equivalent volume of DMSO. Plates were incubated at 37°C for 24 h to allow biofilm formation in the presence of compounds. After incubation, biofilms were rinsed (3× PBS), air-dried, stained with 0.1% crystal violet (15 min), rinsed again (3× PBS), and the bound dye was dissolved in 95% ethanol. Absorbance was measured at 595 nm. Biofilm inhibition (%) was calculated as: [(OD_595_ control - OD_595_ sample) / OD_595_ control] × 100.

Biofilm Eradication Assay: Biofilms were first established by incubating bacterial suspensions in TSB + 1% glucose at 37°C for 24 h. Then, sub-inhibitory concentrations of **C5** (2–256 µg/mL) were added directly to the pre-formed biofilms. Subsequent steps (rinsing, staining, dissolution, measurement, calculation) were identical to the inhibition assay. All experiments were performed in triplicate.

**The anti-inflammatory activity of the compounds**

The effect of compounds on nitric oxide (NO) production was assessed in LPS-stimulated RAW 264.7 cells. Cells were treated for 24 h with either LPS alone (control) or LPS plus test compounds. Nitrite accumulation in cell supernatants, quantified using the Griess reaction in a microplate assay, served as an indicator of NO production. NO levels were expressed as a percentage relative to the LPS control: (NO_sample_ / NO_control_) × 100.

**Membrane depolarization study**

Membrane Potential Assay (DiSC35): A *S. aureus* ATCC 29213 colony was inoculated into LB broth and shaken at 37°C for 6 h. Cells were pelleted (5 min), washed 3 × PBS, and resuspended in PBS to 1×10⁸ CFU/mL. Suspension (150 μL) was added to a black 96-well plate, followed by DiSC35 (10 μM, 40 μL) in the dark. After 30 min incubation (37°C, dark), fluorescence intensity (λ _622 nm_ / λ _670 nm_) was measured every 5 min for 40 min. **C5** solution (10 μL) was then added, and measurements continued for another 40 min.

Membrane Integrity Assay (SYTOX Green): *S. aureus* suspension (10⁸ CFU/mL, 150 μL) was added to a black 96-well plate, followed by SYTOX Green (3 μM, 40 μL) in the dark. After 30 min incubation (37°C, dark), fluorescence intensity (λ_500 nm_ / λ_530 nm_) was monitored every 5 min for 40 min. **C5** dilutions (10 μL) were added to achieve final concentrations of 64 or 256 μg/mL, and measurements continued for 40 min.

**DNA and protein leakage**

*S. aureus* ATCC 29213 suspensions (2 × 10^6^ CFU/mL) were treated with **C5** solution at final concentrations corresponding to 8 × MIC, 4 × MIC, 2 × MIC, 1 × MIC, 1/2 × MIC, and 1/4 × MIC. The mixtures were incubated at 37°C for 4 h, centrifuged, and the supernatant was collected for analysis. DNA concentration was quantified using a microspectrophotometer, while protein concentration was determined using a BCA Protein Assay Kit.

**ROS Detection Assay**

The impact of **C5** on bacterial ROS accumulation was evaluated using the ROS-sensitive fluorescent probe 2',7'-dichlorofluorescein diacetate (DCFH-DA). Bacterial strains stored at -80°C were revived and cultured in fresh Mueller-Hinton broth (5 mL) at 37°C with shaking (200 rpm) to mid-log phase. Cells were pelleted by centrifugation (4500 × g, room temperature), washed with sterile PBS, and resuspended to OD_600_ = 0.5. The suspension was incubated with 10 μM DCFH-DA at 37°C for 20 min. After probe labeling, cells were washed and resuspended. Aliquots (190 μL) of labeled bacteria were transferred to a 96-well plate, followed by addition of 10 μL test compounds. Following 30 min incubation at 37°C, fluorescence intensity was measured (λ _488 nm_ / λ _525 nm_).

**Intracellular ATP Measurement**

The effect of **C5** on bacterial intracellular ATP levels was assessed using an Enhanced ATP Assay Kit. Test strains were cultured in fresh Mueller-Hinton broth (5 mL) at 37°C with shaking (200 rpm) to mid-log phase. Cells were pelleted by centrifugation (4500 × g, room temperature), washed with sterile PBS, and resuspended to OD_600_ = 0.5. Test compounds were added and incubated at 37°C for 30 min. After centrifugation (10,000 × g, 5 min, 4°C), pellets were lysed and centrifuged. The resulting supernatant was used for ATP quantification according to the manufacturer's protocol using a multi-mode microplate reader.


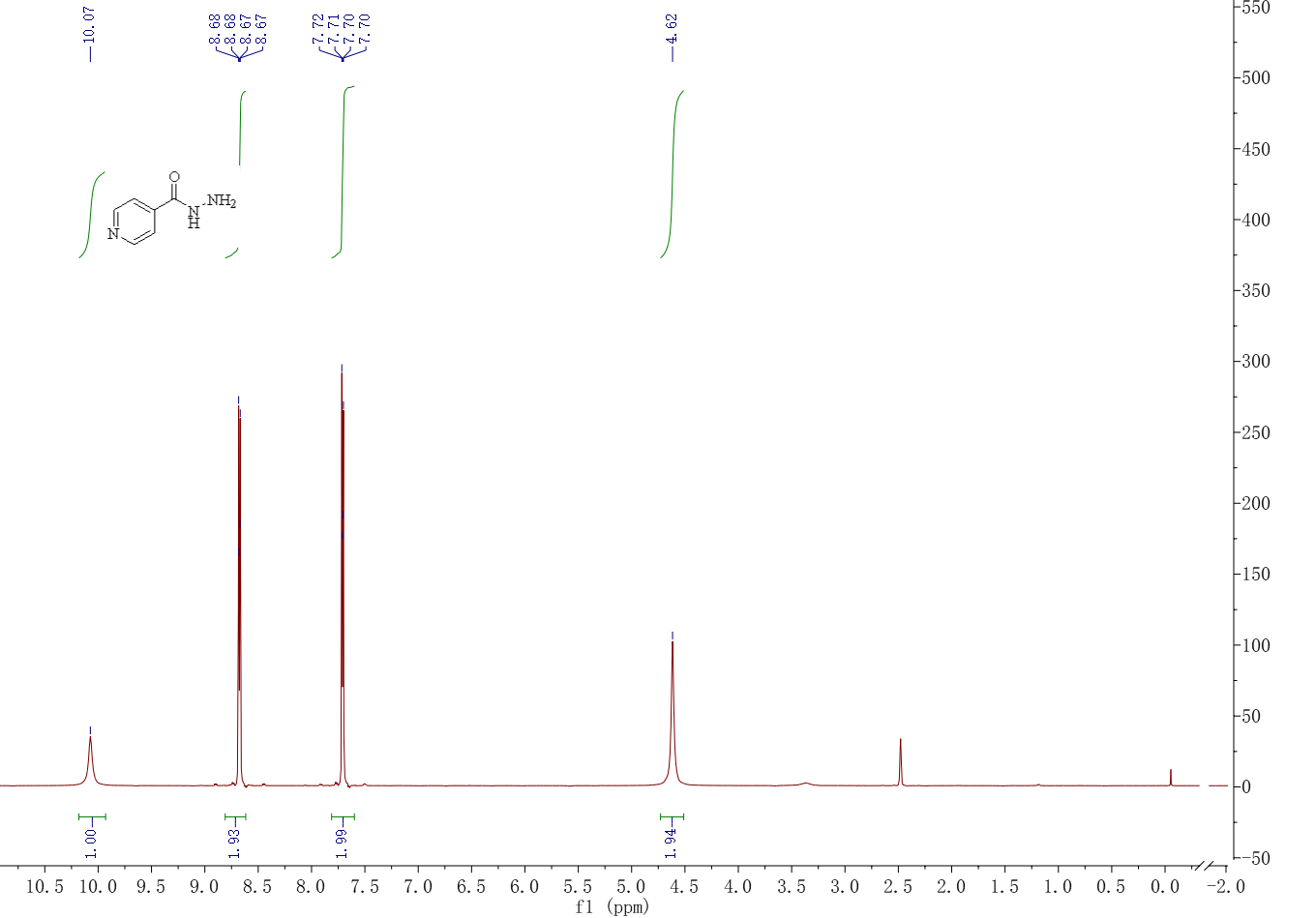


Fig. *^1^H NMR of* **B** (400 MHz, DMSO)


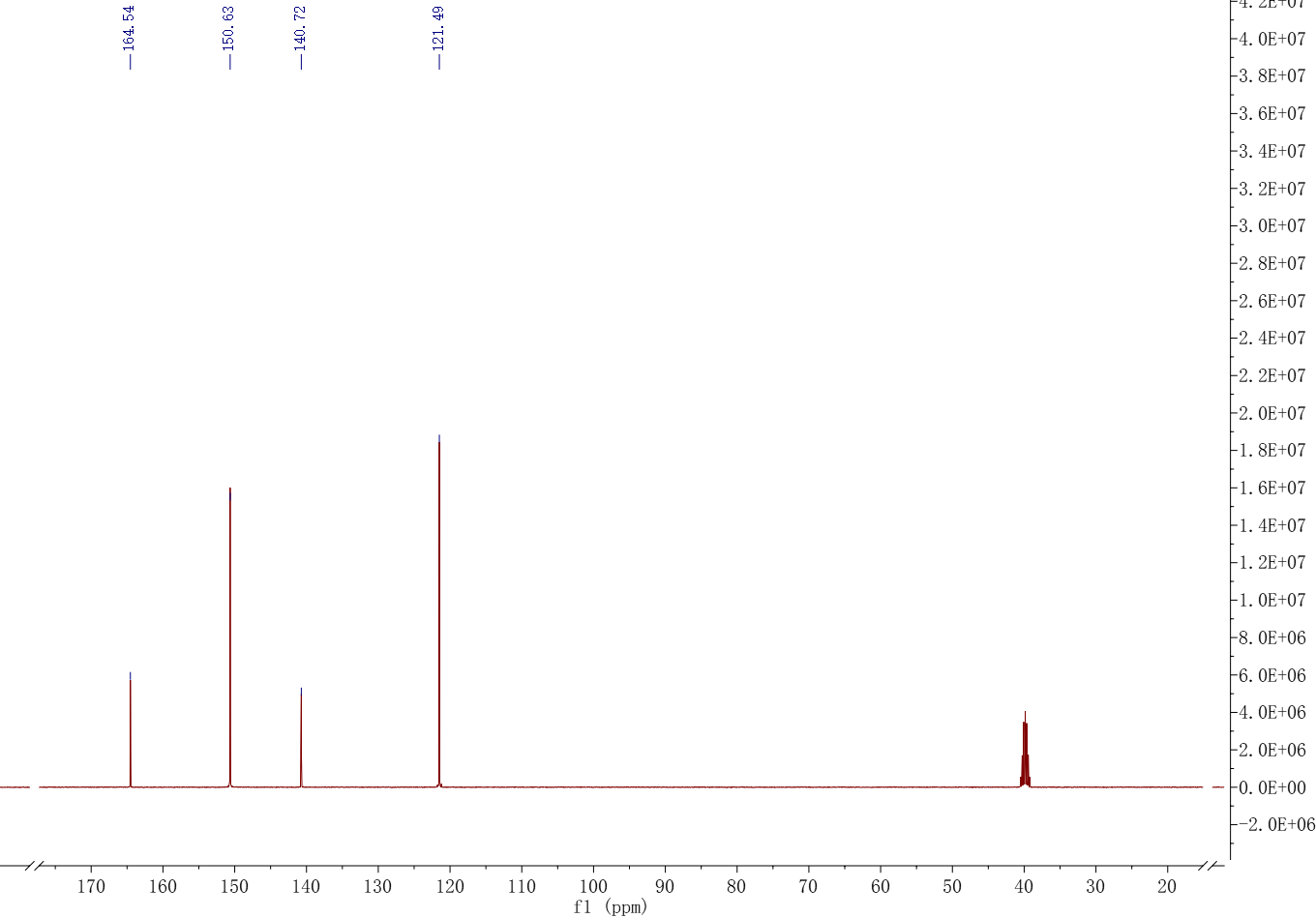


Fig. *^13^C NMR of* **B** (100 MHz, DMSO)


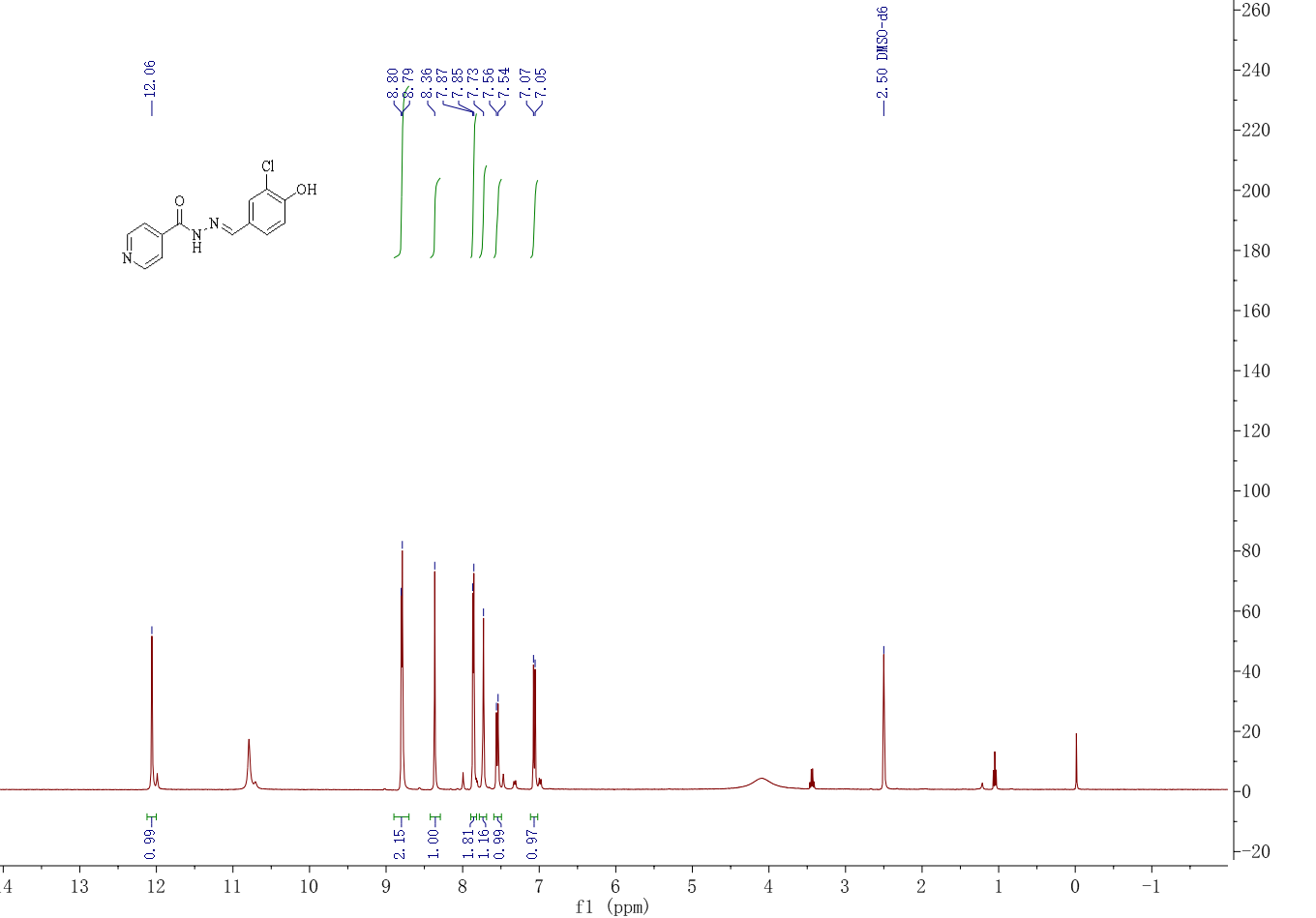


Fig. *^1^H NMR of* **C1** (400 MHz, DMSO)


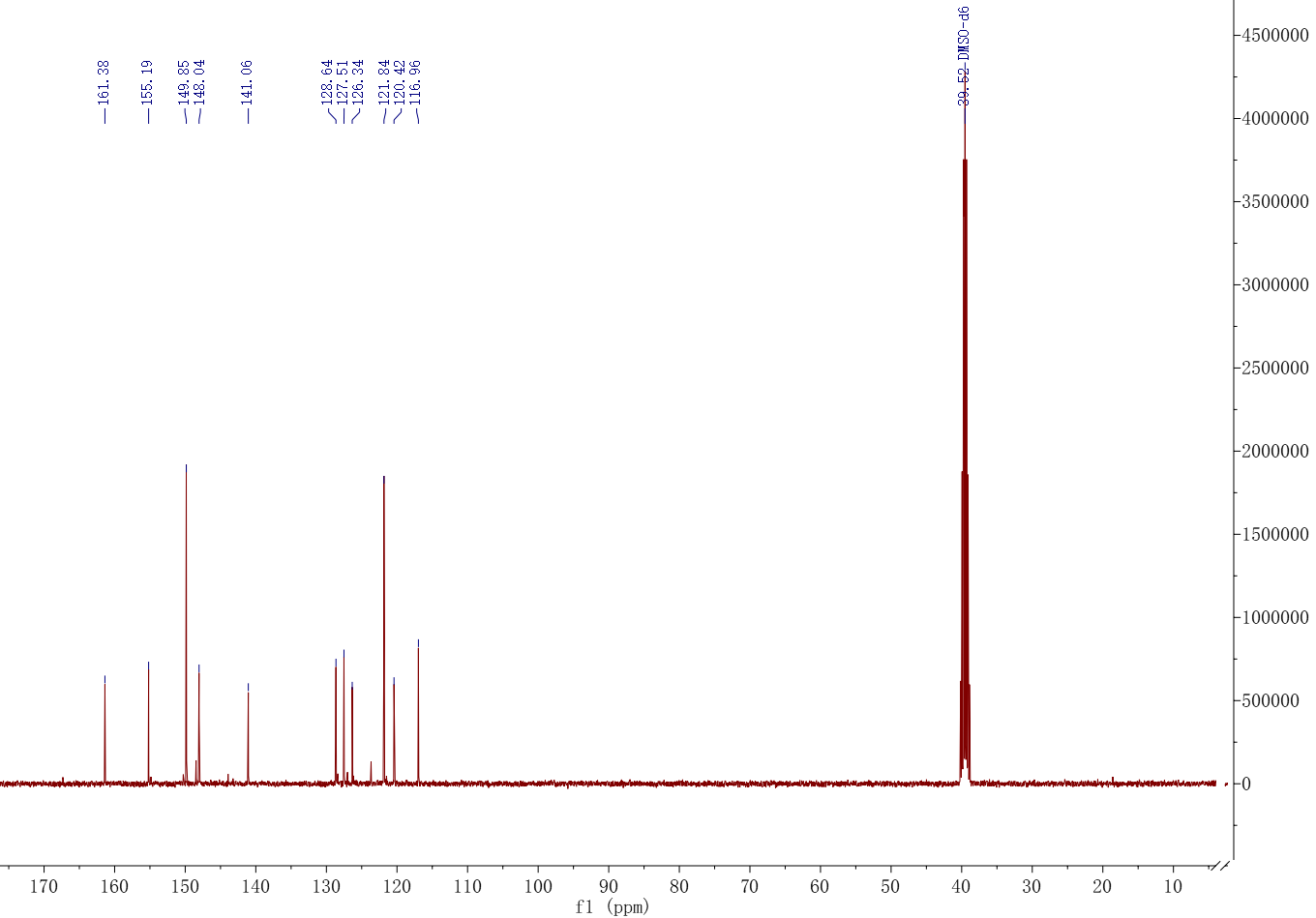


Fig. *^13^C NMR of* **C1** (100 MHz, DMSO)


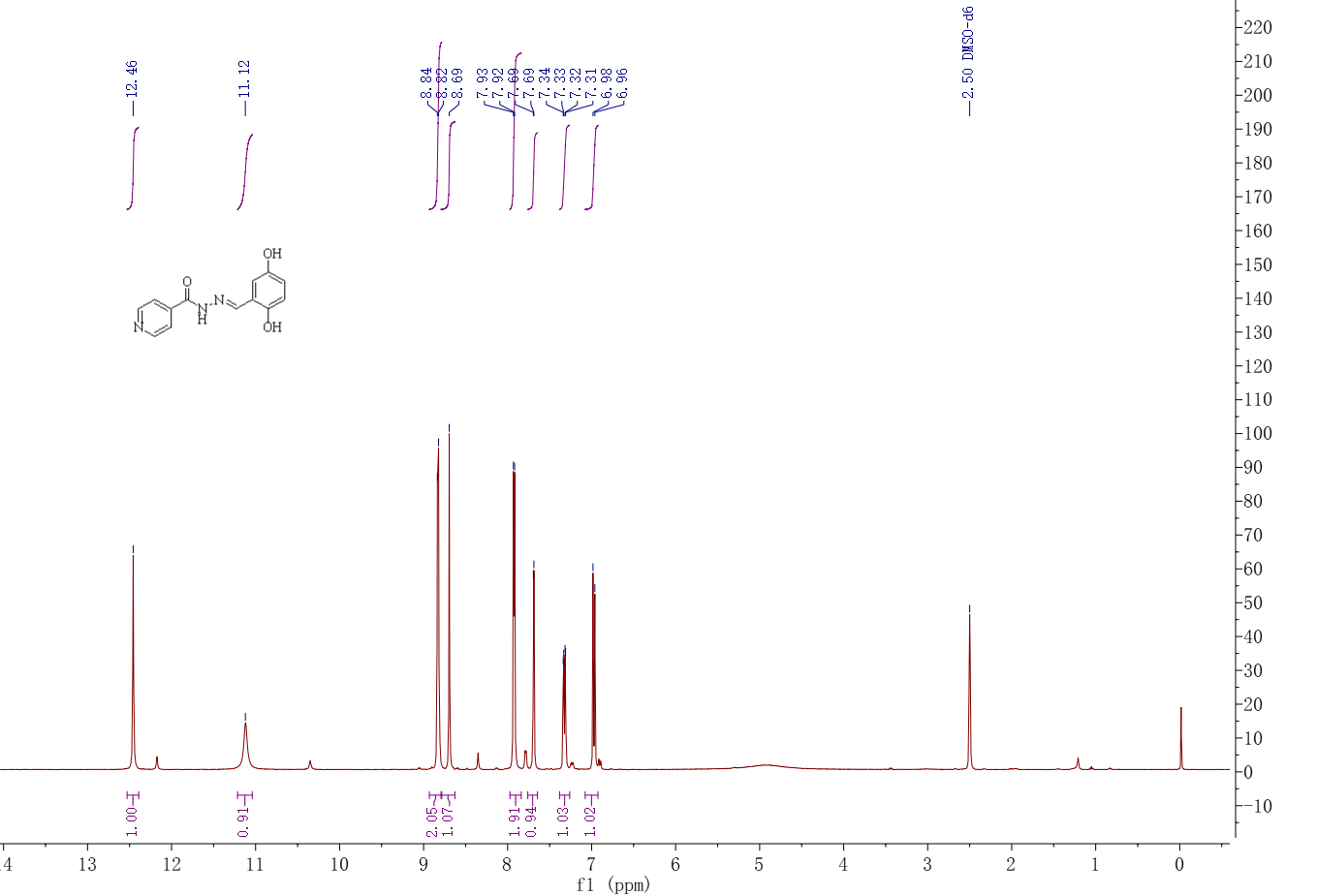


Fig. *^1^H NMR of* **C2** (400 MHz, DMSO)


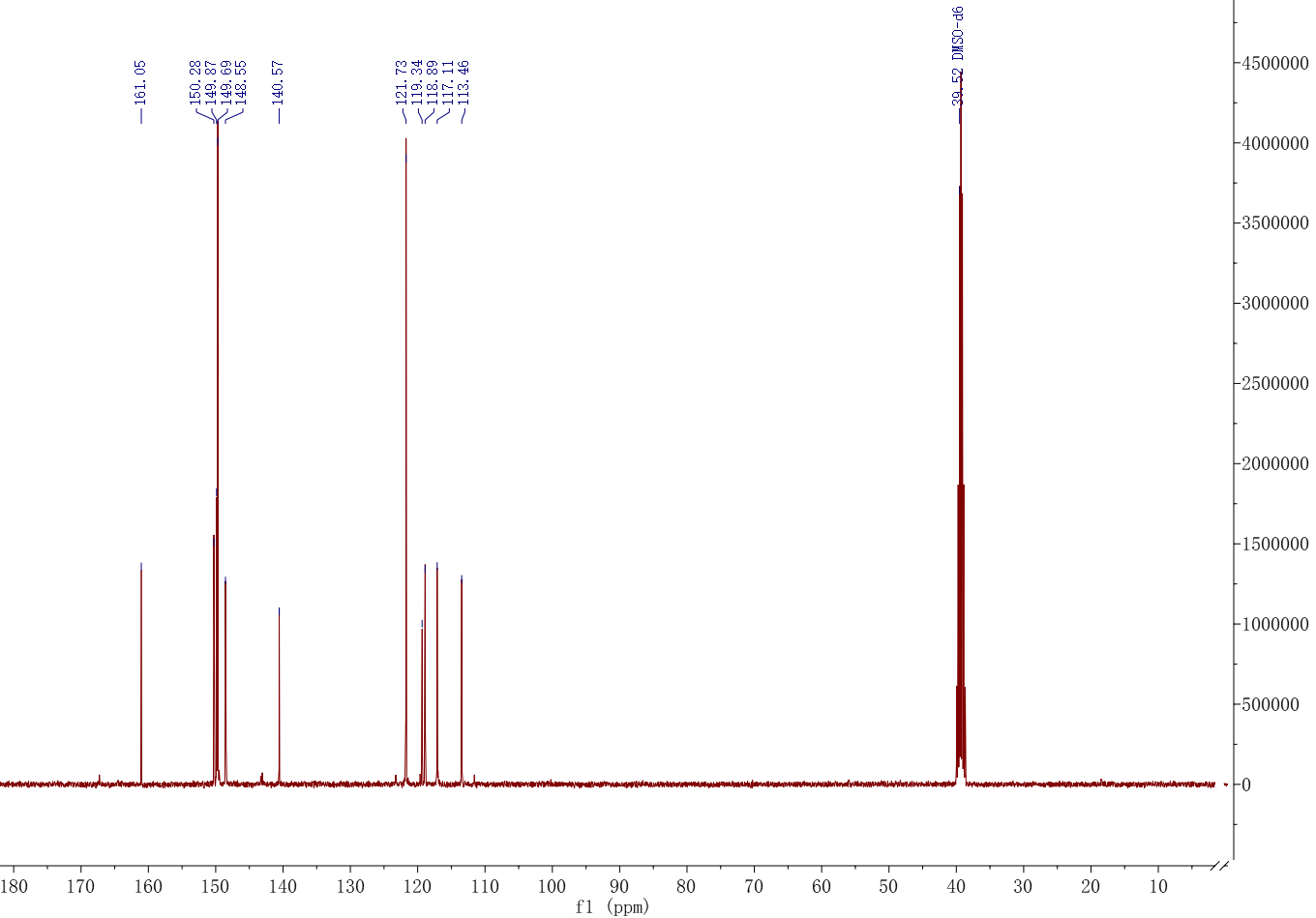


Fig. *^13^C NMR of* **C2** (100 MHz, DMSO)


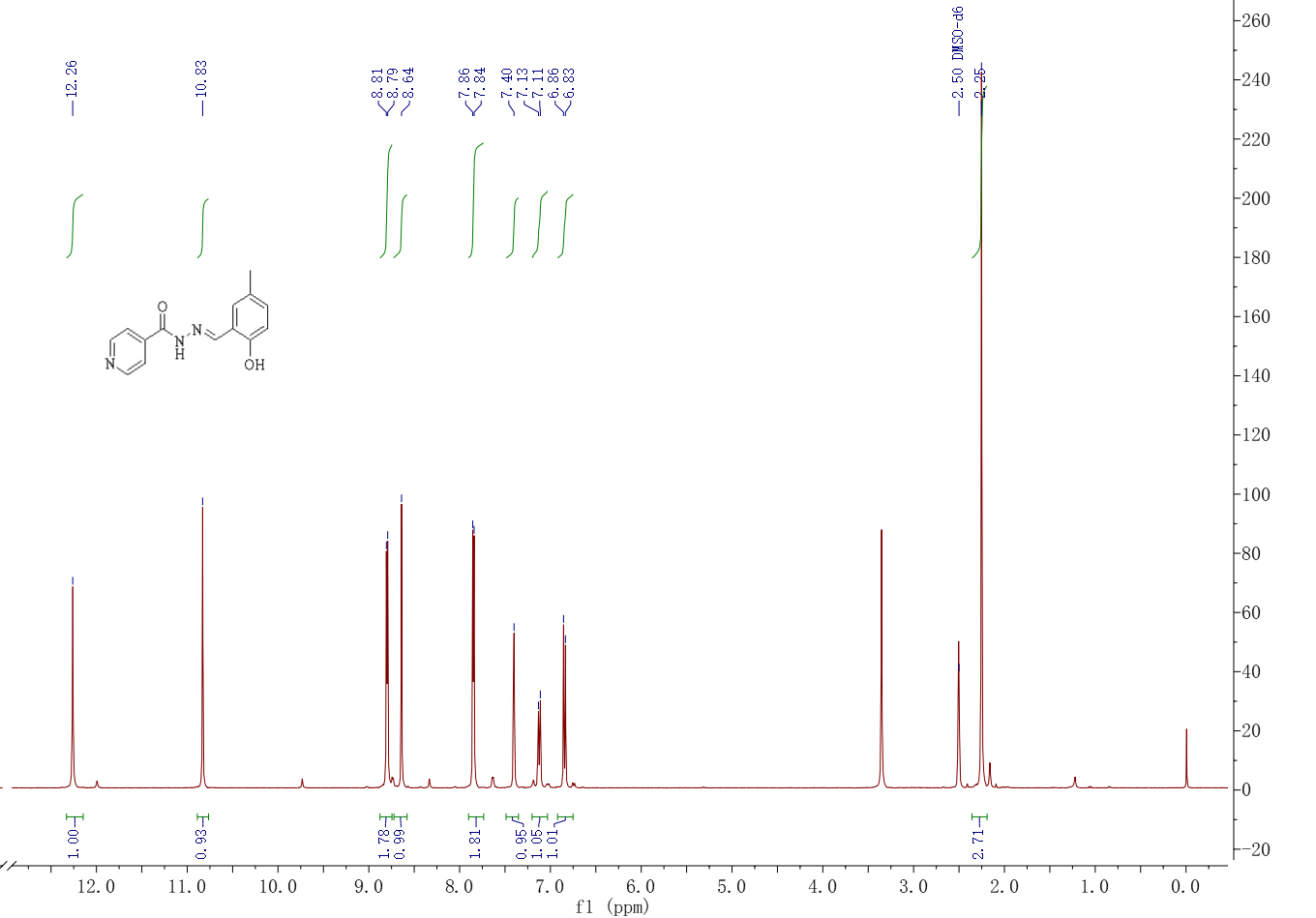


Fig. *^1^H NMR of* **C3** (400 MHz, DMSO)


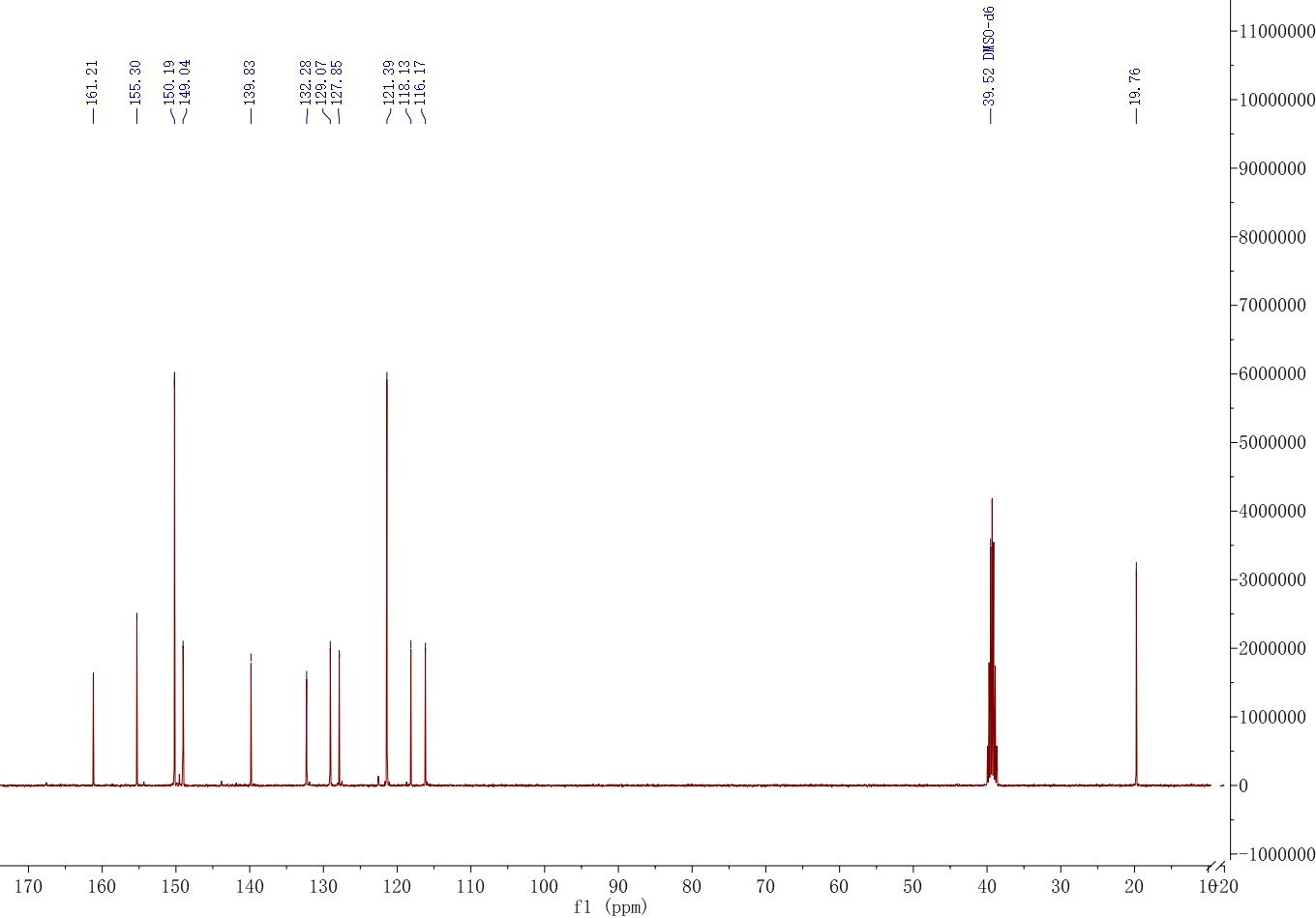


Fig. *^13^C NMR of* **C3** (100 MHz, DMSO)


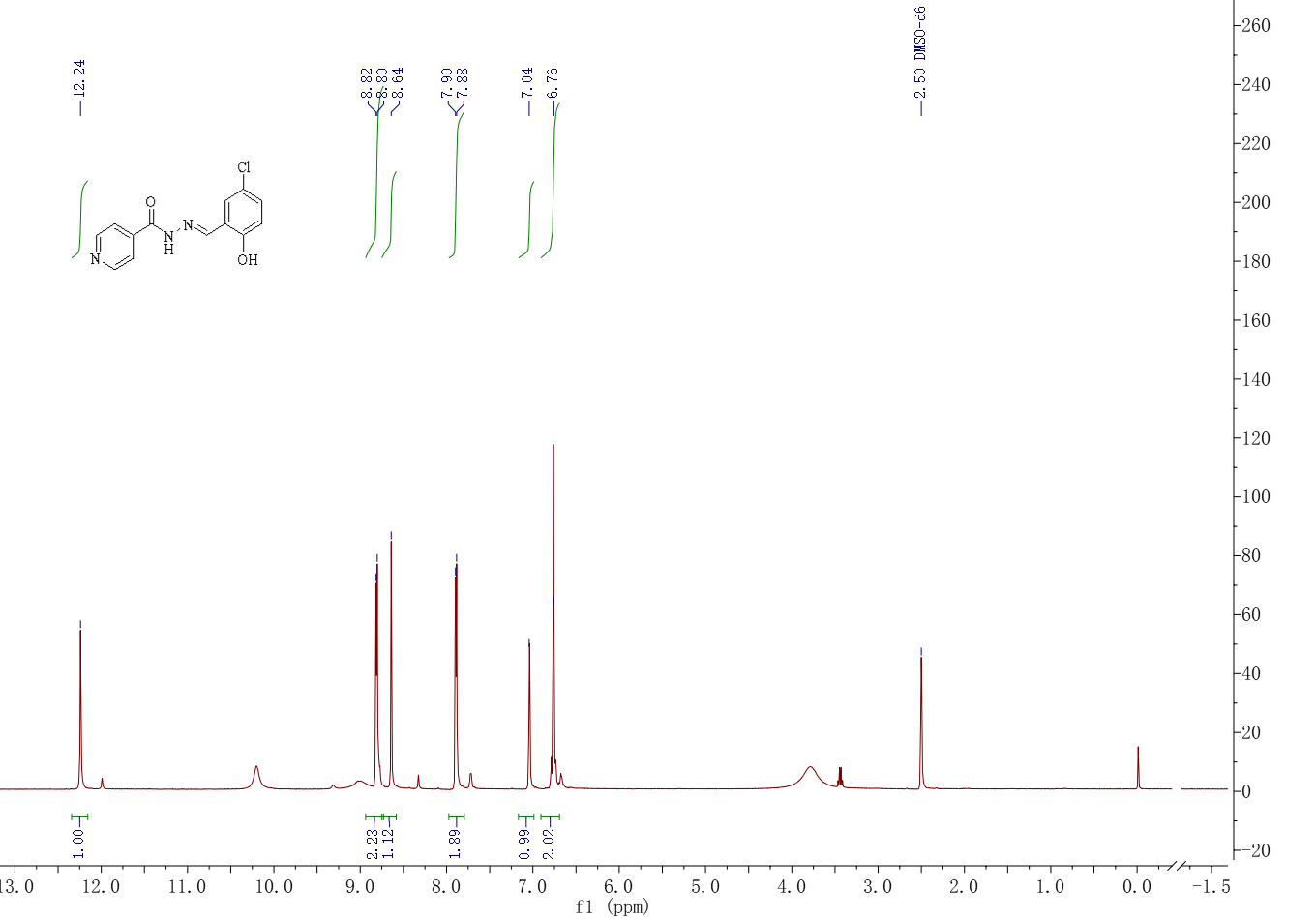


Fig. *^1^H NMR of* **C4** (400 MHz, DMSO)


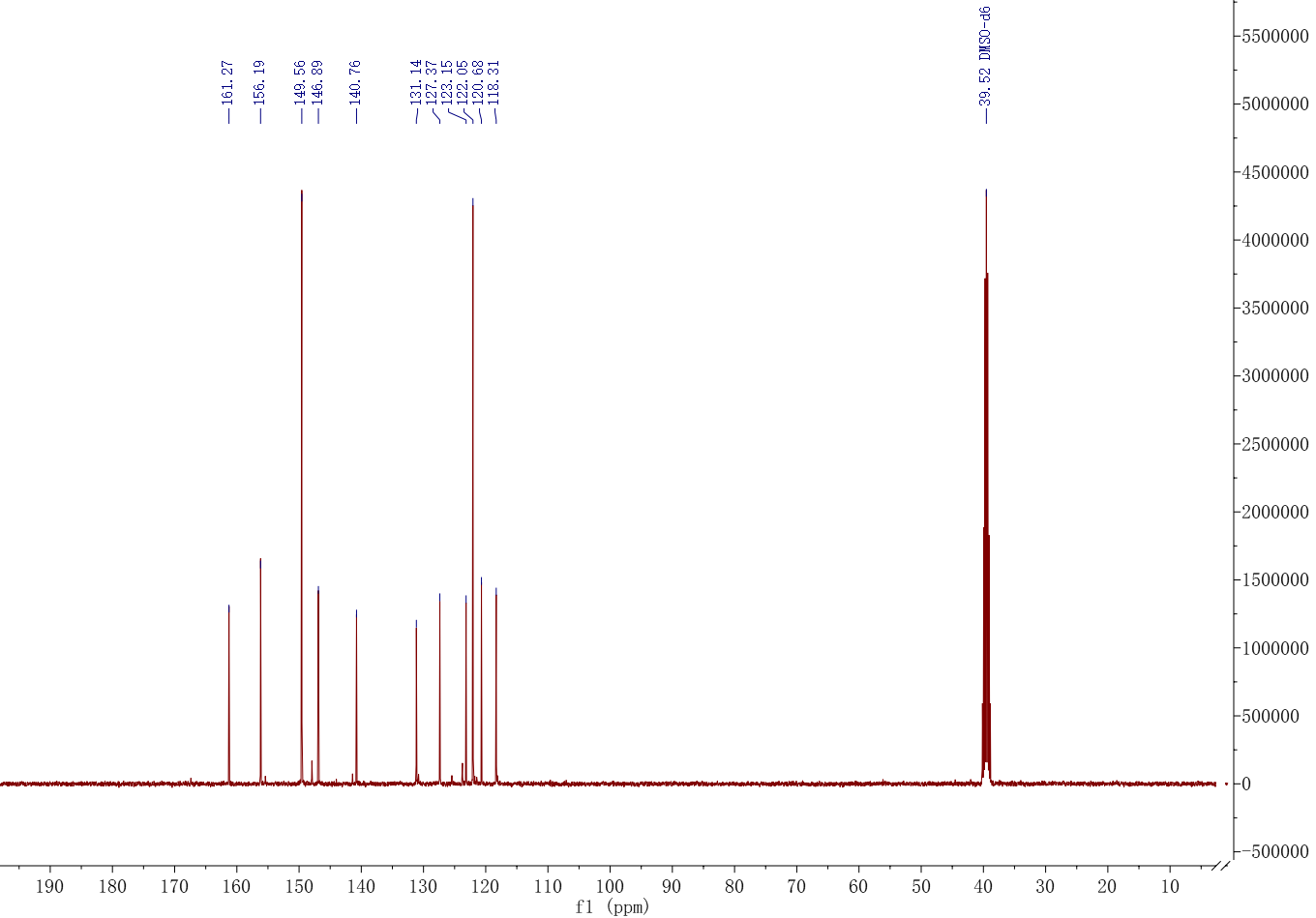


Fig. *^13^C NMR of* **C4** (100 MHz, DMSO)


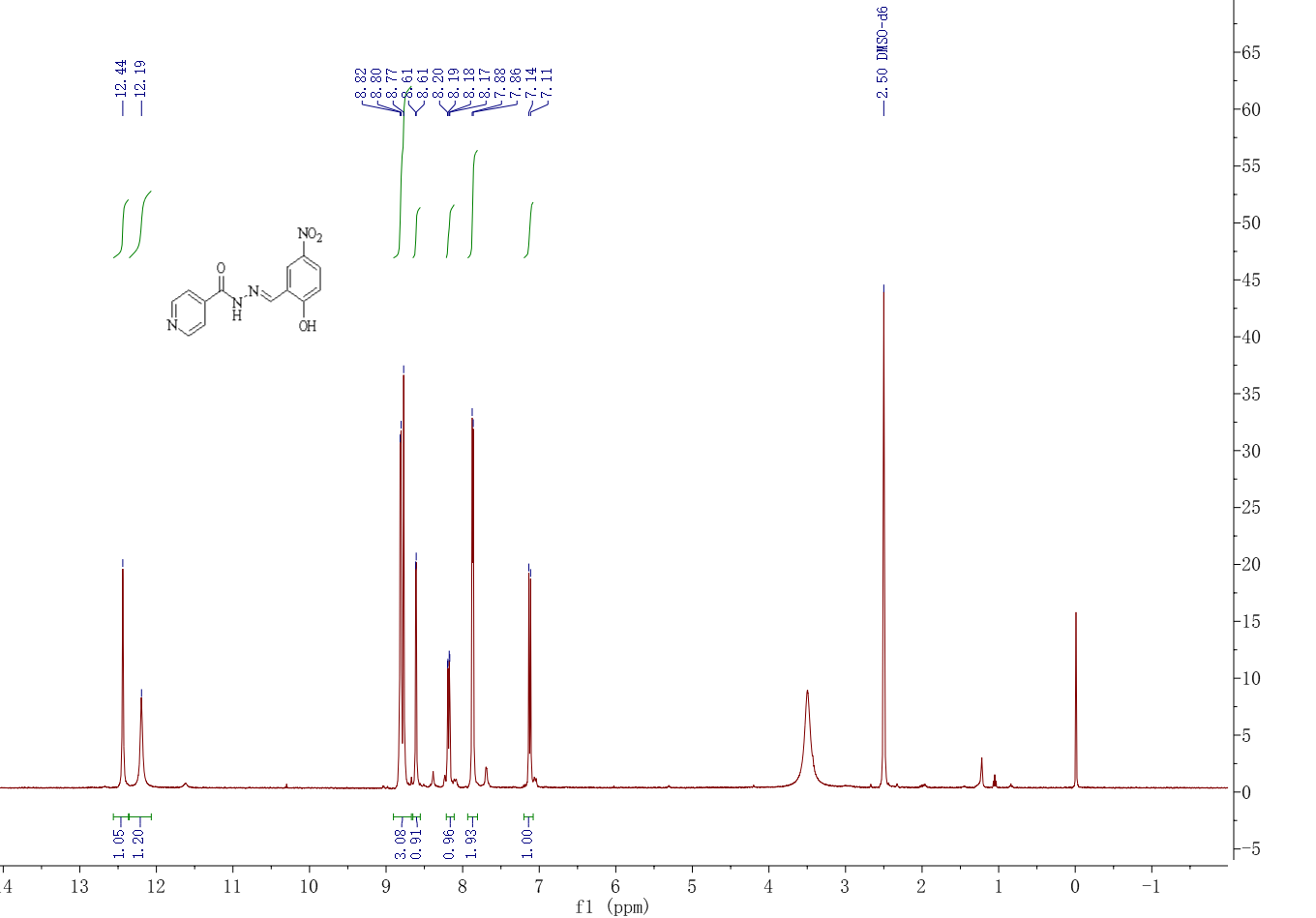


Fig. *^1^H NMR of* **C5** (400 MHz, DMSO)


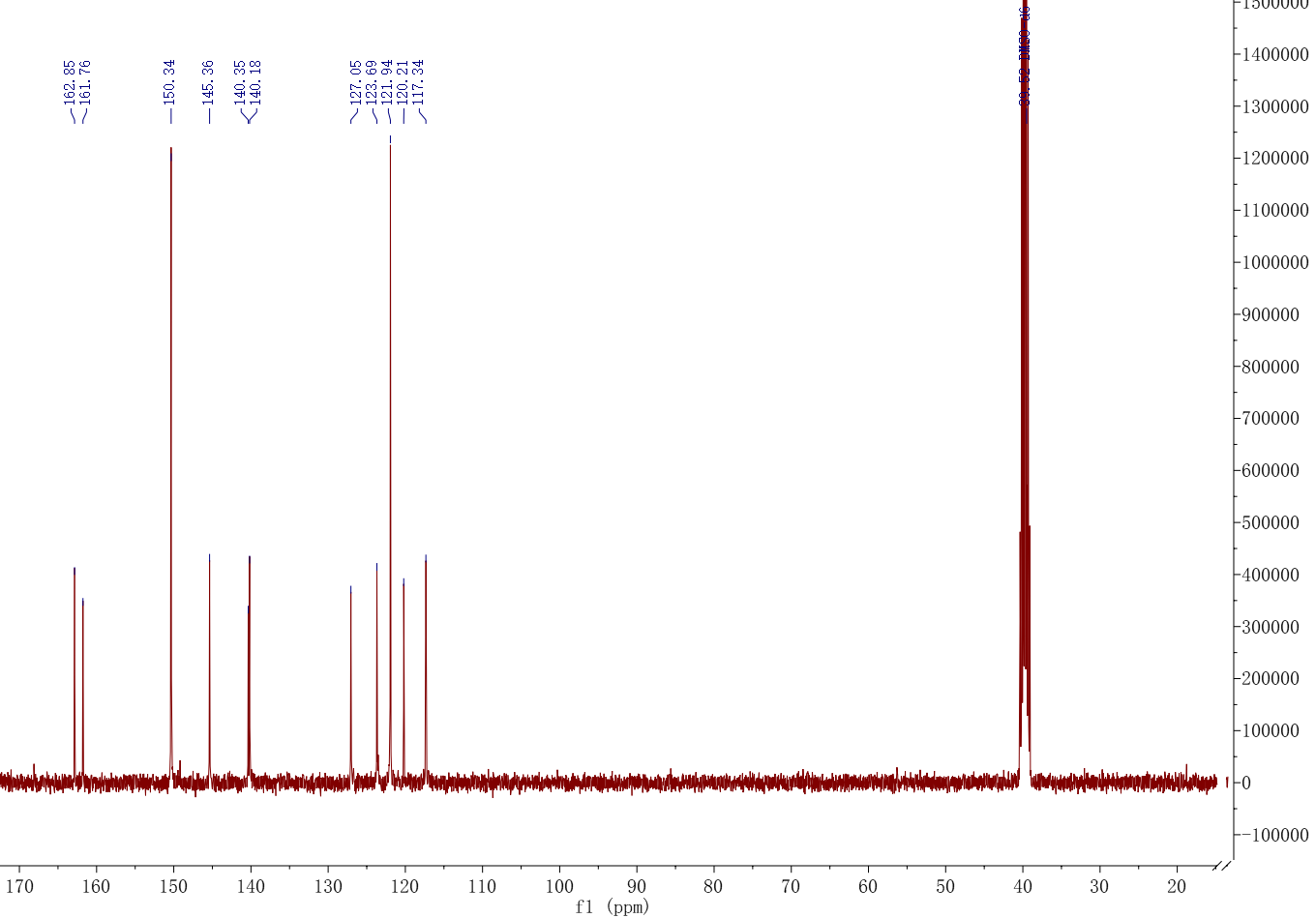


Fig. *^13^C NMR of* **C5** (100 MHz, DMSO)


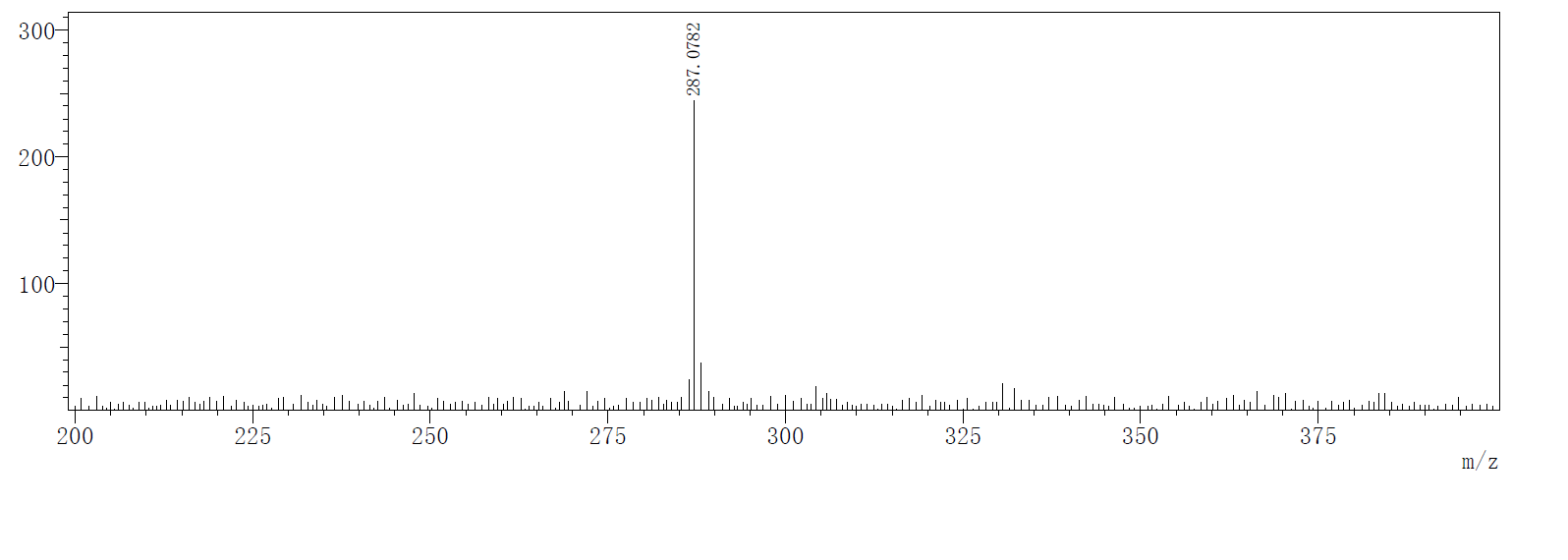


Fig. Mass spectrum of compound **C5**


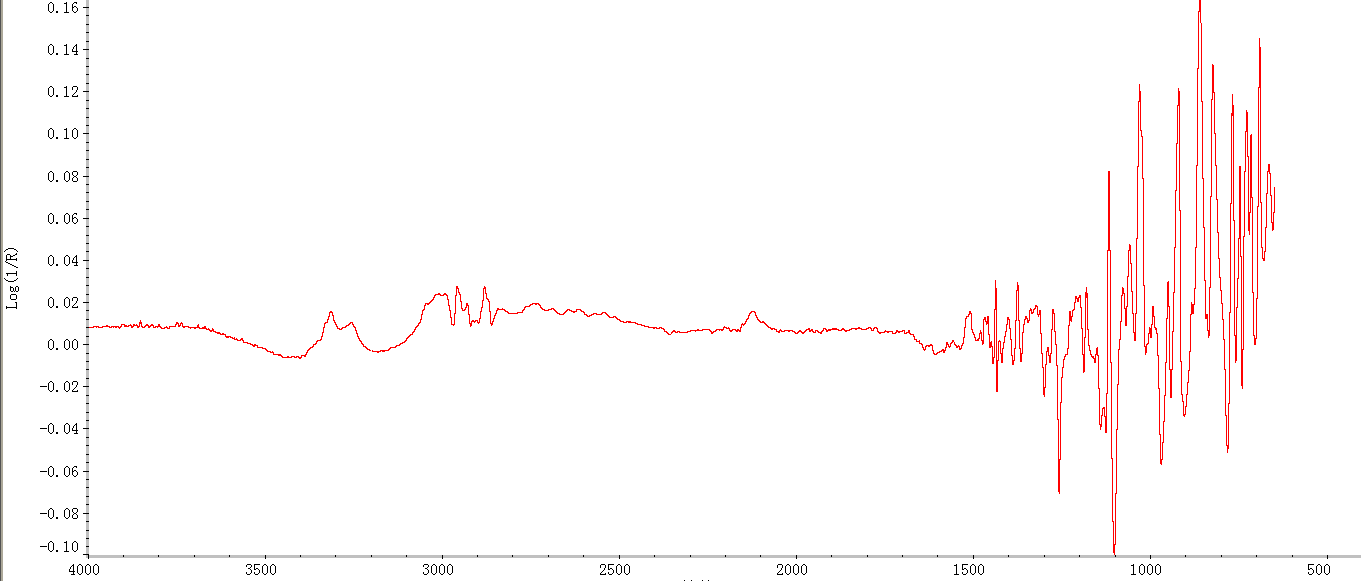


Fig. The FTIR spectrum of **C5**


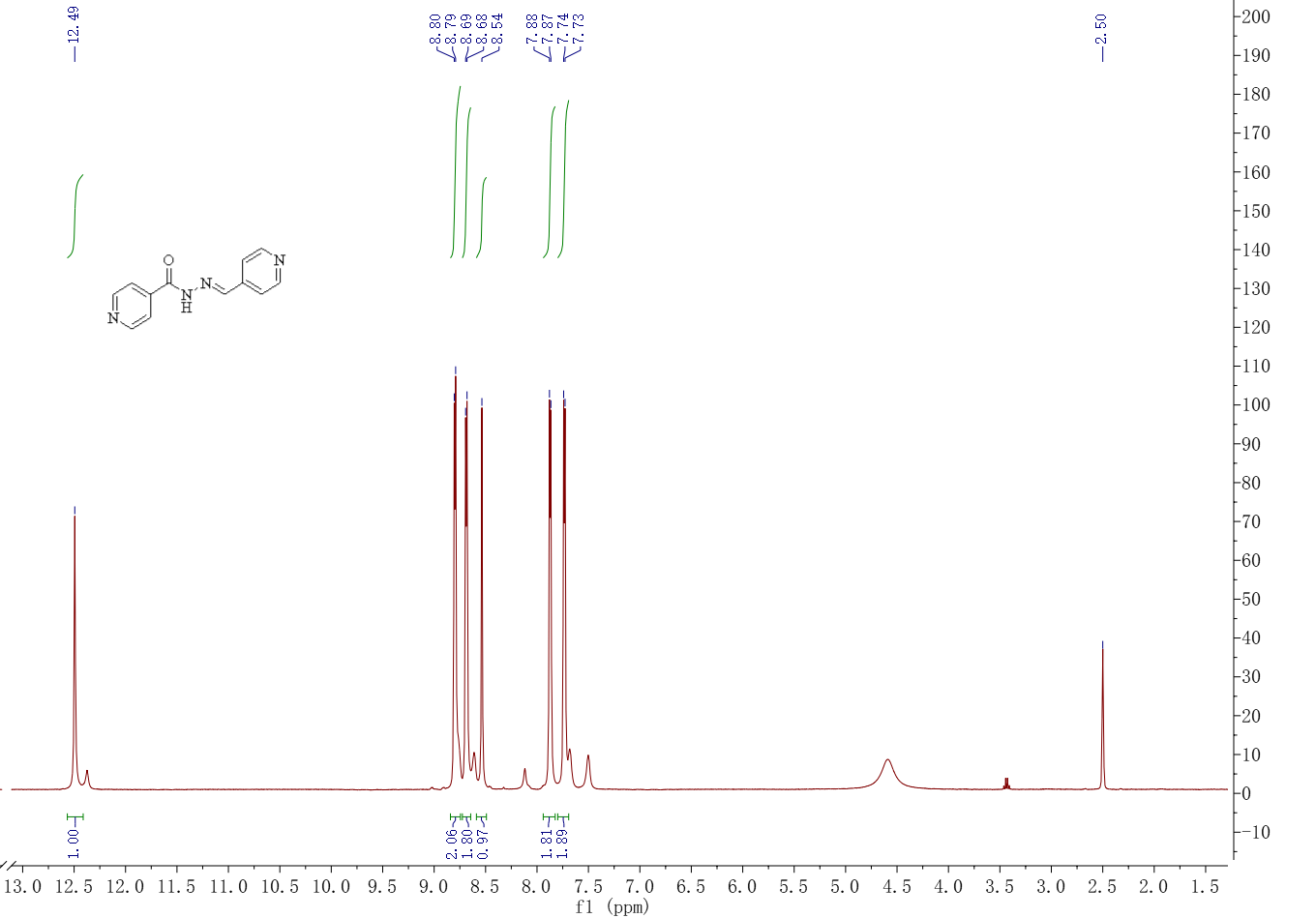


Fig. *^1^H NMR of* **C6** (400 MHz, DMSO)


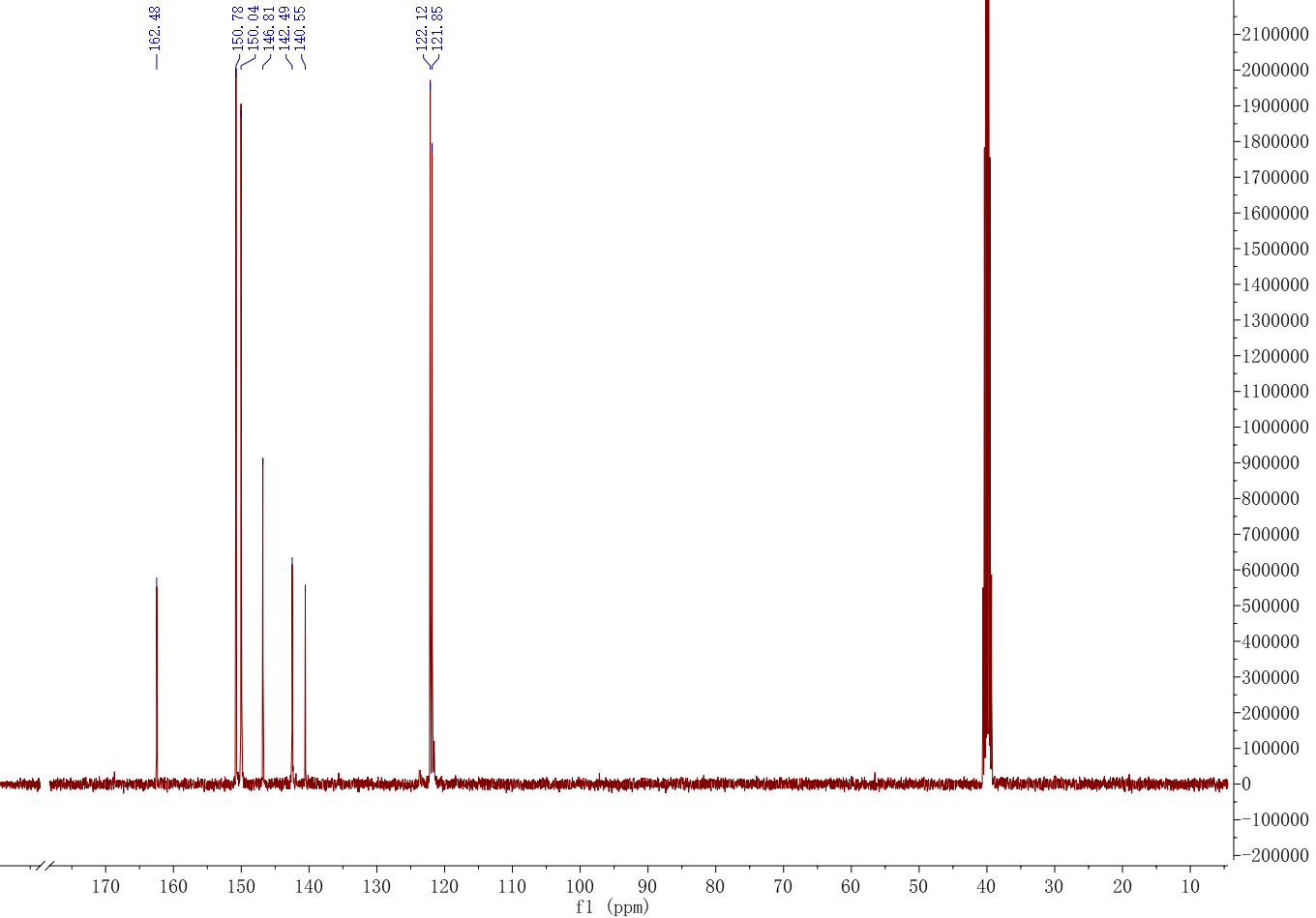


Fig. *^13^C NMR of* **C6** (100 MHz, DMSO)


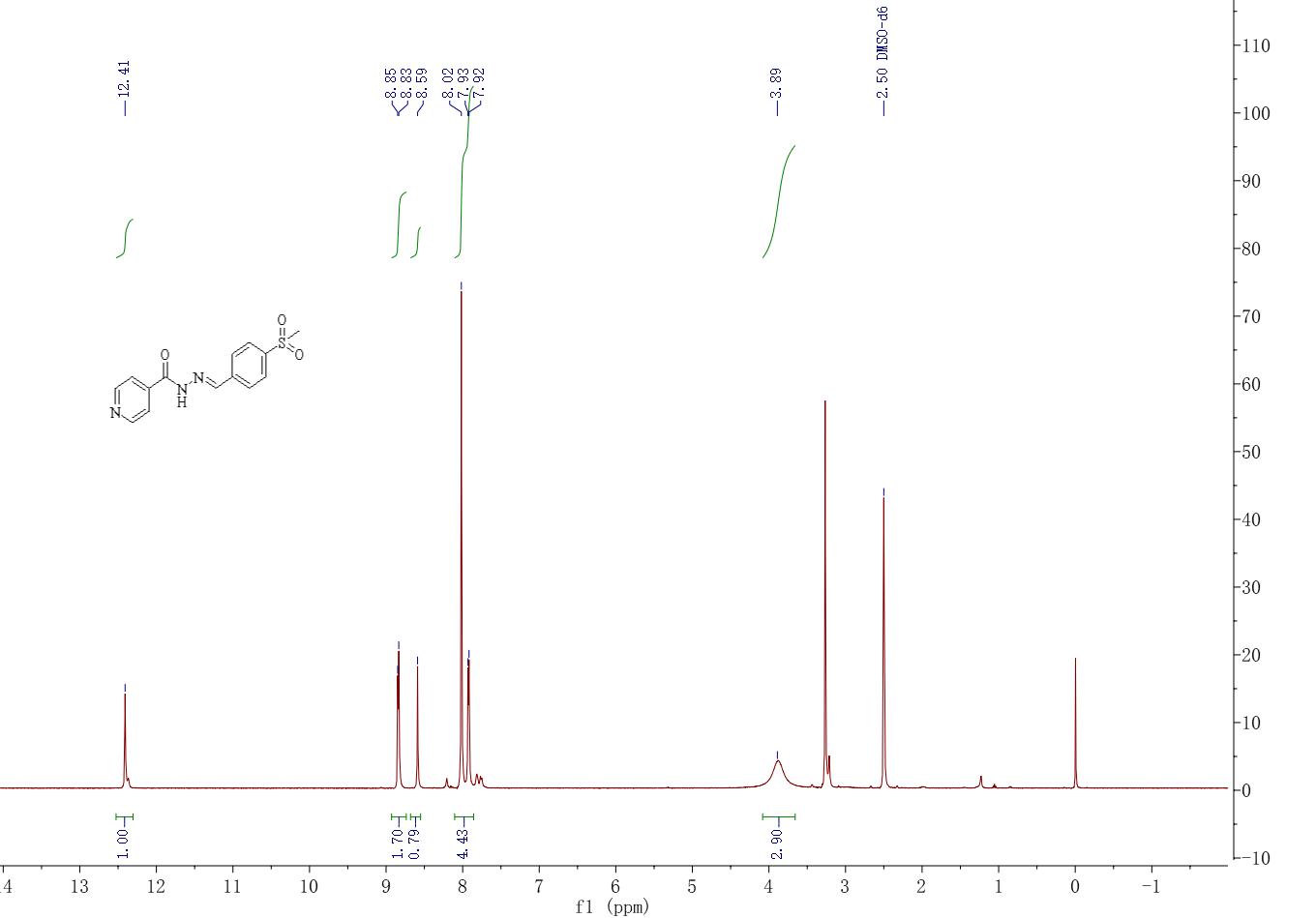


Fig. *^1^H NMR of* **C7** (400 MHz, DMSO)


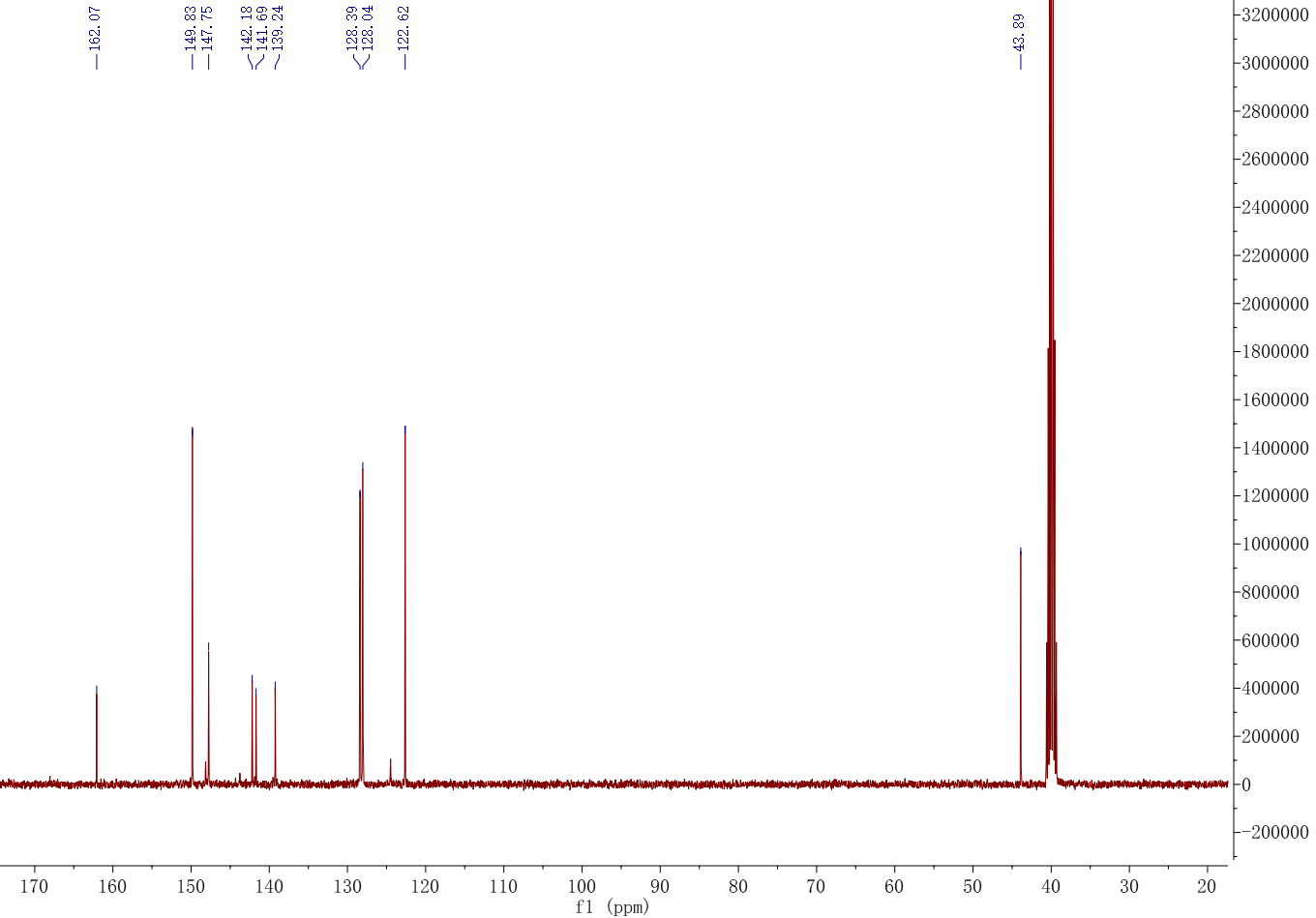


Fig. *^13^C NMR of* **C7** (100 MHz, DMSO)


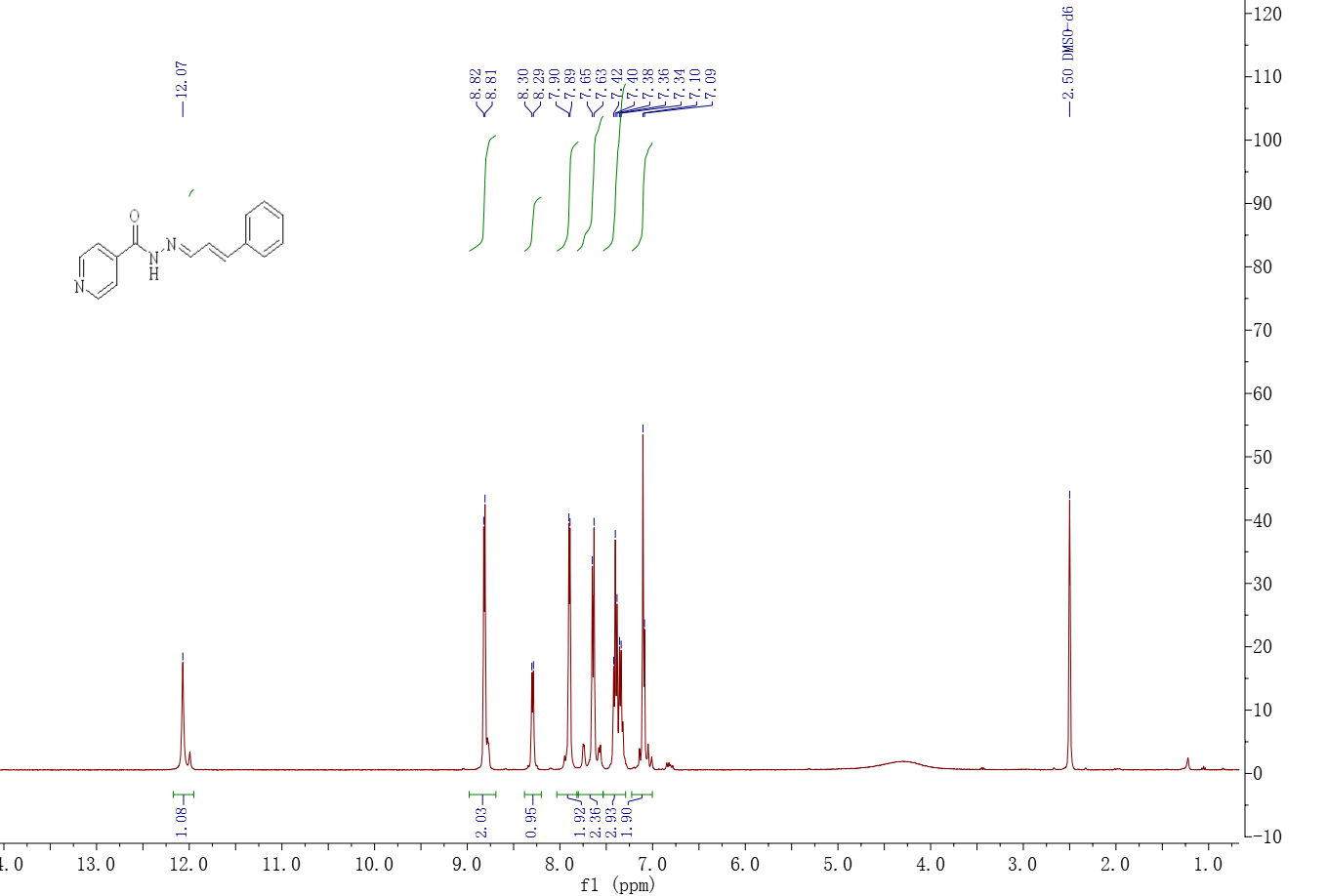


Fig. *^1^H NMR of* **C8** (400 MHz, DMSO)


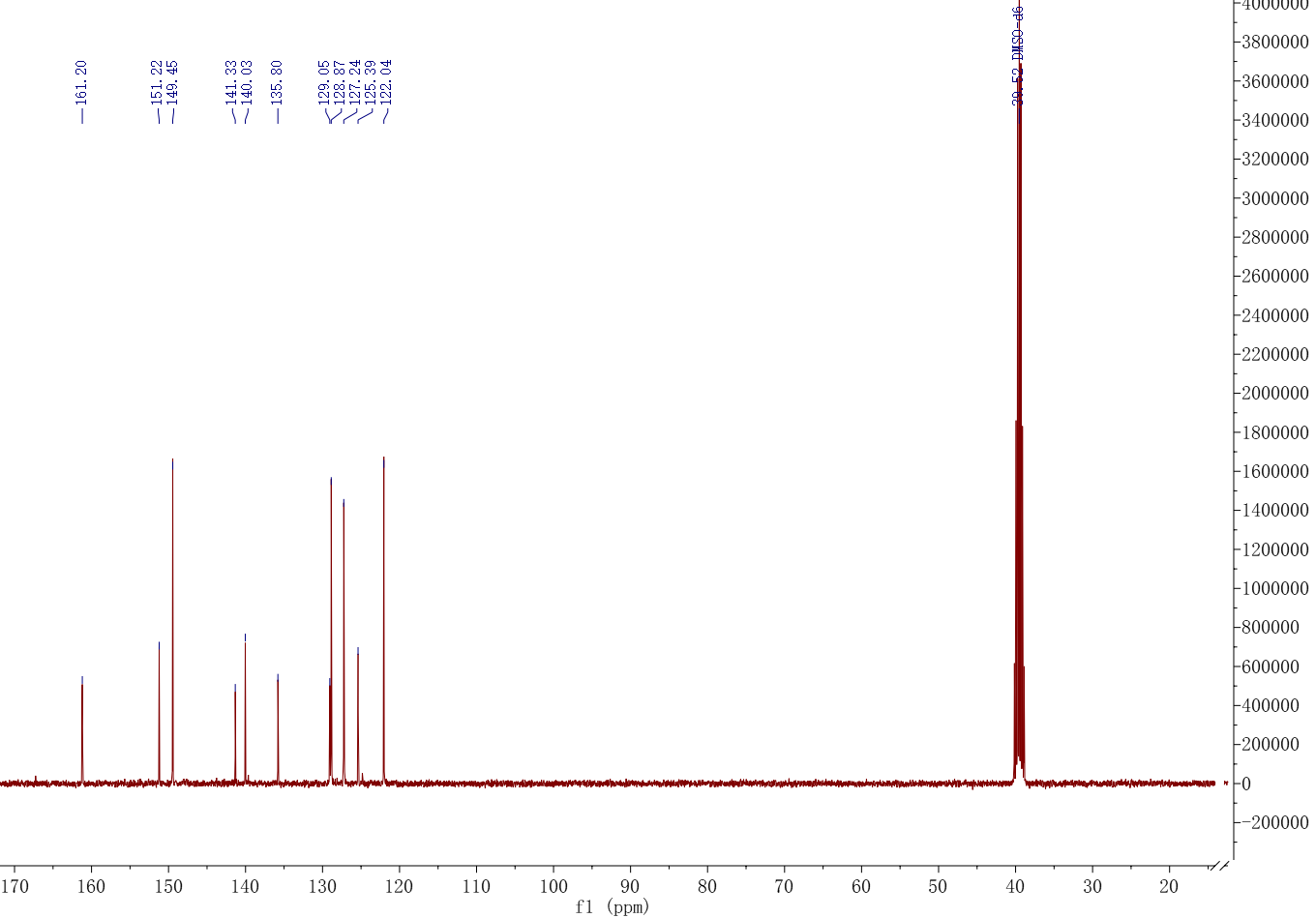


Fig. *^13^C NMR of* **C8** (100 MHz, DMSO)


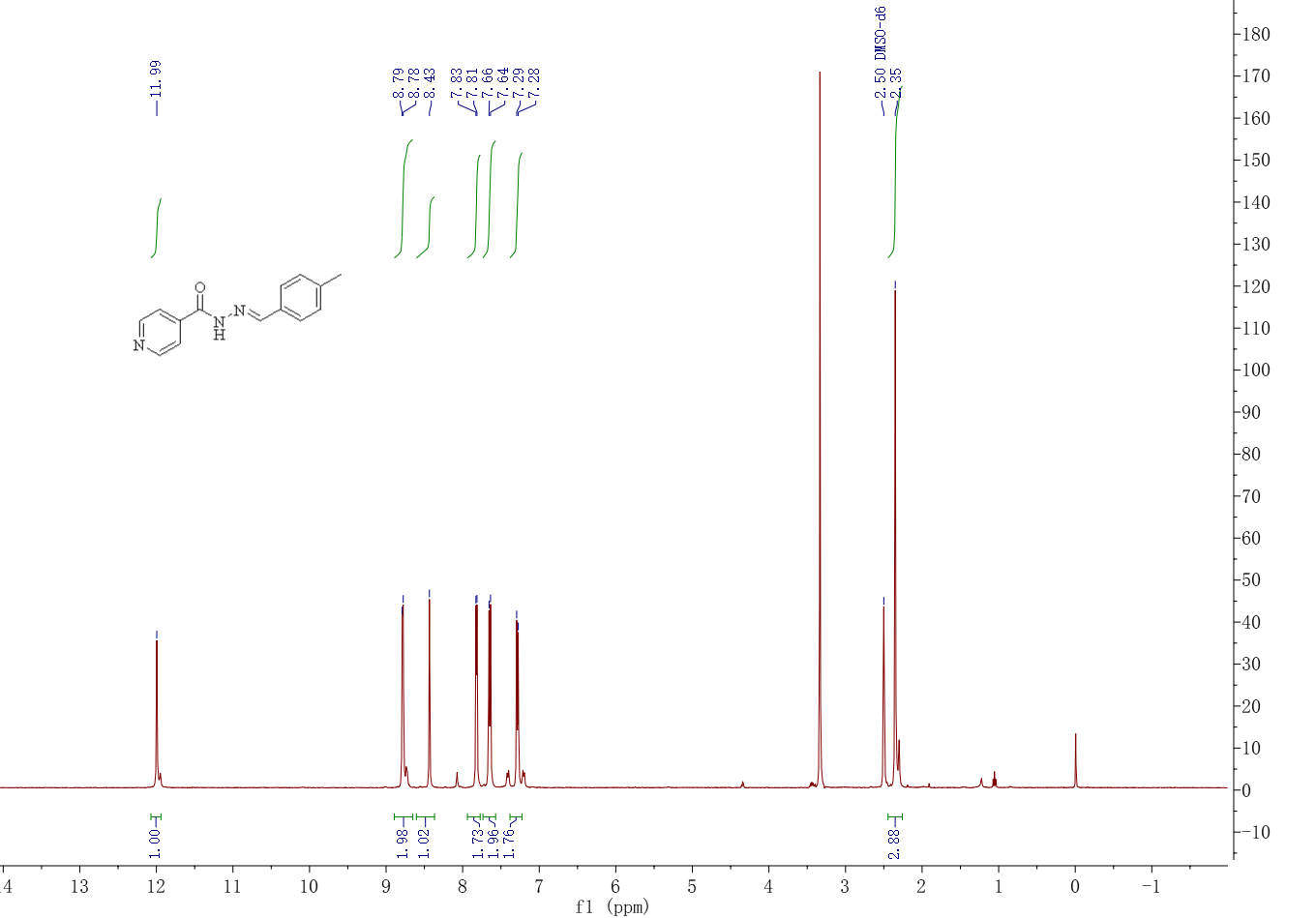


Fig. *^1^H NMR of* **C9** (400 MHz, DMSO)


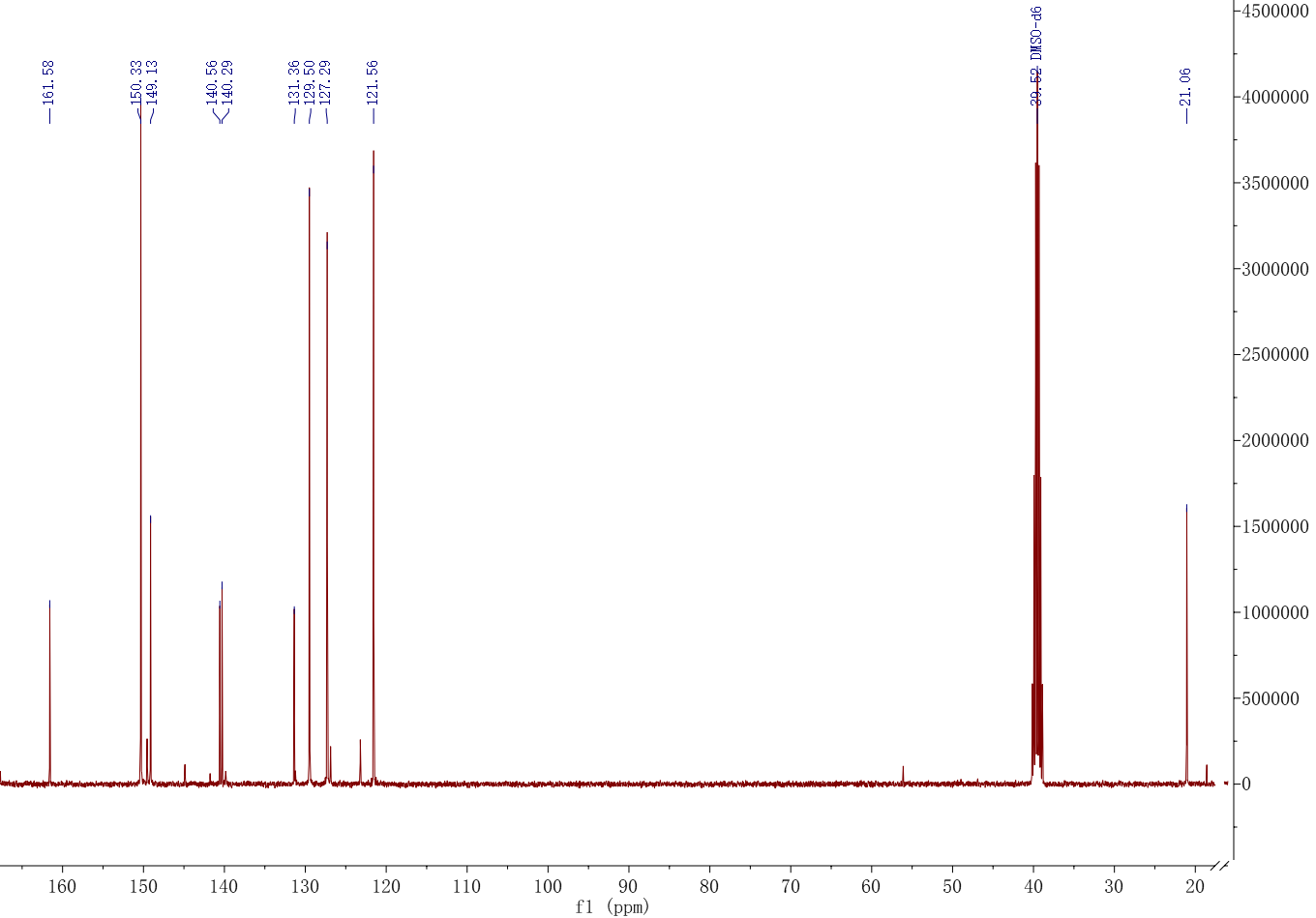


Fig. *^13^C NMR of* **C9** (100 MHz, DMSO)


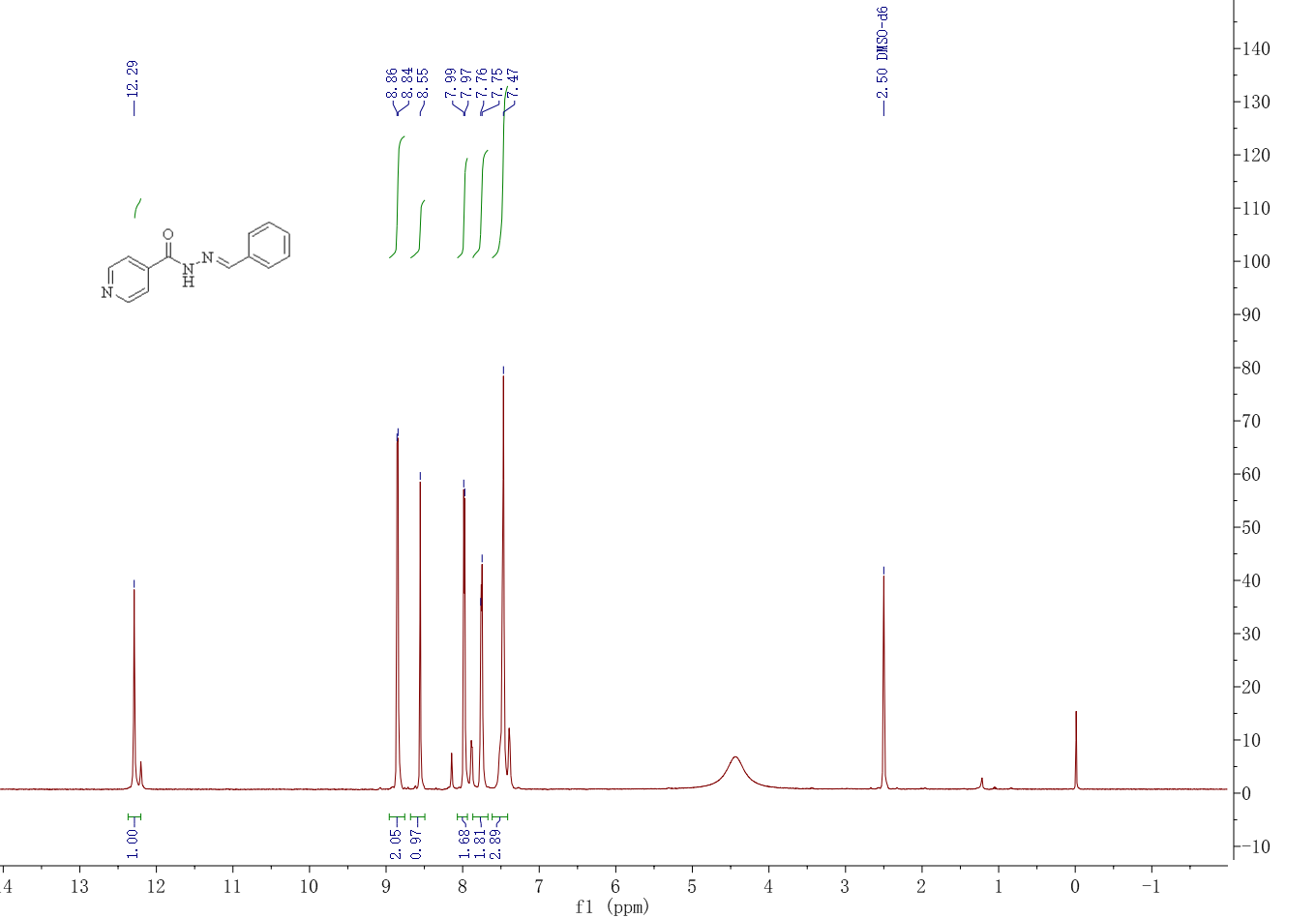


Fig. *^1^H NMR of* **C10** (400 MHz, DMSO)


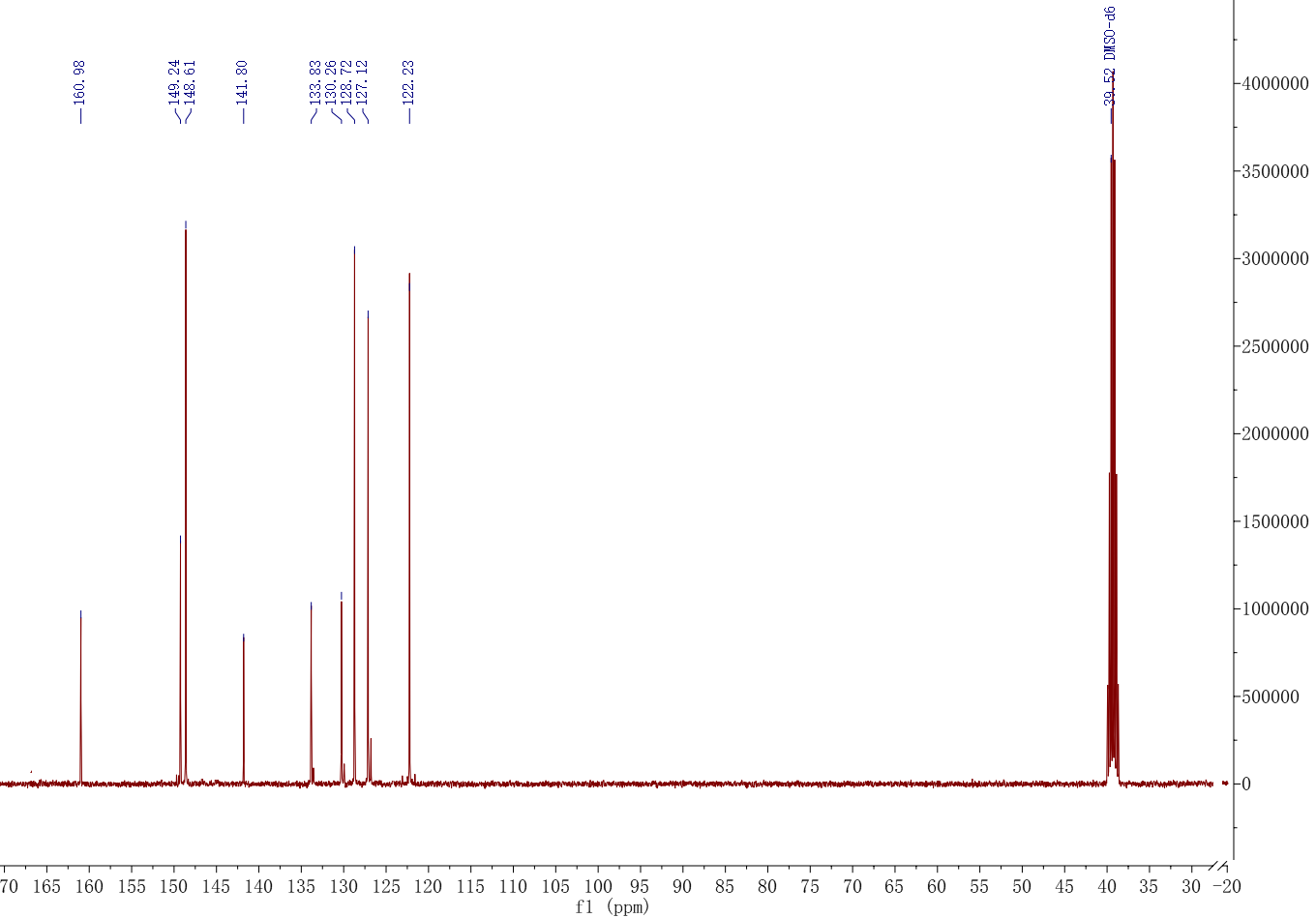


Fig. *^13^C NMR of* **C10** (100 MHz, DMSO)


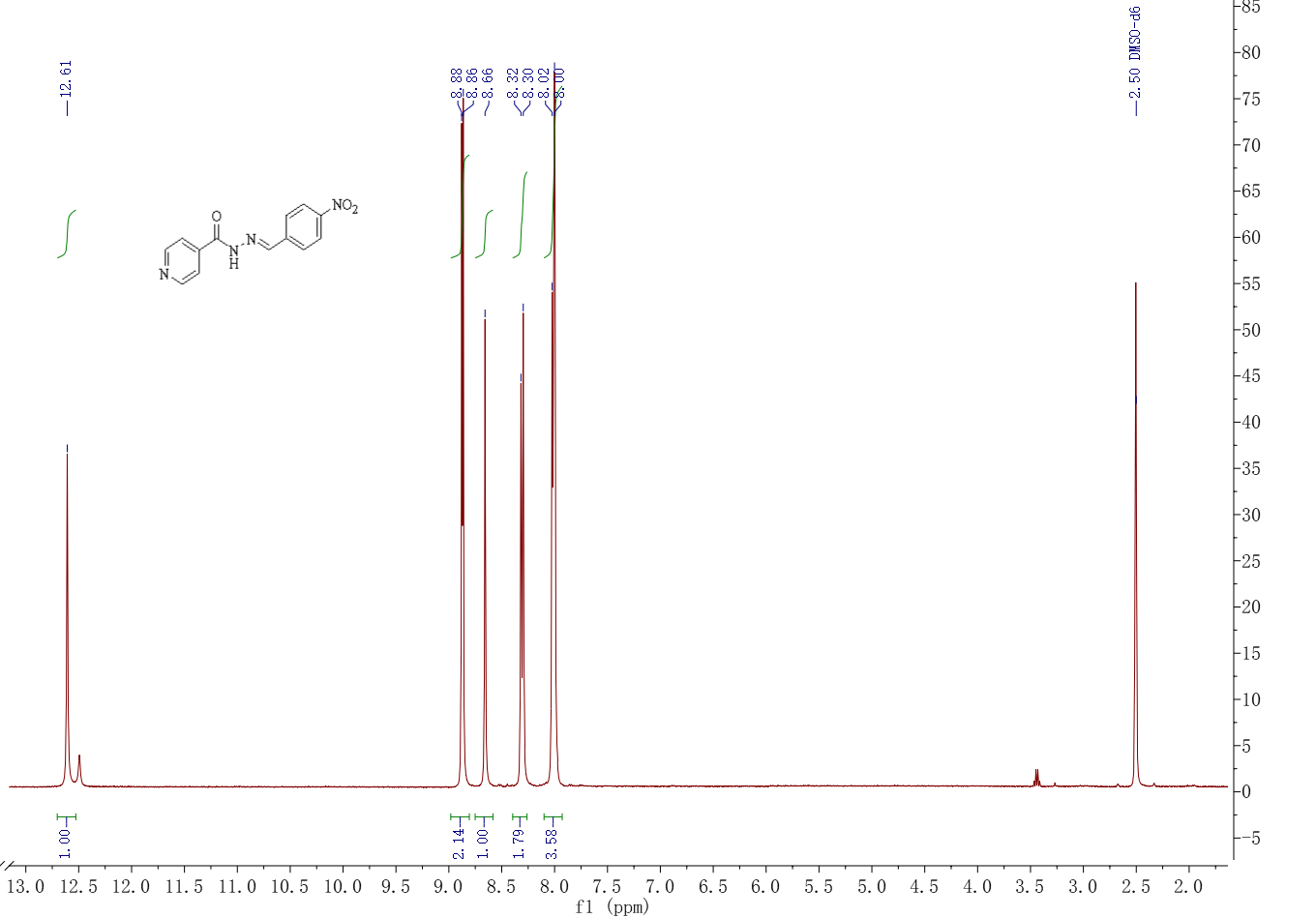


Fig. *^1^H NMR of* **C11** (400 MHz, DMSO)


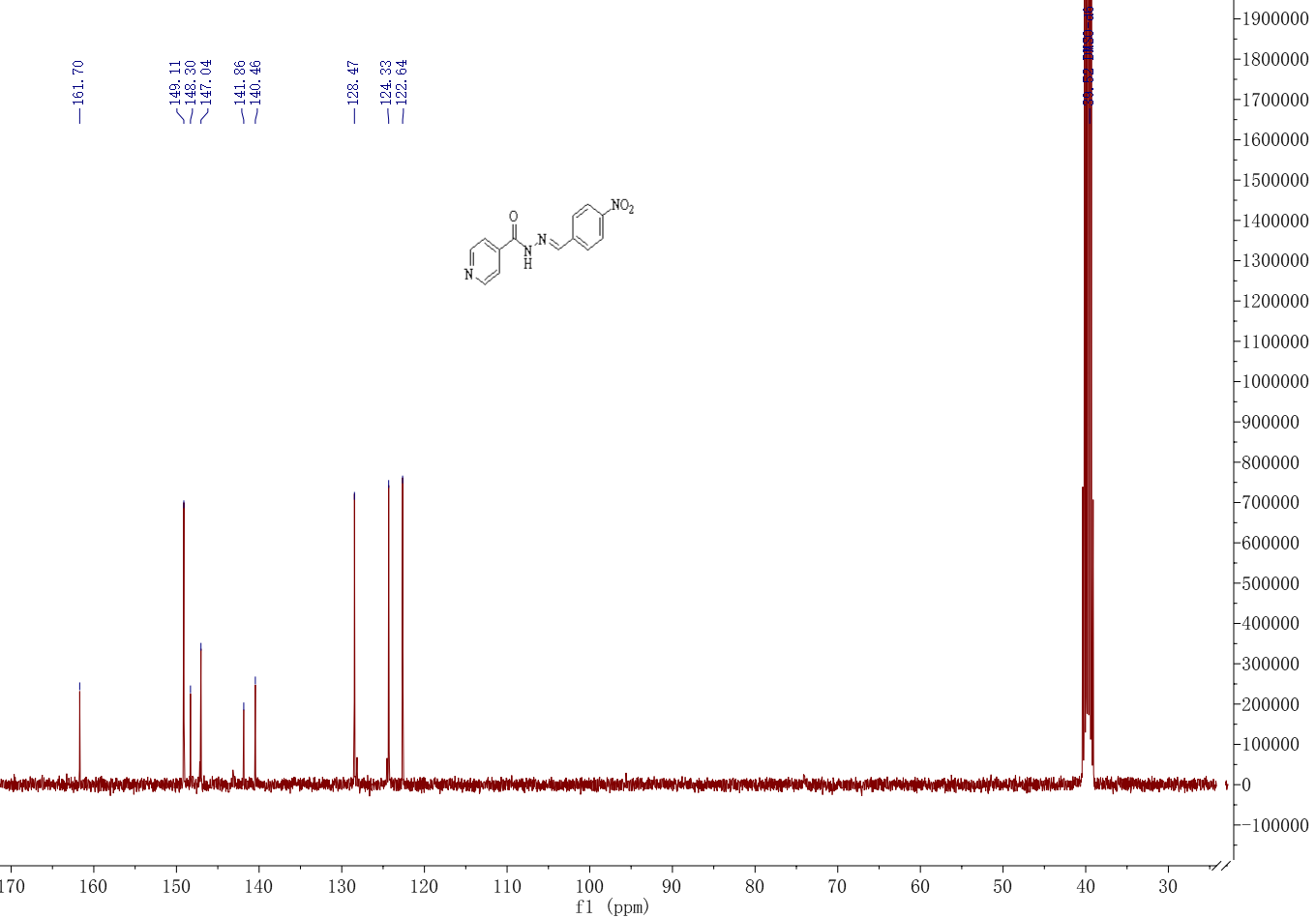


Fig. *^13^C NMR of* **C11** (100 MHz, DMSO)


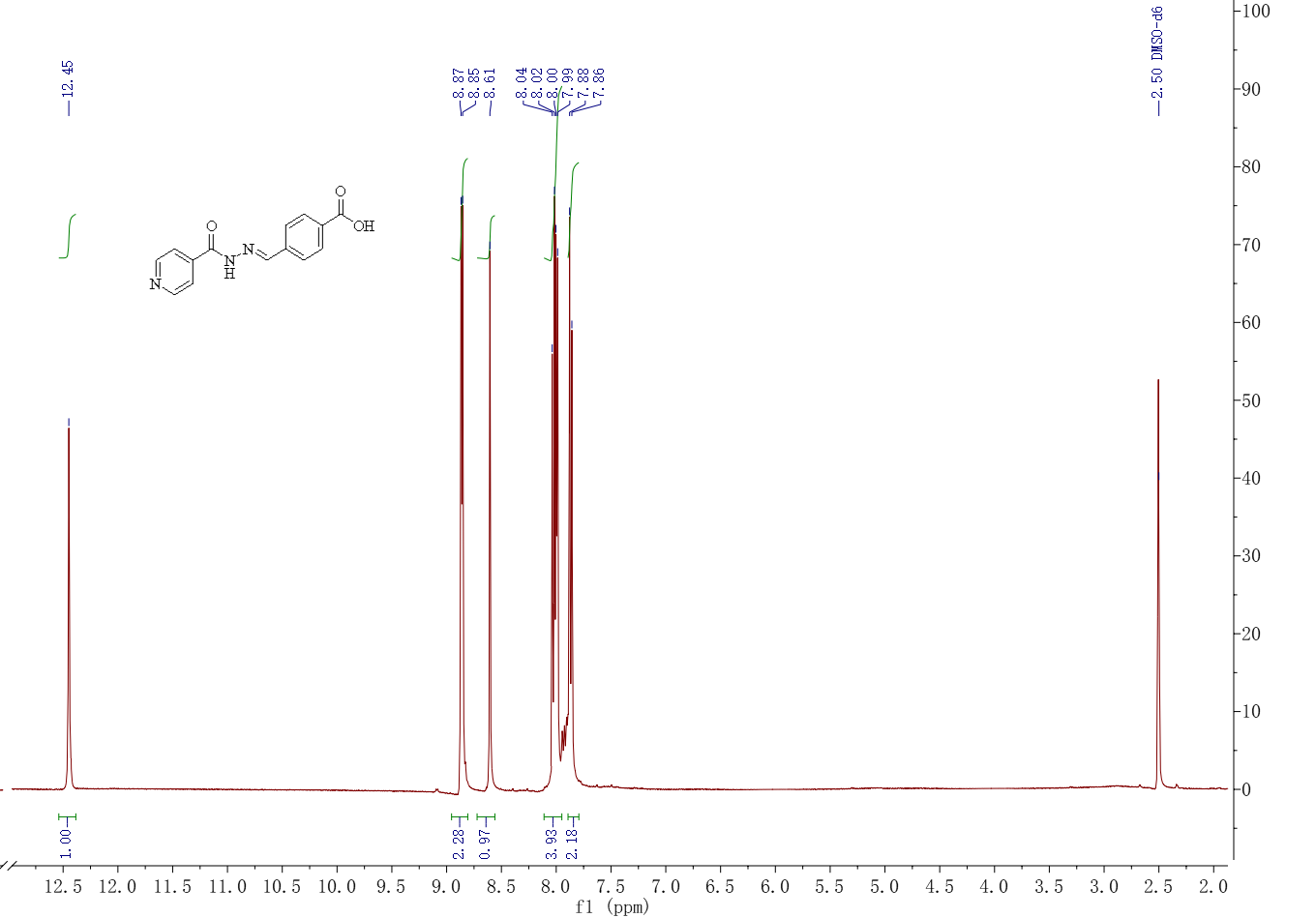


Fig. *^1^H NMR of* **C12** (400 MHz, DMSO)


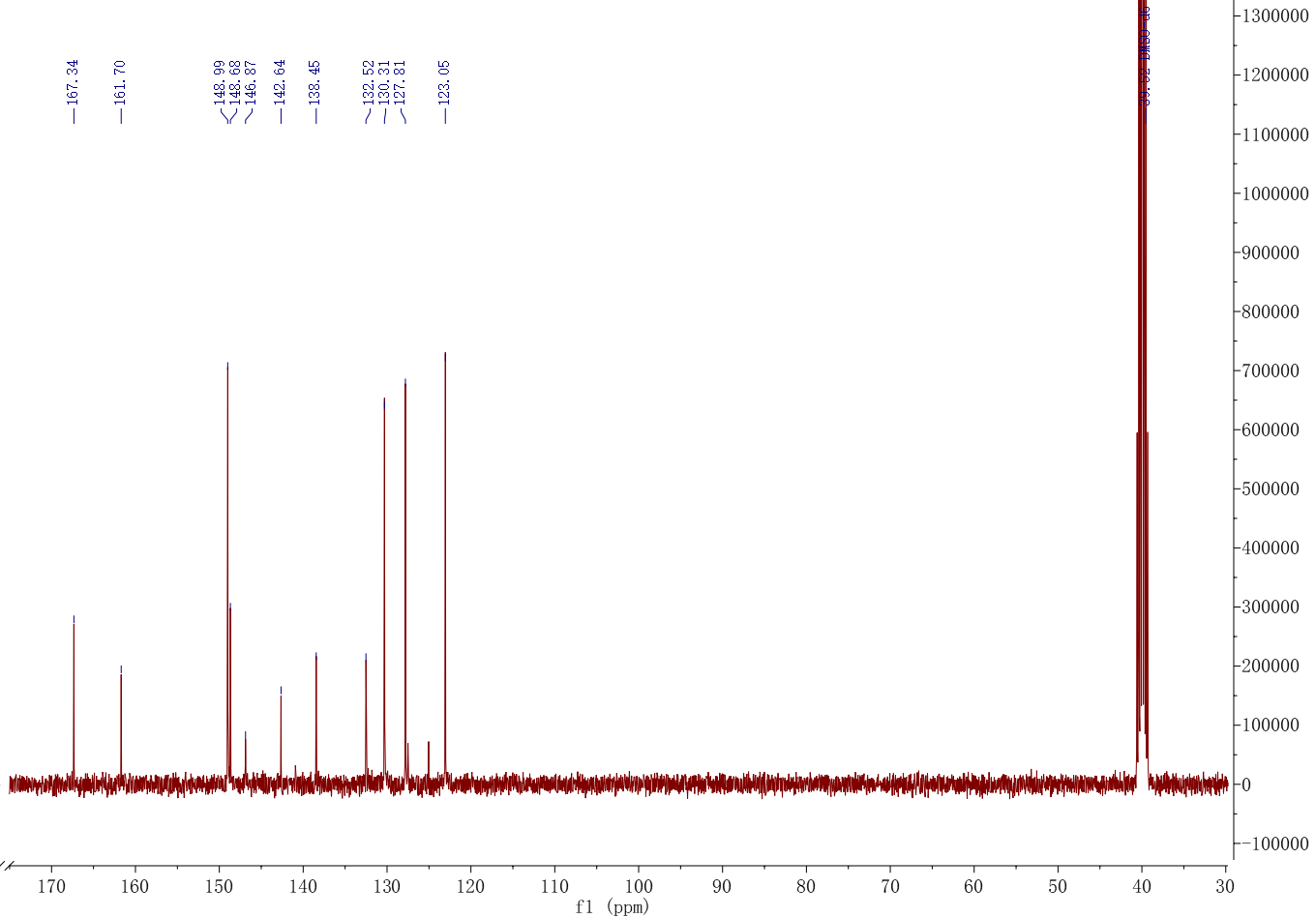


Fig. *^13^C NMR of* **C12** (100 MHz, DMSO)


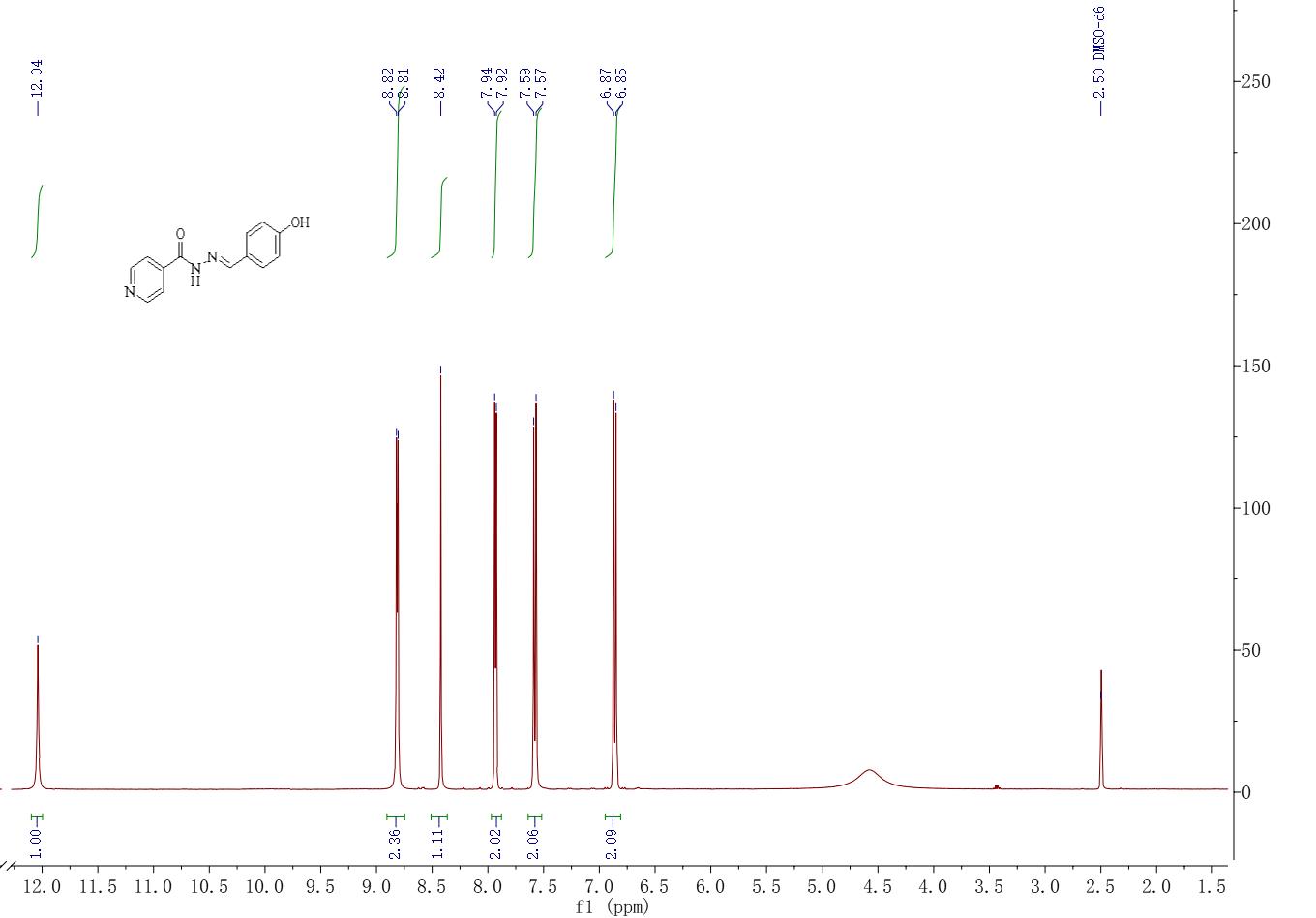


Fig. *^1^H NMR of* **C13** (400 MHz, DMSO)


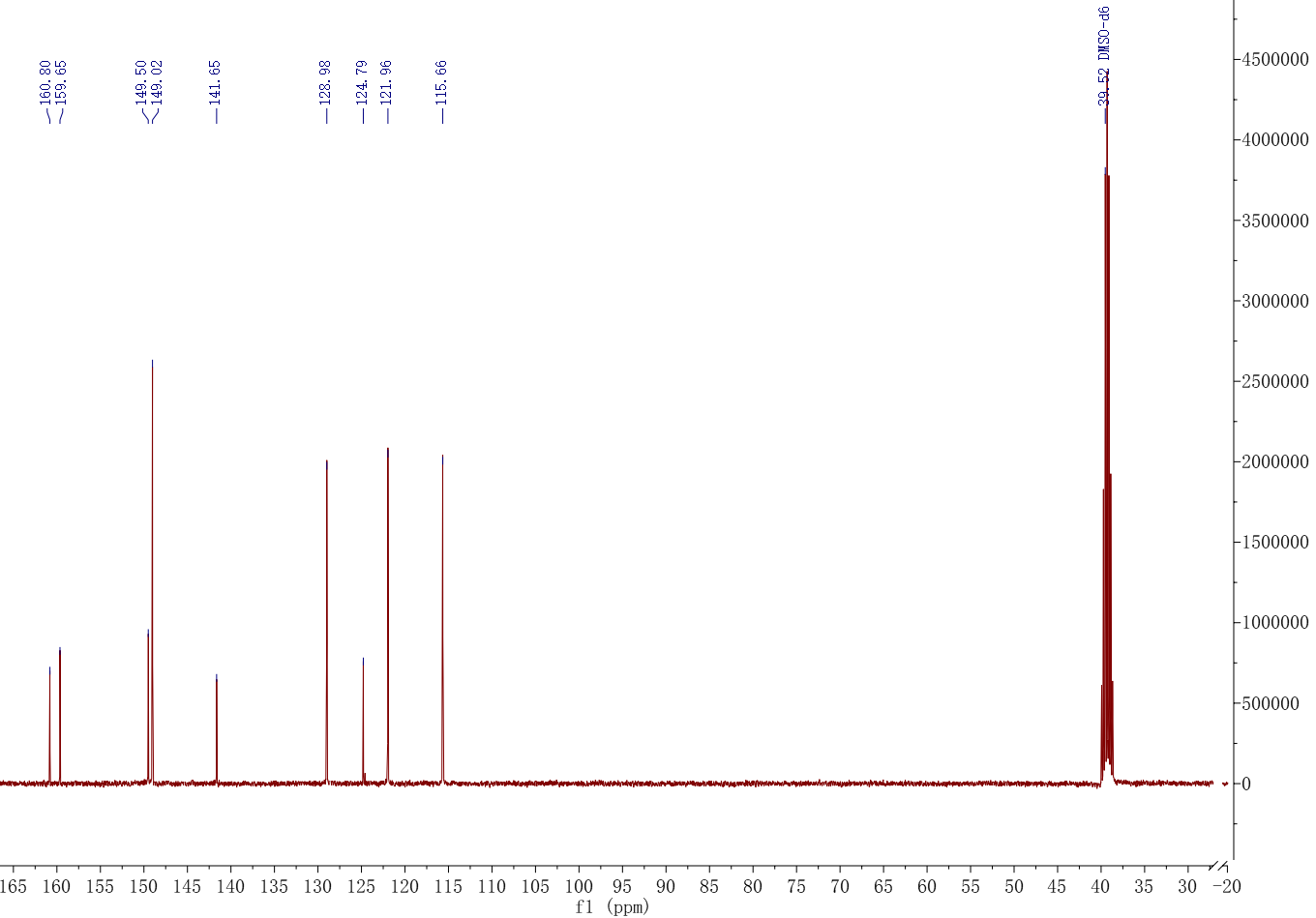


Fig. *^13^C NMR of* **C13** (100 MHz, DMSO)


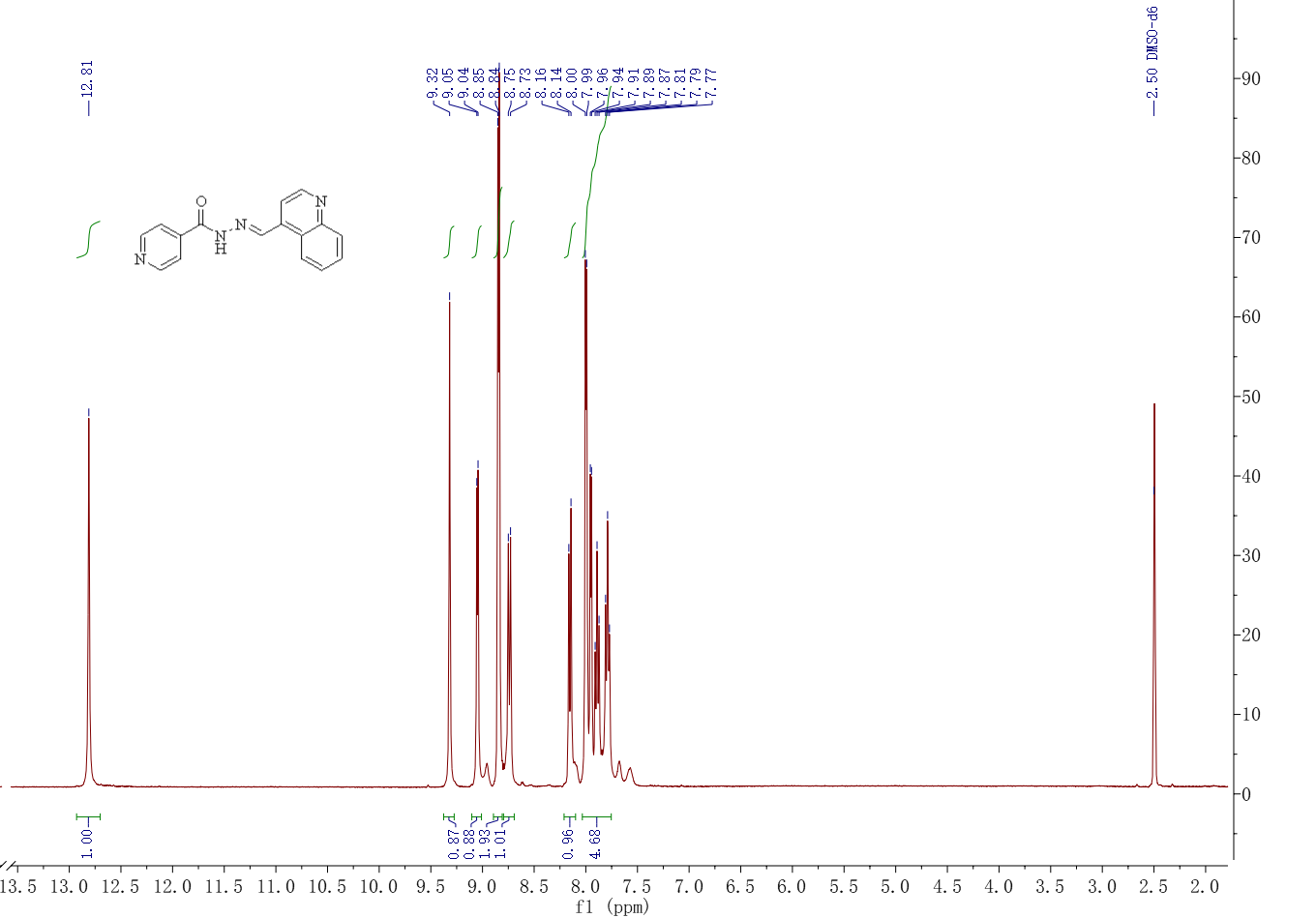


Fig. *^1^H NMR of* **C14** (400 MHz, DMSO)


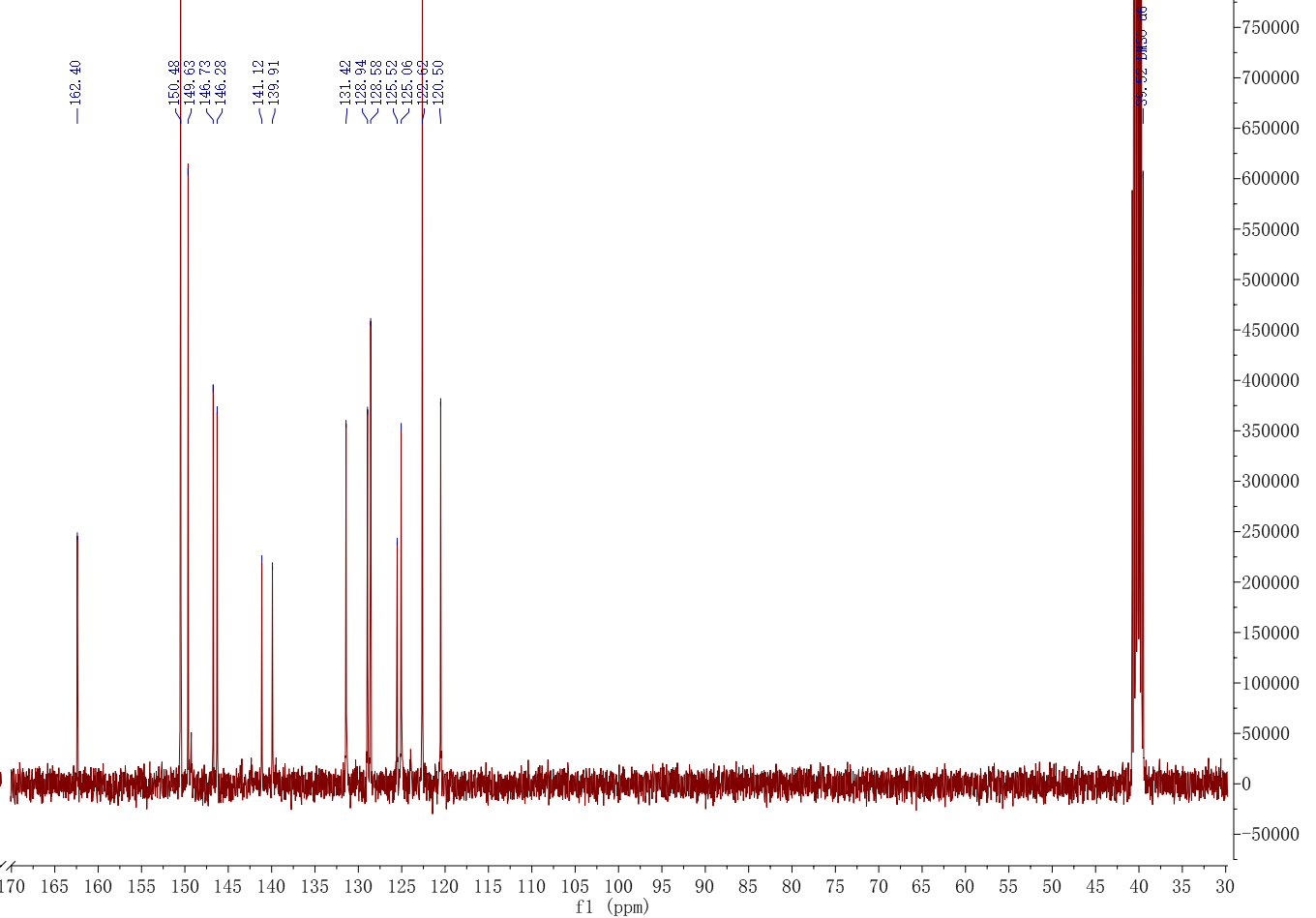


Fig. *^13^C NMR of* **C14** (100 MHz, DMSO)


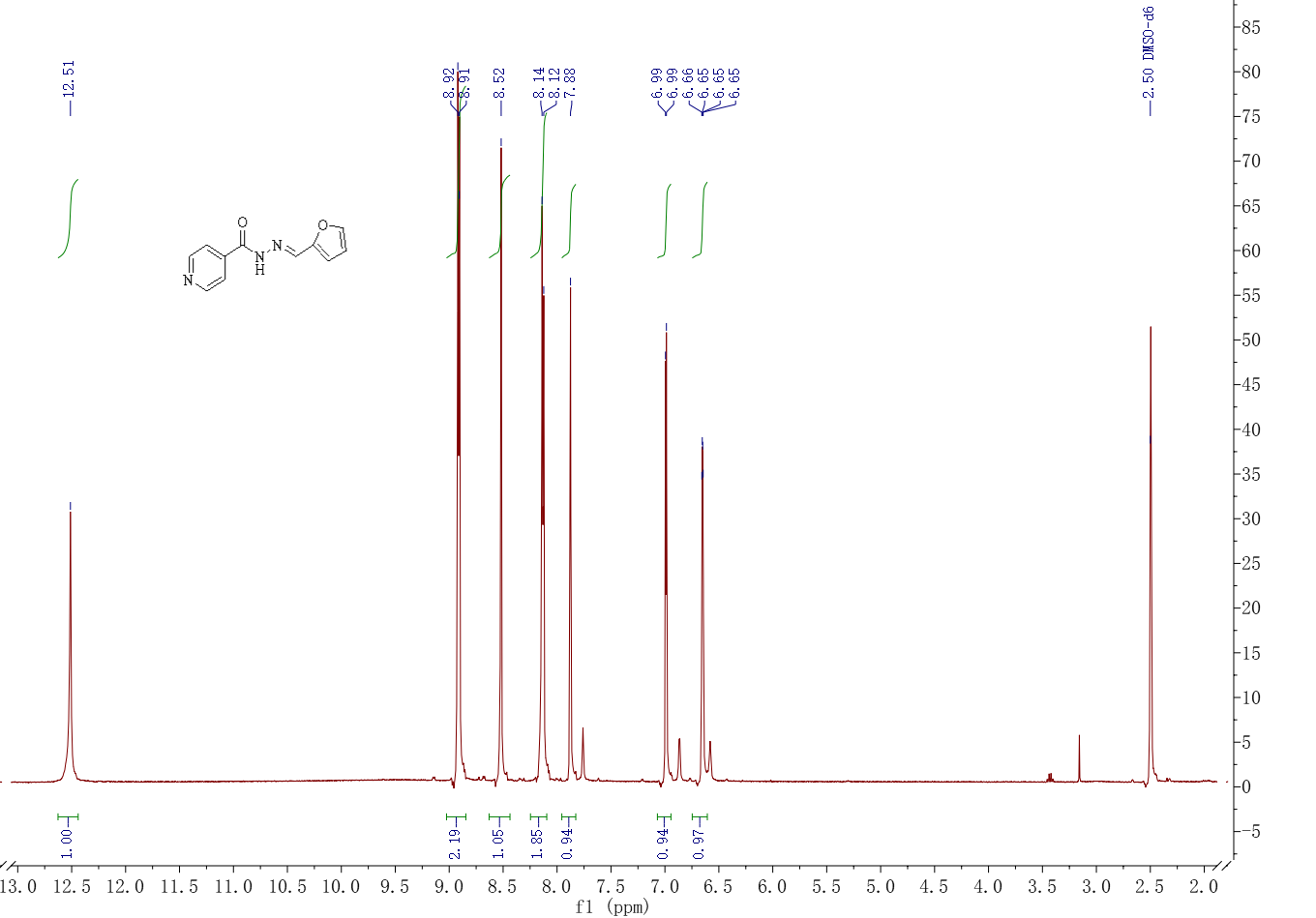


Fig. *^1^H NMR of* **C15** (400 MHz, DMSO)


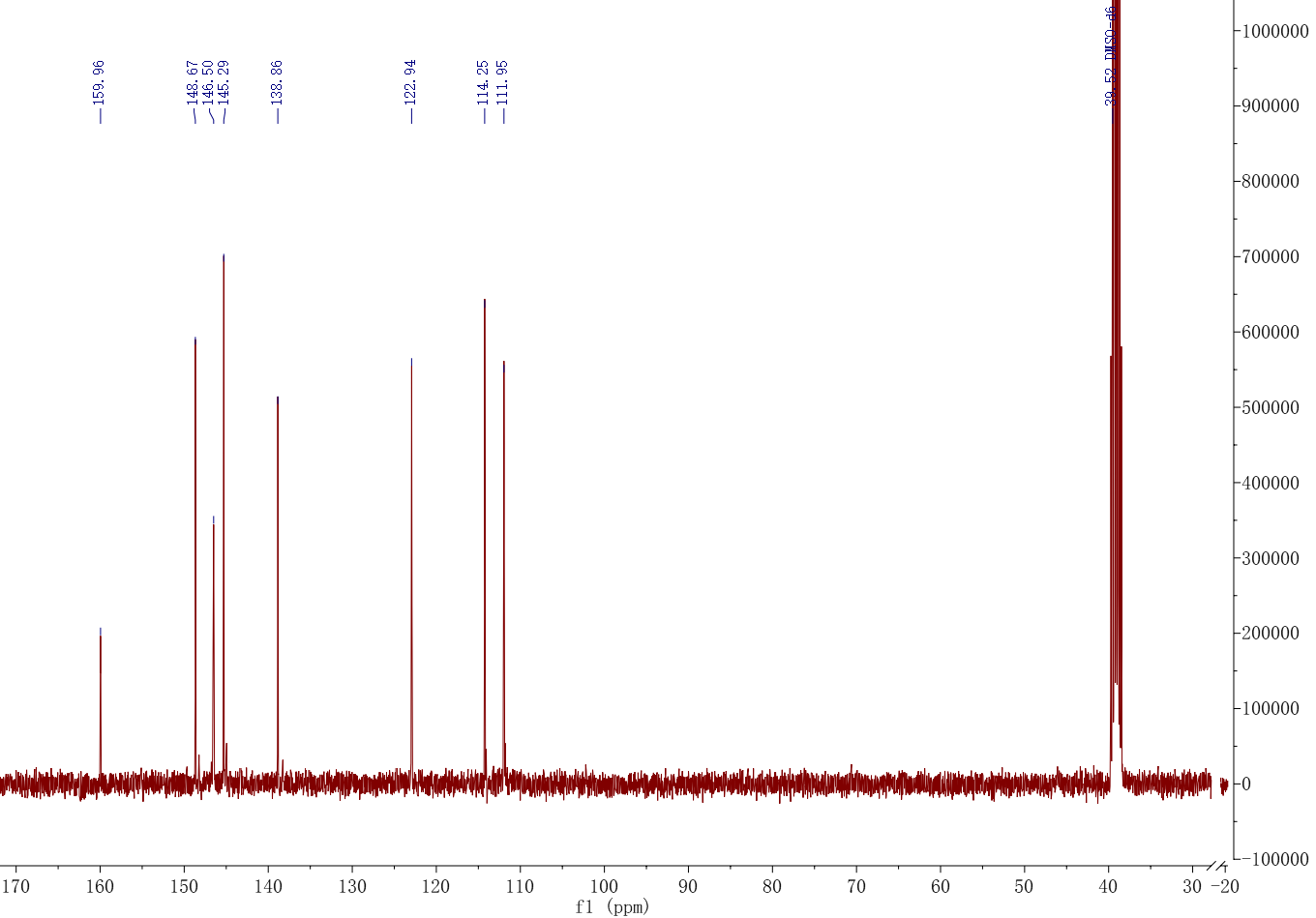


Fig. *^13^C NMR of* **C15** (100 MHz, DMSO)


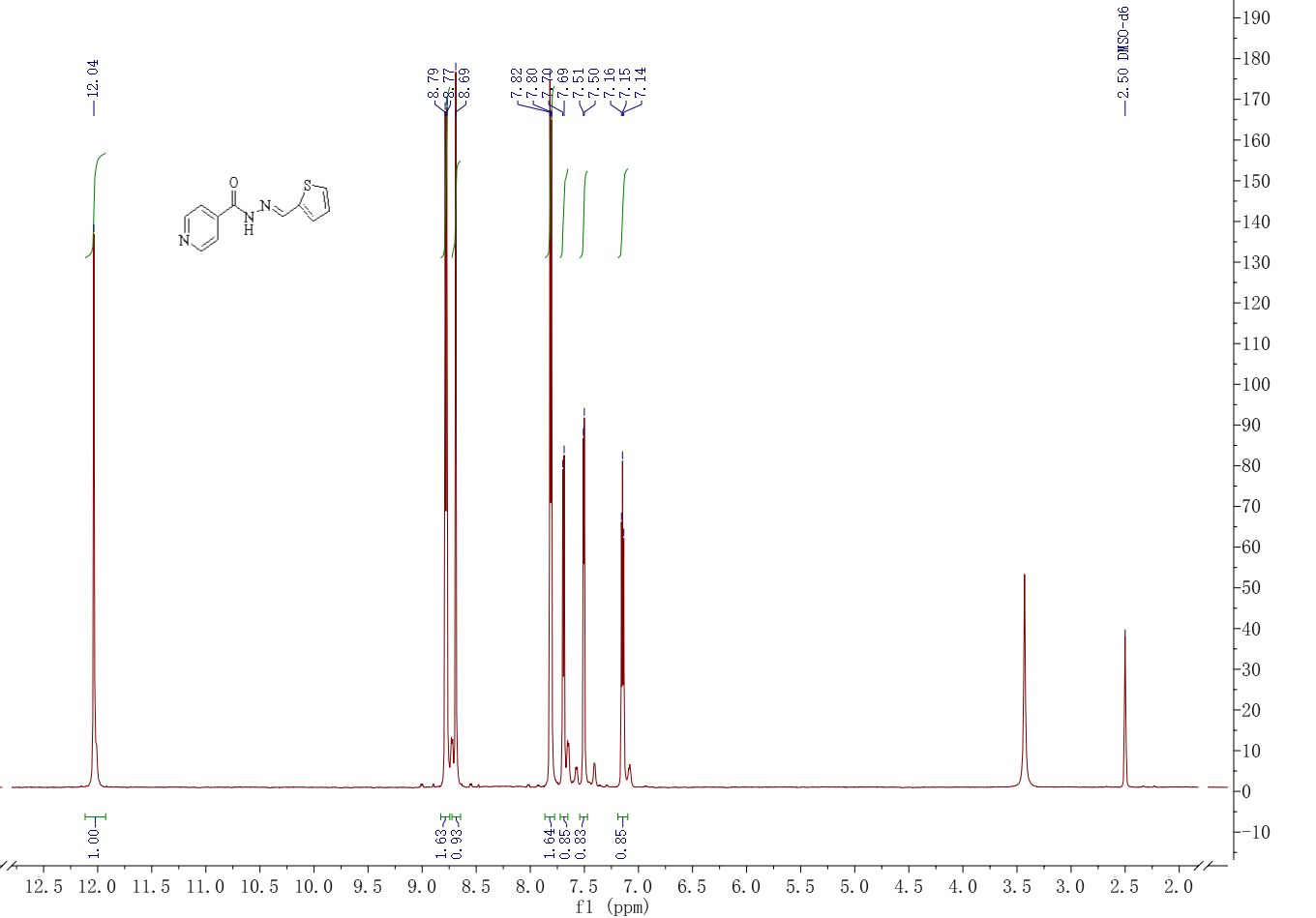


Fig. *^1^H NMR of* **C16** (400 MHz, DMSO)


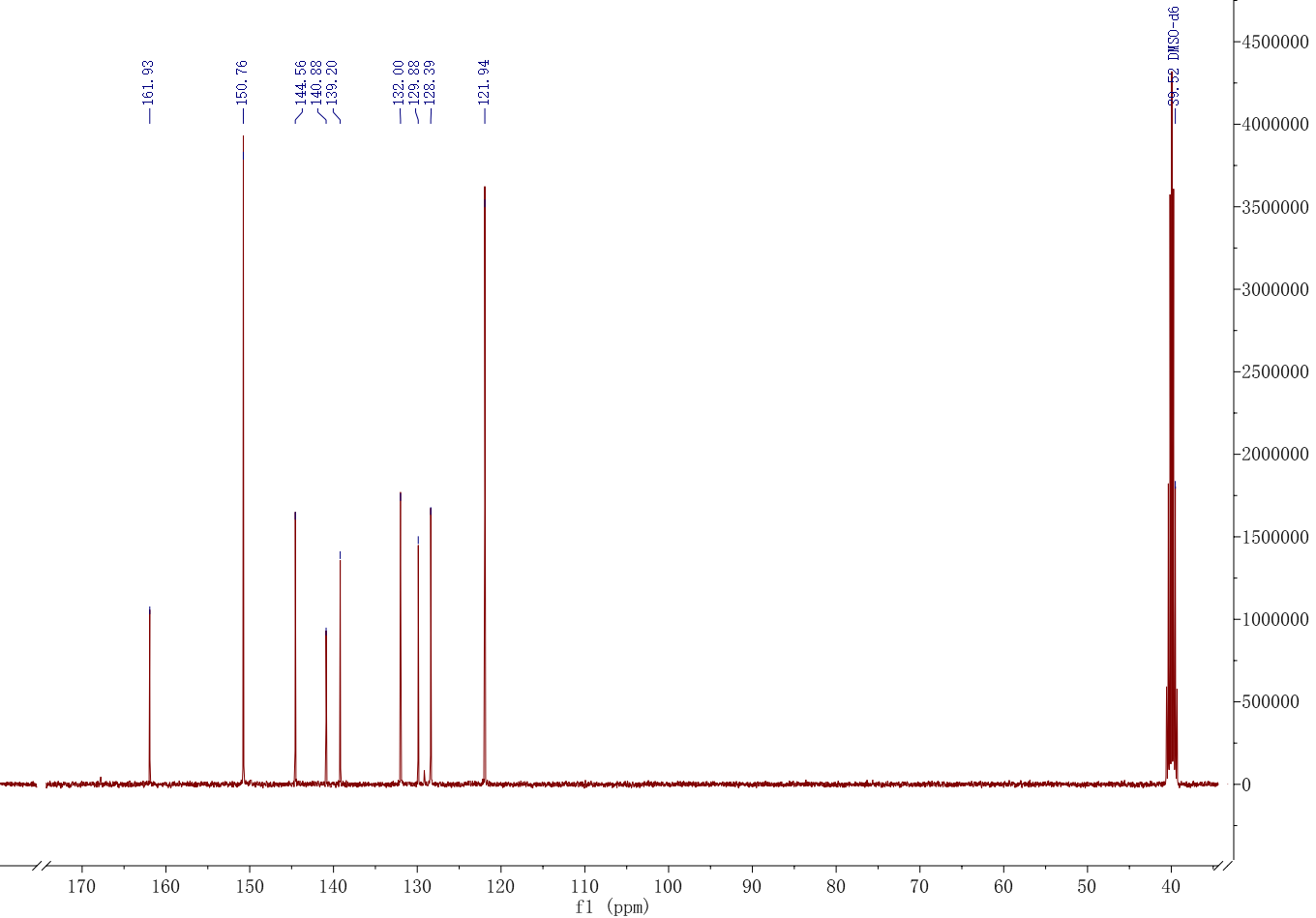


Fig. *^13^C NMR of* **C16** (100 MHz, DMSO)

1. *Correspondent. E-mail: [h418561754@163.com](mailto:h418561754@163.com) [↑](#footnote-ref-2)
